# QuEChERS-Based Multiresidue Pesticide Analysis in Minor Livestock Products and Other Animal-Derived Foods: Performance Evaluation in Selected Matrices and a Market Survey in Korea

**DOI:** 10.3390/molecules31213991

**Published:** 2026-11-05

**Authors:** Min-Ho Song, Yoon-Hee Lee, Yong-Won Cho, Eun-Song Choi, Jung-Hoon Lee, Tae-Gyu Min, Da-Geon Lee, Won-Geun Oh, Jae-Lim Seo, Min-Young Lee, Ji-Ho Lee, Yongho Shin

**Affiliations:** 1 Department of Biological and Environmental Chemistry, College of Agriculture and Life Sciences, Kangwon National University, Chuncheon 24341, Republic of Korea; minobel@naver.com (M.-H.S.);; 2 Department of Applied Bioscience, Dong-A University, Busan 49315, Republic of Korea; 2377652@donga.ac.kr (Y.-H.L.);; 3 Department of Food Biotechnology and Environmental Science, Kangwon National University, Chuncheon 24341, Republic of Korea

**Keywords:** pesticide residues, minor livestock products, QuEChERS, LC–MS/MS, GC–MS/MS, method validation, dietary exposure assessment

## Abstract

A common quick, easy, cheap, effective, rugged, and safe (QuEChERS) procedure coupled with liquid chromatography–tandem mass spectrometry (LC–MS/MS) and gas chromatography–tandem mass spectrometry (GC–MS/MS) was evaluated and applied to survey pesticide residues in minor livestock products and other animal-derived foods marketed in Korea. Performance was assessed in lamb, duck meat, quail egg, mealworm, and edible frog for 150 LC–MS/MS and 75 GC–MS/MS target analytes, including metabolites and isomers. In each matrix, limits of quantitation (LOQs) were ≤0.01 mg/kg for at least 96% of target analytes on each platform. Most analytes met the linearity criterion, and most with LOQs at or below each fortification level met recovery and precision criteria; recurrent low recoveries were most evident for some LC–MS/MS analytes in lamb. Among 90 market samples from 13 food categories, four target analytes were quantified, each in a different sample. Under adult intake assumptions for the surveyed foods, including surrogate and default values, chronic exposure estimates for the four detected pesticides were below their acceptable daily intakes, and acute estimates for the two pesticides with established acute reference doses were below these values. This study provides performance data for the directly evaluated matrices and commodity-specific residue data for the surveyed foods.

## 1. Introduction

Pesticide residues in foods of animal origin can arise from pesticides applied to feed crops, direct treatment of livestock, or treatment of livestock premises [1]. Monitoring provides data on the occurrence and concentrations of pesticide residues in foods for use in dietary exposure assessment together with food-consumption data [2]. European surveillance has generated pesticide residue data for animal products including bovine muscle and fat, poultry, and eggs [3]. A Korean market survey examined beef, pork, chicken, eggs, and milk [4]. Outside these conventional livestock commodities, residues have also been quantified in mealworms from commercial farms in Korea [5], edible insect products marketed in Japan [6], and fresh edible snails [7]. Taken together, the established routes of livestock exposure and the documented occurrence of pesticide residues across conventional and other animal-derived foods support extending occurrence surveys to minor livestock products and other animal-derived foods to broaden commodity-specific residue data for products marketed in Korea.

Reliable occurrence data depend on extraction, cleanup, and quantitation appropriate to the matrix and target analytes. The quick, easy, cheap, effective, rugged, and safe (QuEChERS) approach combines acetonitrile extraction, salt-induced partitioning, and dispersive solid-phase extraction for multiresidue sample preparation [8,9]. Its relatively simple extraction and cleanup steps provide a practical framework for multiresidue analysis, although analytical performance must be established for each analyte–matrix combination. Animal-derived matrices can require different preparation strategies: Lichtmannegger et al. (2015) used modular QuEChERS-derived cleanup schemes according to fat content, whereas Lee et al. (2022) observed fewer compounds meeting recovery and precision criteria in tallow and lard than in beef, pork, and chicken under acidified acetonitrile extraction and C18 cartridge cleanup [10,11]. Analyte properties also influence condition selection; an acetonitrile–ethyl acetate mixture was selected for 17 pyrethroids after solvent-comparison experiments in pork and eggs [12], and primary–secondary amine (PSA) cleanup produced lower recoveries for several acidic analytes than C18 cleanup in mealworms [5]. Matrix-matched calibration was also used in these studies to support quantitation [5,11,12]. Liquid chromatography–tandem mass spectrometry (LC–MS/MS) and gas chromatography–tandem mass spectrometry (GC–MS/MS) can further extend analytical scope through different analyte panels; in hen eggs, 365 LC-amenable and 142 GC-amenable residues were analyzed with a modified QuEChERS procedure [13]. Taken together, these studies illustrate how multiresidue analysis of animal-derived foods has been addressed through matrix- and analyte-dependent choices in sample preparation, matrix-matched quantitation, and complementary LC–MS/MS and GC–MS/MS coverage [14].

A common QuEChERS-based procedure has been evaluated in goat and lamb muscle for pesticides and other contaminants, with performance differing between the two matrices [15], while broad multiresidue methods have been validated in hen eggs and mealworms [5,13]. More recently, a modified QuEChERS–LC–MS/MS method targeting 25 pesticides was applied to eight animal-derived food types in a study that also included residue monitoring and dietary risk assessment [16]. These studies provide performance data for the matrix–analyte combinations and preparation conditions examined. The supported quantitative scope may also differ from the full target panel: in a GC–MS/MS study of mealworms, not all target analytes met recovery criteria at both evaluated fortification levels [17]. Existing performance data therefore provide a foundation for further application but do not, by themselves, establish quantitative suitability for the combinations selected for another survey. For a survey encompassing meat, eggs, edible insects, and amphibians, direct evaluation in selected matrices under common preparation conditions is needed to identify combinations that meet analytical requirements and those requiring caution. The resulting performance data support interpretation of measurements within the directly validated scope, whereas broader market application provides complementary, commodity-specific residue information for foods marketed in Korea.

Residue concentrations alone cannot determine dietary risk because they must be combined with food-consumption data to estimate dietary exposure, which is then compared with relevant health-based guidance values [18]. For pesticide residues, chronic exposure is evaluated against the acceptable daily intake (ADI), whereas acute exposure is evaluated against the acute reference dose (ARfD), where applicable [3]. These exposure estimates are conditional on the residue and consumption data used and on how incomplete information is handled. For residue data, the concentrations underlying non-detected or non-quantified results are unknown, and the values assigned to these results can influence exposure estimates, particularly when a large proportion of observations is left-censored [19]. For consumption data, exposure estimates depend on the coverage and representativeness of the underlying surveys, and assumptions applied when commodity-specific information is incomplete should be stated explicitly [18]. Linking residue surveys with clearly defined exposure assessments therefore places residue data in dietary context while keeping interpretation tied to the foods and population evaluated and the assumptions applied.

This study was undertaken to evaluate a common QuEChERS sample preparation procedure coupled with LC–MS/MS and GC–MS/MS and to investigate pesticide occurrence in animal-derived foods marketed in the Republic of Korea. Analytical performance was assessed in five matrices—lamb, duck meat, quail egg, mealworm, and edible frog—for 150 LC–MS/MS and 75 GC–MS/MS target analytes, including relevant metabolites and isomers. The analytical approach was applied to 90 monitoring samples spanning 13 food categories. The resulting residue data were subsequently used to estimate chronic dietary exposure to the detected pesticides in adults and characterize associated risks from the surveyed foods under the stated assumptions, with acute risk assessment restricted to compounds with established acute reference doses.

## 2. Results and Discussion

### 2.1. Limits of Quantitation

Matrix-specific limits of quantitation (LOQs) ranged from 0.001 to 0.10 mg/kg for LC–MS/MS and from 0.006 to 0.04 mg/kg for GC–MS/MS. Table 1 summarizes the LOQ distribution in each matrix; values for individual analytes are listed in Table S1 (LC–MS/MS) and Table S2 (GC–MS/MS). Depending on the matrix, 145–147 of the 150 LC–MS/MS analytes (96.7–98.0%) and 72–75 of the 75 GC–MS/MS analytes (96.0–100.0%) had LOQs of ≤0.01 mg/kg. Seven LC–MS/MS analytes exceeded this level in at least one matrix: afidopyropen, cyromazine, dichlorvos, flumethrin, flumioxazin, imidacloprid-olefin, and trichlorfon. Trichlorfon had an LOQ of 0.04 mg/kg in all five matrices, and the highest LC–MS/MS LOQ, 0.10 mg/kg, was obtained for flumethrin (quail egg and mealworm) and imidacloprid-olefin (lamb and quail egg). For GC–MS/MS, only three analytes had LOQs above 0.01 mg/kg: phosmet, triadimenol, and triazophos. The highest GC–MS/MS LOQ, 0.04 mg/kg, was obtained for phosmet (lamb, quail egg, and edible frog) and triazophos (quail egg).

In each matrix, LOQs of ≤0.01 mg/kg were obtained for nearly all analytes. Lichtmannegger et al. (2015) investigated more than 80 pesticides in egg and meat using GC–MS/MS and LC–MS/MS, with 0.01 mg/kg used as the LOQ-level fortification, and Kang et al. (2020) reported GC–MS/MS LOQs of 0.00074–0.0231 mg/kg for 66 pesticides in major livestock products [4,10]. Together, these studies support the use of 0.01 mg/kg as a practical sensitivity benchmark for multiresidue analysis in foods of animal origin. Under Regulation (EC) No. 396/2005, a default EU MRL of 0.01 mg/kg generally applies to covered pesticide–commodity combinations for which no specific MRL has been established, subject to the Regulation’s exemptions and alternative default values [20]. Attaining LOQs at or below this level for most analytes is therefore a prerequisite for compliance monitoring at low regulatory thresholds. Notably, this performance was achieved in duck meat, quail egg, and edible frog, for which directly comparable wide-scope pesticide validation data remain limited. In mealworm, the common workflow yielded LOQs of ≤0.01 mg/kg for nearly all analytes without the hexane partitioning or acidified extraction steps used in earlier mealworm-specific methods [5,17]. The method thus demonstrated applicability across compositionally diverse animal-derived matrices.

### 2.2. Linearity

Matrix-matched calibration curves yielded *r*^2^ values of 0.9561–1.0000 for LC–MS/MS and 0.9780–1.0000 for GC–MS/MS. Table 2 summarizes the matrix-specific distributions, and individual values are provided in Tables S1 and S2. Depending on the matrix, 140–149 of 150 LC–MS/MS analytes (93.3–99.3%) and 74–75 of 75 GC–MS/MS analytes (98.7–100.0%) had *r*^2^ ≥ 0.990. All analyte–matrix combinations met the acceptance criterion of *r*^2^ ≥ 0.980 except seven for LC–MS/MS and one for GC–MS/MS. The LC–MS/MS exceptions were flumethrin and flumioxazin in lamb, triforine and imidacloprid-olefin in duck meat, and afidopyropen, dichlorvos, and imidacloprid-olefin in edible frog; the single GC–MS/MS exception was dimethenamid in lamb (*r*^2^ = 0.9780). These eight analyte–matrix combinations did not satisfy the predefined linearity criterion and therefore should not be regarded as quantitatively validated in their respective matrices; none of the four pesticides quantified in the market survey was among the analytes concerned. High calibration linearity has also been reported for 365 LC-amenable and 142 GC-amenable pesticide residues in hen eggs and for 47 pesticides in edible insects [13,21]. However, differences in analyte coverage, calibration ranges, and validation criteria preclude direct ranking of overall method performance.

### 2.3. Recovery and Precision Across Matrices

Accuracy and precision were evaluated by fortifying blank matrices at 0.01, 0.02, and 0.1 mg/kg with five replicates at each level. Table 3 summarizes the numbers of analytes meeting the acceptance criteria in each matrix, and individual recoveries and relative standard deviations (RSDs) are listed in Tables S1 and S2. Depending on the matrix and fortification level, 134–150 LC–MS/MS analytes (89.3–100.0%) and 72–75 GC–MS/MS analytes (98.7–100.0%) met the criteria. For LC–MS/MS, compliance was lowest in lamb, with corresponding proportions of 95.9, 92.5, and 89.3% at 0.01, 0.02, and 0.1 mg/kg, respectively, whereas 96.6–100.0% of the analytes met the criteria in duck meat, quail egg, mealworm, and edible frog at every level.

Broadly acceptable recovery and precision were obtained across the five matrices using a common QuEChERS-based workflow. Other wide-scope studies have also reported substantial proportions of analytes meeting their respective recovery and precision criteria. In hen egg, acceptable recoveries of 70–120% with RSDs of ≤20% were achieved for 92.9% of LC-amenable and 86.6% of GC-amenable pesticide residues [13]. Likewise, more than 90% of the 353 pesticides evaluated in mealworm met the same recovery and precision limits [5]. Sun et al. (2025) evaluated a 25-pesticide QuEChERS–LC–MS/MS method and applied it to eight animal-derived food types [16]. The present recovery and precision results complement that work by documenting performance for 225 target analytes across five selected matrices using both LC–MS/MS and GC–MS/MS. Although direct numerical comparisons are constrained by differences in analyte scope, fortification levels, and acceptance criteria, the present findings support the application of the common extraction and cleanup procedure to analyte–matrix combinations meeting the validation criteria. These results characterize method performance under matrix-matched quantitation; however, matrix effects were not independently quantified, and the magnitude and direction of signal suppression or enhancement could not be determined.

The comparatively lower proportion of LC–MS/MS analytes meeting the criteria in lamb has precedent in broad-scope multiresidue validation. Using the QuEChERSER mega-method covering 330 pesticides, veterinary drugs, and environmental contaminants, Ninga et al. (2022) reported acceptable recovery and precision for 92% of ultra-high-performance liquid chromatography–tandem mass spectrometry (UHPLC–MS/MS) analytes in lamb and 95% in goat; the corresponding low-pressure gas chromatography–tandem mass spectrometry (LPGC–MS/MS) proportions were 82 and 92%, respectively [15]. In the present study, the difference was confined to the LC–MS/MS panel: all GC–MS/MS analytes met the criteria in lamb at every fortification level, and most LC–MS/MS analytes did so as well. Taken together, these observations indicate that lamb was somewhat more demanding than the other matrices examined, consistent with the lamb–goat difference reported by Ninga et al. (2022) [15], but that the effect was limited in scope rather than a general constraint on the analysis of this matrix.

### 2.4. Analyte-Specific Recovery Deviations

Seven LC–MS/MS analytes deviated from the criteria at two or more fortification levels in at least one matrix: bifenazate, cyromazine, dichlorvos, fenhexamid, pyrimethanil-4-hydroxy, pyrimethanil-5-hydroxy, and saflufenacil (Table S1). All seven showed repeated noncompliance in lamb; cyromazine, pyrimethanil-5-hydroxy, and saflufenacil were also observed in duck meat, dichlorvos in mealworm, and saflufenacil in edible frog, whereas no analyte deviated at more than one level in quail egg. All repeated deviations involved low recoveries, and six results also exceeded the RSD criterion. The lowest recoveries were obtained in lamb for pyrimethanil-5-hydroxy (0.6–6.4%), pyrimethanil-4-hydroxy (5.4–7.5%), and bifenazate (17.0–19.8%), and low recoveries were also obtained across matrices for cyromazine (39.4–68.1%), dichlorvos (24.7–64.3%), and saflufenacil (49.7–59.0%). For GC–MS/MS, heptachlor in duck meat at 0.1 mg/kg was the only analyte–matrix combination that fell outside the criteria (67.3%, Table S2); all other evaluated analyte–matrix combinations met the criteria.

The repeatedly deviating analytes fell into two groups. The first included several structurally distinct analytes that exhibited particularly low recoveries in lamb. Bifenazate can be oxidized to bifenazate-diazene [22,23]. In lamb, bifenazate recovery was only 17.0–19.8%, whereas separately quantified bifenazate-diazene showed recoveries of 113.3–119.3%; both compounds yielded recoveries of 72.5–111.4% in the other four matrices. Although this pattern is compatible with possible analyte transformation, conversion during sample preparation was not directly investigated. For EU monitoring of plant commodities, the residue definition includes the sum of bifenazate and bifenazate-diazene expressed as bifenazate [24].

Both pyrimethanil metabolites contain an aromatic hydroxyl group that is absent from the parent compound. Pyrimethanil-4′-hydroxy (2-(4-hydroxyanilino)-4,6-dimethylpyrimidine) is hydroxylated on the aniline ring, whereas pyrimethanil-5-hydroxy (2-anilino-4,6-dimethylpyrimidin-5-ol) is hydroxylated on the pyrimidine ring [25]. Aromatic hydroxylation may alter redox behavior and interactions with matrix components. In phenolic systems, oxidation can generate electrophilic quinonoid intermediates that may form covalent adducts with food proteins [26], potentially rendering a fraction of the analyte less extractable. Such a route may therefore have contributed to the low recoveries, particularly for pyrimethanil-4′-hydroxy, although it was not directly examined under the present conditions.

Fenhexamid contains a para-hydroxyanilide arrangement comparable to the para-hydroxyanilino motif of pyrimethanil-4′-hydroxy, but additionally bears chlorine substituents ortho and meta to the phenolic hydroxyl [27]. Structural differences, including chlorine substitution, may influence the chemical reactivity of these compounds. However, their contribution to the higher recovery of fenhexamid in lamb (55.4–67.8%) than that of pyrimethanil-4′-hydroxy (5.4–7.5%) was not experimentally evaluated. The particularly low recoveries of these four analytes in lamb may be related to the pro-oxidative environment of heme-rich muscle homogenates [28]. However, neither their relative susceptibility to oxidation nor the contribution of this matrix environment was examined under the present extraction conditions. Losses during extraction or cleanup therefore cannot be excluded.

The second group showed compound-specific patterns that may be partly explained by physicochemical properties [27]. Cyromazine, a highly water-soluble weak base (log P 0.069; water solubility 13,000 mg/L), may partition incompletely into acetonitrile during salting-out, and modified extraction procedures have been used to improve its recovery [29]. Saflufenacil, a weakly acidic uracil herbicide (pK_a_ 4.41), showed uniformly modest recoveries (49.7–79.0%) across all five matrices, consistent with partial retention of acidic analytes by the PSA sorbent used in the dispersive cleanup, a loss that has motivated PSA-free QuEChERS variants for acidic pesticides [30]. For dichlorvos, whose vapor pressure (2100 mPa at 20 °C) is exceptionally high for a pesticide, recovery increased with the fortification level in both deviating matrices (from 24.7 to 61.5% in lamb and from 38.1 to 93.2% in mealworm between 0.01 and 0.1 mg/kg), a pattern consistent with a greater relative impact of analyte loss at lower concentrations. Given its high vapor pressure, volatilization during extraction and cleanup may have contributed, although matrix-dependent effects cannot be excluded. The marginal deviation of heptachlor in duck meat (67.3% at 0.1 mg/kg) is likewise consistent with the behavior of this highly lipophilic organochlorine (log P 5.44): with a comparable acetonitrile-based procedure, heptachlor was recovered at 66% from soybean, a commodity containing approximately 20% fat, together with reduced recoveries of other high-log P organochlorines [31]. For routine quantitative monitoring, analyte–matrix combinations that did not meet the acceptance criteria would require additional confirmation or method optimization before reliable quantitation could be established. This limitation does not preclude application of the common workflow to combinations that met the validation criteria.

### 2.5. Pesticide Residues in Monitoring Samples

**Figure 1:**
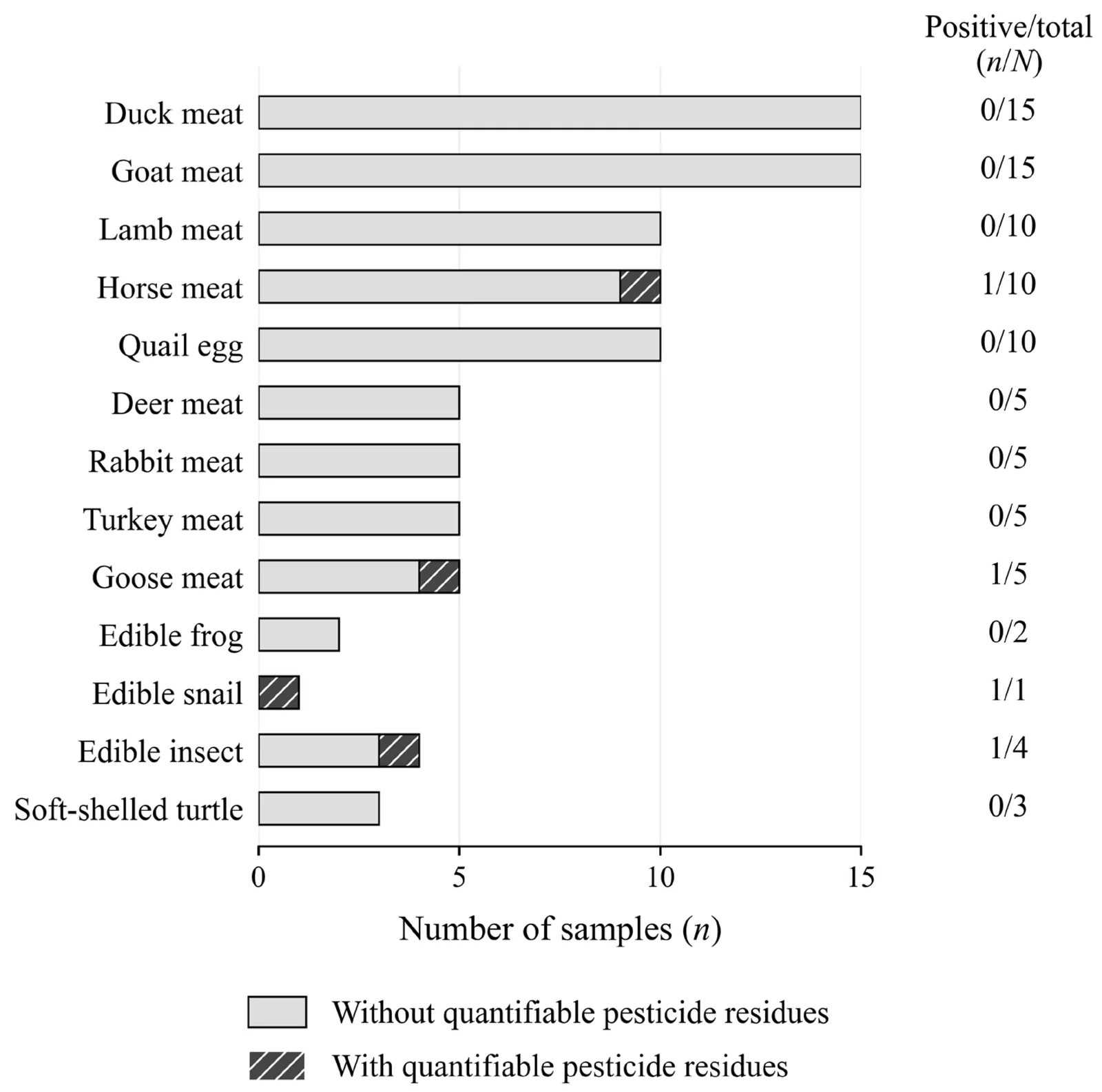
Monitoring sample distribution across 13 food categories. Bar lengths show total sample counts; hatched segments indicate samples with at least one quantifiable target pesticide residue. Labels show positive/total samples (*n*/*N*). Results are exploratory because sample sizes were small and unequal.

Among the 90 monitoring samples representing 13 minor livestock and animal-derived food categories, pesticide residues were quantified in four samples (4.4%). Figure 1 summarizes the numbers of samples examined and those containing quantifiable pesticide residues across the 13 food categories. Bifenthrin was quantified at 0.011 mg/kg in one of the 10 horse meat samples, and flubendiamide at 0.015 mg/kg in one of the five goose meat samples. Lufenuron was quantified at 0.064 mg/kg in one of the four edible insect samples, whereas isoprothiolane was measured at 0.350 mg/kg in the single edible snail sample examined (Table 4). Only one target analyte was quantified in each positive sample, and no target pesticide residues were quantified in the remaining 86 samples. For monitoring samples lacking suitable analyte-free blank matrices, quantitation was performed by post-extraction standard addition using the corresponding cleaned sample extracts. In particular, isoprothiolane in edible snail was quantified using this approach rather than a calibration curve prepared in a surrogate matrix.

The predominance of samples without quantifiable residues was also observed in European monitoring of animal products, in which 93.1% of 19,995 samples contained no quantifiable residues in 2024 [3]. Pesticide residues in beef, pork, chicken, eggs, and milk were investigated in an earlier Korean market survey [4], whereas additional occurrence data for horse and goose meat were obtained in the present study. For edible insects, lufenuron was previously quantified in mealworms collected from commercial farms in Korea [5], providing a direct precedent for its occurrence in this food category. In a separate Japanese market survey, residues of 20 pesticides were quantified across nine of the 50 edible insect products examined [6]. Pesticide residues were quantified in 22.09% of 824 edible snail samples, with 27 different pesticides represented among the positive samples [7]. Both isoprothiolane and lufenuron were also detected in freshwater fish in a nationwide Korean monitoring study [32], documenting their occurrence in other animal-derived foods without establishing a common contamination source. These published findings provide context for the present results, although direct comparisons of detection frequencies and residue concentrations are limited by differences in species, product processing, analytical scope, and LOQs. Within the present survey, reliable comparisons of contamination prevalence among food categories were also limited by the small and unequal sample numbers, particularly the inclusion of only one edible snail sample (Table 4). The measured residue concentrations were subsequently used as inputs to the dietary exposure assessment.

### 2.6. Dietary Exposure and Risk Characterization

Under the specified intake assumptions, estimated chronic dietary exposures from the monitored commodities were below the respective ADIs for all four detected pesticides in both the lower-bound (LB) and upper-bound (UB) scenarios (Table 5). The highest commodity-specific %ADI values were obtained for flubendiamide in goose meat, at 0.190% (LB) and 0.242% (UB), followed by bifenthrin in horse meat, at 0.0169% and 0.108%, respectively. The UB estimates for lufenuron and isoprothiolane corresponded to 9.04 × 10^−4^% and 1.36 × 10^−4^% of their respective ADIs. Both UB exposure estimates were reported as 1.36 × 10^−7^ mg/kg b.w./day after rounding to three significant figures. Nevertheless, the UB %ADI for lufenuron was approximately 6.65 times that for isoprothiolane, primarily reflecting the difference between their ADIs of 0.015 and 0.100 mg/kg b.w./day, respectively.

The similar UB exposure estimates for lufenuron and isoprothiolane were largely explained by the summation term for the remaining monitored commodities in Equation (2), in which unquantified residue concentrations were replaced by the corresponding assigned LOQs. This term accounted for 99.99% and 99.79% of the total UB estimates, respectively. For both compounds, approximately 91.4% of the summation term was attributed to duck and goose meat, for which the same mean-intake and LOQ values were used (Tables S1 and S3). By contrast, 91.1% and 99.2% of the increases from LB to UB for bifenthrin and flubendiamide were attributed to LOQ substitution for unquantified residues within the horse- and goose-meat categories, respectively. For lufenuron and isoprothiolane, essentially the entire LB–UB increase was attributed to the summation term. For isoprothiolane, the mean concentration in edible snail was identical in both scenarios because the compound was quantified in the only sample examined (Table 4). For edible insects and snails, a common default intake of 0.00005 g/day was assigned to both *IR_mean_* and *IR_extreme_* as an imputation assumption (Table S3). These variables denote the mean- and extreme-intake inputs, respectively. Accordingly, these UB estimates were interpreted as conditional on the assigned intakes and the treatment of left-censored concentrations, rather than as upper bounds on actual high-consumer exposure.

In the acute assessment, maximum measured concentrations were used, so the estimates were unaffected by the LB–UB substitution applied to unquantified residues in the chronic assessment. The modified global estimate of acute dietary exposure (GEADE) yielded values of 1.69 × 10^–5^ and 1.62 × 10^–4^ mg/kg b.w./day for bifenthrin in horse meat and flubendiamide in goose meat, corresponding to 0.169% and 0.0809% of their respective ARfDs (Table 6). For these two compounds, the ranking by chronic %ADI was reversed for acute %ARfD: The acute exposure estimate for flubendiamide was approximately 9.6 times that for bifenthrin. However, its ARfD (0.200 mg/kg b.w.) was 20 times that of bifenthrin (0.010 mg/kg b.w.). No %ARfD values were calculated for lufenuron or isoprothiolane, as ARfD establishment was considered unnecessary in the 2015 and 2017 JMPR evaluations, respectively [33,34]. For horse meat, the consumers-only mean intake for lamb meat was used, whereas the 97.5th-percentile intake among duck-meat consumers was used for goose meat (Table S3). Although both acute exposure estimates were below their respective ARfDs under these surrogate-intake assumptions, the horse-meat estimate was not based on a 97.5th-percentile consumption input.

Taken together, the monitoring data were used to characterize pesticide-specific dietary exposure from the surveyed minor livestock and animal-derived foods under the stated assumptions. The assessment was restricted to the monitored commodities; exposure to the same pesticides through foods outside this survey was not included. Estimates were calculated using an assumed adult body weight of 60 kg, and separate assessments were not performed for other age groups. Further refinements could incorporate commodity-specific consumption data, direct verification of analytical performance for commodities assigned surrogate or default LOQs, and more representative residue monitoring. These improvements would reduce reliance on surrogate and default inputs.

## 3. Materials and Methods

### 3.1. Chemicals and Reagents

Analytical standards and stock solutions (100–1000 mg/L) of the target pesticides, including relevant metabolites and isomers, were purchased primarily from AccuStandard, Inc. (New Haven, CT, USA) and HPC Standards GmbH (Jesewitz, Germany), with additional standards obtained from Sigma-Aldrich Co. (St. Louis, MO, USA), Dr. Ehrenstorfer GmbH (Augsburg, Germany), Chem Service, Inc. (West Chester, PA, USA), LGC Standards GmbH (Luckenwalde, Germany), and Kemidas Co., Ltd. (Gunpo, Republic of Korea). HPLC-grade acetonitrile and LC–MS-grade methanol were obtained from Duksan Pure Chemicals Co., Ltd. (Ansan, Republic of Korea) and Thermo Fisher Scientific Inc. (Waltham, MA, USA), respectively. Optima LC/MS-grade formic acid and BioUltra ammonium formate solution (10 M in water) were purchased from Thermo Fisher Scientific Inc. and Sigma-Aldrich Co., respectively. Extra-pure anhydrous magnesium sulfate and guaranteed-reagent-grade sodium chloride were obtained from Daejung Chemicals & Metals Co., Ltd. (Siheung, Republic of Korea) and Junsei Chemical Co., Ltd. (Tokyo, Japan), respectively. Dispersive solid-phase extraction (d-SPE) tubes containing 150 mg MgSO_4_, 25 mg PSA, and 25 mg C18 were purchased from TaeSan Science Co., Ltd. (Anyang, Republic of Korea). LC–MS-grade water was obtained from Merck KGaA (Darmstadt, Germany).

### 3.2. Preparation of Working Standards and Calibration Procedures

Individual stock solutions (100–1000 mg/L) were used as supplied or prepared in methanol or acetonitrile, as appropriate. The individual stock solutions were combined separately to prepare mixed working standard solutions for LC–MS/MS and GC–MS/MS analyses. Each mixture was diluted with acetonitrile to 0.5, 1, 2, 3, 5, 10, 20, 50, 80, 200, 500, 1000, and 5000 ng/mL. Matrix-matched calibration standards were prepared separately for each validation matrix by mixing equal volumes of cleaned blank matrix extract and the appropriate working standard solution (1:1, *v*/*v*). For LC–MS/MS, matrix-matched calibration curves ranged from the analyte-specific limit of quantitation (LOQ) to 25 or 40 ng/mL, equivalent to 0.10 or 0.16 mg/kg in the samples. For GC–MS/MS, the calibration range extended from the analyte-specific LOQ to 25 ng/mL, equivalent to 0.10 mg/kg. For monitoring samples for which suitable analyte-free blank matrices were unavailable, quantitation was performed using post-extraction standard addition. Known amounts of the target analyte were added to aliquots of the cleaned sample extracts prior to instrumental analysis, and the residue concentration was determined from the resulting standard-addition calibration relationship.

### 3.3. Sample Preparation

A 5.0-g portion of each homogenized sample was placed in a 50-mL centrifuge tube and extracted with 10 mL of acetonitrile at 1300 rpm for 2 min using a 1600 MiniG shaker (SPEX SamplePrep, Metuchen, NJ, USA). A QuEChERS extraction salt packet containing 4 g of anhydrous magnesium sulfate and 1 g of sodium chloride was added, and the tube was shaken at 1300 rpm for 1 min using the same instrument. The extract was centrifuged at 3500 rpm for 10 min using a ScanSpeed 1248 centrifuge (LaboGene A/S, Allerød, Denmark). A 1-mL aliquot of the supernatant was transferred to a 2-mL d-SPE tube. After mixing for 1 min, the tube was centrifuged at 13,000 rpm for 5 min at 4 °C using an M15R centrifuge (Hanil Scientific Inc., Gimpo, Republic of Korea). The cleaned supernatant was mixed with acetonitrile at a 1:1 (*v*/*v*) ratio and subjected to LC–MS/MS and GC–MS/MS analyses. Sample extracts exceeding the calibration range were further diluted and reanalyzed, and concentrations were corrected for the additional dilution. No freezing-out step was performed during extraction or cleanup.

### 3.4. Method Validation

Method validation was performed separately for the lamb, duck meat, quail egg, mealworm, and frog matrices for both LC–MS/MS and GC–MS/MS analyses with reference to Codex CXG 40-1993 [35]. LOQ, linearity, recovery, and within-laboratory precision were evaluated. The LOQ was defined as the lowest concentration producing a signal-to-noise ratio (S/N) of ≥10. Linearity was assessed using matrix-matched calibration curves, and an *r*^2^ value of ≥0.980 was considered acceptable. Accuracy and precision were assessed by fortifying blank samples before extraction at 0.01, 0.02, and 0.1 mg/kg, with five replicates at each level. Accuracy was expressed as mean recovery, and precision was expressed as the RSD. Acceptance criteria were 60–120% recovery with an RSD of ≤32% at 0.01 mg/kg and 70–120% recovery with an RSD of ≤22% at 0.02 and 0.1 mg/kg.

### 3.5. Sample Procurement

Lamb meat, duck meat, quail eggs, mealworms (*Tenebrio molitor* larvae), and edible frog samples used for method validation were purchased from local retail markets. Candidate samples from each of the five validation matrices were screened for the target pesticides using the LC–MS/MS and GC–MS/MS procedures described in Section 3.6. Samples in which no target pesticides were detected were selected as blank matrices for matrix-matched calibration and recovery experiments. A total of 90 monitoring samples representing 13 minor livestock and animal-derived food categories were purchased throughout the Republic of Korea between 8 July and 16 September 2025 (Table S3). The samples comprised duck meat (*n* = 15), goat meat (*n* = 15), lamb meat (*n* = 10), horse meat (*n* = 10), quail eggs (*n* = 10), deer meat (*n* = 5), rabbit meat (*n* = 5), turkey meat (*n* = 5), goose meat (*n* = 5), edible frog samples (*n* = 2), an edible snail sample (*n* = 1), edible insect samples (*n* = 4), and soft-shelled turtle samples (*n* = 3). Offline purchasing was prioritized, whereas online markets were used for commodities with limited local availability; 69 samples were obtained offline and 21 online. Each sample was assigned a unique identification code. Upon arrival at the laboratory, all samples were immediately homogenized by dry-ice-assisted grinding and stored at −20 °C until extraction.

### 3.6. Instrumental Analysis

Pesticides assigned to LC–MS/MS were analyzed using an LCMS-8050 system (Shimadzu Corp., Kyoto, Japan). Chromatographic separation was performed on a Kinetex C18 column (150 × 2.1 mm i.d., 2.6 μm; Phenomenex, Inc., Torrance, CA, USA) using water (A) and methanol (B), each containing 0.1% formic acid and 5 mM ammonium formate. The gradient program was 5% B at 0–0.2 min, 50% B at 0.5 min, 98% B at 16.5–19.5 min, and 5% B at 19.6–23.0 min. The flow rate was 0.2 mL/min, and the injection volume was 2 μL. Electrospray ionization was performed in positive- and negative-ion modes. The nebulizing, heating, and drying gas flow rates were 3.0, 10.0, and 10.0 L/min, respectively. The interface, desolvation-line, and heat-block temperatures were 300, 250, and 400 °C, respectively. Argon was used as the collision gas.

Pesticides assigned to GC–MS/MS were analyzed using a GCMS-TQ8050 NX system (Shimadzu Corp., Kyoto, Japan) equipped with an Rtx-5MS capillary column (30 m × 0.25 mm i.d., 0.25 μm film thickness; Restek Corporation, Bellefonte, PA, USA). Helium was used as the carrier gas at 1.2 mL/min. The oven temperature was initially set at 90 °C, increased to 180 °C at 20 °C/min and then to 300 °C at 5 °C/min, and held at 300 °C for 5 min. A 2-μL aliquot was injected in splitless mode, and the interface temperature was maintained at 300 °C. For both analytical platforms, the target analytes were monitored in multiple reaction monitoring mode using analyte-specific quantifier and qualifier transitions and collision energies (Tables S4 and S5).

### 3.7. Dietary Exposure and Risk Assessment

#### 3.7.1. Chronic Dietary Exposure and Risk Characterization

Chronic dietary exposure was estimated by combining the residue concentrations measured in the present monitoring survey with food intake values compiled in the source monitoring report, which used data from the eighth Korea National Health and Nutrition Examination Survey (KNHANES VIII, 2019–2021) [36]. The estimate was based on the global estimate of chronic dietary exposure (GECDE) developed by the Joint FAO/WHO Expert Committee on Food Additives (JECFA), which was adapted to the present dataset as described below [37,38]. Although originally developed for veterinary drug residues, the GECDE framework was adapted here for a scenario-based assessment of the monitored commodities using the intake assignments described below. For each detected compound, the commodity-specific exposure term was calculated using Equation (1):

(1) $${\text{High exposure}}_{i}\;(\text{mg}/\text{kg b.w.}/\text{day})=\frac{{IR}_{\text{extreme},i}\;(\text{g}/\text{day})\;\times \;C_{\text{mean},i}\;(\text{mg}/\text{kg})}{BW\;(\text{kg b.w.})}\times 0.001\;(\text{kg}/\text{g})\;$$

where *IR_extreme,i_* denotes the commodity-specific intake input used in Equations (1) and (4). The 97.5th percentile intake among consumers was used where available. For lamb meat, goat meat, and quail eggs, consumers-only mean intakes were used instead, in accordance with the procedure described in the source report [36]. When commodity-specific intake data were unavailable, lamb meat and duck meat were used as surrogates for horse meat and goose meat, respectively. For commodities lacking both intake data and a suitable surrogate, a common default intake of 0.00005 g/day was assigned to both *IR_extreme_* and *IR_mean_*, consistent with the default value tabulated in the monitoring and intake-estimation reports [36,39]. The same default was used because the available data did not support separate estimates of mean and high consumption. This assignment was treated as an imputation assumption rather than an observed intake or a high-consumer percentile. The same surrogate-commodity assignments and *IR_extreme,i_* values were used in the acute assessment described below.

*C_mean,i_* is the mean concentration of the compound across all samples of commodity *i*, including those in which no residue was detected, and *BW* is the assumed body weight. The original method uses the median residue concentration as the representative residue level. In a dataset in which fewer than half of the samples contain quantifiable residues, however, the median becomes zero under the LB scenario or the LOQ under the UB scenario and does not adequately reflect the residues actually measured. The median was therefore replaced by the mean concentration defined above. Because most of the results were left-censored, lower-bound (LB) and upper-bound (UB) scenarios were applied: concentrations below the LOQ were assigned a value of zero in the LB scenario and a value equal to the LOQ in the UB scenario [19]. For the UB calculation, analyte- and matrix-specific LOQs listed in Tables S1 and S2 were assigned according to the following matrix mapping: lamb for goat meat, horse meat, deer meat, and rabbit meat; duck meat for goose meat and turkey meat; mealworm for edible insects; and edible frog for soft-shelled turtle. An LOQ of 0.01 mg/kg was assigned to edible snail because no comparable validated matrix was available. Accordingly, *C_mean,i_* and the corresponding mean concentrations for the remaining commodities (*C_mean,k_*) were calculated separately for each scenario. A body weight of 60 kg was assumed for Korean adults [40], and the assessment was accordingly restricted to the general adult population.

The modified GECDE was calculated using Equation (2):

(2) $$GECDE\;(\text{mg}/\text{kg b.w.}/\text{day})={\text{High exposure}}_{i}+\;\sum_{k\neq i}\frac{{IR}_{\text{mean},\;k}\;(\text{g}/\text{day})\;\times \;C_{\text{mean},k\;}(\text{mg}/\text{kg})}{BW\;(\text{kg b.w.})}\times 0.001\;(\text{kg}/\text{g})$$

where the summation is taken over all remaining commodities *k* (*k* ≠ *i*), and *IR_mean,k_* denotes the population-mean daily intake of commodity *k*, including non-consumers, or its assigned substitute. For horse meat and goose meat, the population-mean intakes of lamb meat and duck meat, respectively, were used. For commodities lacking both intake data and a suitable surrogate, the common default intake of 0.00005 g/day described above was applied. The intake values and assignments used in the assessment are provided in Table S3. In the original method, Equation (2) is evaluated once for each commodity in turn and the highest of the resulting values is reported as the GECDE. For each compound, Equation (2) was evaluated separately for every commodity in which that compound was detected, and the resulting value was reported as a commodity-specific estimate (GECDE*_i_*). Under the LB scenario, commodities in which the compound was not detected contributed zero to the summation term in Equation (2); consequently, only commodities with detected residues contributed to the exposure estimate.

Chronic risk was characterized by comparing the modified GECDE with the acceptable daily intake (ADI) of each compound using Equation (3):

(3) $$\%ADI=\frac{GECDE\;(\text{mg}/\text{kg b.w.}/\text{day})}{ADI\;(\text{mg}/\text{kg b.w.}/\text{day})}\times 100$$

The ADI values were obtained from the Korean regulatory list of pesticide acceptable daily intakes issued by the Rural Development Administration [41]. A %ADI exceeding 100% was regarded as indicative of a potential chronic health concern.

#### 3.7.2. Acute Dietary Exposure and Risk Characterization

Acute dietary exposure was estimated using the GEADE framework defined by JECFA, adapted as described below [37,38]. The high-residue concentration used in the original GEADE framework, generally derived as a 95/95 upper tolerance limit, was replaced by the maximum concentration measured for each analyte–commodity combination, thereby avoiding the need to estimate an underlying residue distribution from the limited monitoring data. The modified GEADE was calculated using Equation (4):

(4) $$GEADE\;(\text{mg}/\text{kg b.w.}/\text{day})=\;\frac{{IR}_{\text{extreme},i}\;(\text{g}/\text{day})\;\times \;C_{\text{max},i}\;(\text{mg}/\text{kg})}{BW\;(\text{kg b.w.})}\;\times \;0.001\;(\text{kg}/\text{g})$$

where *C_max,i_* is the maximum concentration measured among the samples of commodity *i* in which the compound was detected, and *IR_extreme,i_* and *BW* are as defined for Equation (1).

Acute risk was characterized by comparing the modified GEADE with the acute reference dose (ARfD) of each compound using Equation (5):

(5) $$\%ARfD=\frac{GEADE(\text{mg}/\text{kg b.w.}/\text{day})}{ARfD(\text{mg}/\text{kg b.w.}/\text{day})}\times 100$$

The ARfD values were obtained from JMPR evaluations [42,43]. Acute risk was evaluated only for the compounds for which an ARfD has been established, and a %ARfD exceeding 100% was regarded as indicative of a potential acute health concern.

## 4. Conclusions

When a common QuEChERS sample preparation procedure was applied without matrix-specific modification to five matrices (lamb, duck meat, quail egg, mealworm, and edible frog), LOQs of ≤0.01 mg/kg were obtained for at least 96% of the 150 LC–MS/MS and 75 GC–MS/MS target analytes, including metabolites and isomers, in each matrix. Most analytes met the linearity criterion, and most analytes with LOQs at or below each fortification level met the recovery and precision criteria. Performance limitations were identified for certain analyte–matrix combinations, particularly recurrent low recoveries for some LC–MS/MS analytes in lamb; these exceptions indicate that quantitative interpretation should rest on the performance documented for each combination. In the broader survey of 90 market samples from 13 food categories, one target analyte was quantified in each of four samples. Because sample numbers were small and unequal across food categories, the market survey should be considered exploratory rather than representative of pesticide occurrence in the Korean market as a whole. Under the stated assumptions for adult dietary exposure from the surveyed foods, chronic estimates for all four detected pesticides were below their respective acceptable daily intakes, and acute estimates for the two pesticides with established acute reference doses were below those values. These estimates depend on the assumed adult body weight, surrogate or default food intakes, and LOQ substitution in the chronic UB scenario; they do not represent total dietary exposure from all foods. Together, the performance data provide a basis for interpreting quantitative results in the directly evaluated matrices, while the market survey adds commodity-specific residue data for animal-derived foods marketed in Korea.

## Figures and Tables

**Table 1:** Matrix-specific distribution of limits of quantitation and analytes with LOQs above 0.01 mg/kg.

Matrix	LOQ Category (mg/kg), Analytes [*n* (%)]				Analytes with LOQ > 0.01 mg/kg
**LC–MS/MS ^1^**	**≤0.01**	**0.02**	**0.04**	**0.10**	**LOQ (mg/kg): Analyte(s)**
Lamb	145 (96.7)	2 (1.3)	2 (1.3)	1 (0.7)	0.02: Afidopyropen, flumioxazin 0.04: Flumethrin, trichlorfon 0.10: Imidacloprid-olefin
Duck meat	147 (98.0)	1 (0.7)	2 (1.3)	— ^3^	0.02: Flumethrin 0.04: Trichlorfon, imidacloprid-olefin
Quail egg	145 (96.7)	1 (0.7)	2 (1.3)	2 (1.3)	0.02: Afidopyropen 0.04: Dichlorvos, trichlorfon 0.10: Flumethrin, imidacloprid-olefin
Mealworm	146 (97.3)	1 (0.7)	2 (1.3)	1 (0.7)	0.02: Imidacloprid-olefin 0.04: Cyromazine, trichlorfon 0.10: Flumethrin
Edible frog	147 (98.0)	—	3 (2.0)	—	0.04: Flumethrin, trichlorfon, imidacloprid-olefin
**GC–MS/MS ^2^**	**≤0.01**	**0.02**	**0.04**	**0.10**	**LOQ (mg/kg): analyte(s)**
Lamb	73 (97.3)	1 (1.3)	1 (1.3)	—	0.02: Triazophos 0.04: Phosmet
Duck meat	73 (97.3)	2 (2.7)	—	—	0.02: Phosmet, triadimenol
Quail egg	72 (96.0)	1 (1.3)	2 (2.7)	—	0.02: Triadimenol 0.04: Phosmet, triazophos
Mealworm	75 (100.0)	—	—	—	—
Edible frog	74 (98.7)	—	1 (1.3)	—	0.04: Phosmet

^1^ LC–MS/MS: *n* = 150 analytes per matrix. ^2^ GC–MS/MS: *n* = 75 analytes per matrix. ^3^ The symbol “—” indicates that no analytes were included in the corresponding category.

**Table 2:** Matrix-specific distribution of coefficients of determination (*r*^2^).

Matrix	*r*^2^ Category, Analytes [*n* (%)]			Analytes with *r*^2^ < 0.980
**LC–MS/MS** ^1^	**≥0.990**	**0.980–0.990**	**<0.980**	**Analyte(s)**
Lamb	145 (96.7)	3 (2.0)	2 (1.3)	Flumethrin, Flumioxazin
Duck meat	145 (96.7)	3 (2.0)	2 (1.3)	Imidacloprid-olefin, Triforine
Quail egg	146 (97.3)	4 (2.7)	— ^3^	—
Mealworm	149 (99.3)	1 (0.7)	—	—
Edible frog	140 (93.3)	7 (4.7)	3 (2.0)	Afidopyropen, Dichlorvos, Imidacloprid-olefin
**GC–MS/MS** ^2^	**≥0.990**	**0.980–0.990**	**<0.980**	**Analyte(s)**
Lamb	74 (98.7)	—	1 (1.3)	Dimethenamid
Duck meat	74 (98.7)	1 (1.3)	—	—
Quail egg	75 (100.0)	—	—	—
Mealworm	75 (100.0)	—	—	—
Edible frog	75 (100.0)	—	—	—

^1^ LC–MS/MS: *n* = 150 analytes per matrix. ^2^ GC–MS/MS: *n* = 75 analytes per matrix. ^3^ The symbol “—” indicates that no analytes were included in the corresponding category.

**Table 3:** Matrix-specific numbers of analytes meeting the recovery and precision acceptance criteria at each fortification level.

Matrix	Analytes Meeting the Acceptance Criteria ^3,4^ [*n/N* (%)]			Analytes Outside the Criteria at ≥2 Levels ^5^
**LC–MS/MS ^1^**	**0.01 mg/kg**	**0.02 mg/kg**	**0.1 mg/kg**	**Analyte(s)**
Lamb	139/145 (95.9)	136/147 (92.5)	134/150 (89.3)	Bifenazate, cyromazine, dichlorvos, fenhexamid, pyrimethanil-4-hydroxy, pyrimethanil-5-hydroxy, saflufenacil
Duck meat	143/147 (97.3)	143/148 (96.6)	147/150 (98.0)	Cyromazine, pyrimethanil-5-hydroxy, saflufenacil
Quail egg	143/145 (98.6)	146/146 (100.0)	150/150 (100.0)	— ^6^
Mealworm	145/146 (99.3)	144/147 (98.0)	149/150 (99.3)	Dichlorvos
Edible frog	145/147 (98.6)	144/147 (98.0)	148/150 (98.7)	Saflufenacil
**GC–MS/MS ^2^**	**0.01 mg/kg**	**0.02 mg/kg**	**0.1 mg/kg**	**Analyte(s)**
Lamb	73/73 (100.0)	74/74 (100.0)	75/75 (100.0)	—
Duck meat	73/73 (100.0)	75/75 (100.0)	74/75 (98.7)	—
Quail egg	72/72 (100.0)	73/73 (100.0)	75/75 (100.0)	—
Mealworm	75/75 (100.0)	75/75 (100.0)	75/75 (100.0)	—
Edible frog	74/74 (100.0)	74/74 (100.0)	75/75 (100.0)	—

^1^ A total of 150 pesticides were determined by LC–MS/MS in each matrix. ^2^ A total of 75 pesticides were determined by GC–MS/MS in each matrix. ^3^ Analytes with LOQs at or below the fortification level were counted in *N*; those without a reported recovery at that level were not counted in *n*. ^4^ Acceptance criteria: 60–120% recovery with RSD ≤32% at 0.01 mg/kg, and 70–120% recovery with RSD ≤22% at 0.02 and 0.1 mg/kg; *n* = 5 replicates per level. ^5^ Analytes whose mean recovery or RSD fell outside the criteria at two or more levels. ^6^ The symbol “—” indicates that no analytes fell outside the acceptance criteria at two or more fortification levels.

**Table 4:** Pesticide residues detected in minor livestock and animal-derived foods collected in the Republic of Korea.

Analyte	Commodity	Positive Samples (*n/N*) ^1^	Analytical Platform	LOQ ^2^ (mg/kg)	Residue Concentration (mg/kg)
Bifenthrin	Horse meat	1/10	GC–MS/MS	0.006	0.011
Flubendiamide	Goose meat	1/5	LC–MS/MS	0.001	0.015
Lufenuron	Edible insect	1/4	LC–MS/MS	0.002	0.064
Isoprothiolane	Edible snail	1/1	LC–MS/MS	0.010	0.350

^1^ Positive samples (*n*) are expressed as the number of samples containing a quantifiable residue relative to the number of samples (*N*) examined within the corresponding commodity category. ^2^ LOQs for horse meat and goose meat were assigned using the validated lamb and duck-meat matrices, respectively, and the mealworm LOQ was applied to the edible-insect category. For edible snail, for which no comparable validated matrix was available, an LOQ of 0.01 mg/kg was assigned. These assigned LOQs should not be interpreted as directly validated LOQs for monitoring commodities outside the five validation matrices.

**Table 5:** Chronic dietary exposure and risk estimates for pesticide residues detected in minor livestock and animal-derived foods.

Analyte	Commodity	ADI ^1^ (mg/kg b.w./Day)	GECDE*_i_* ^2^ (mg/kg b.w./Day)		%ADI	
			LB ^3^	UB ^4^	LB (%)	UB (%)
Bifenthrin	Horse meat	0.010	1.69 × 10^−6^	1.08 × 10^−5^	1.69 × 10^−2^	1.08 × 10^−1^
Flubendiamide	Goose meat	0.017	3.24 × 10^−5^	4.11 × 10^−5^	1.90 × 10^−1^	2.42 × 10^−1^
Lufenuron	Edible insect	0.015	1.33 × 10^−11^	1.36 × 10^−7^	8.89 × 10^−8^	9.04 × 10^−4^
Isoprothiolane	Edible snail	0.100	2.92 × 10^−10^	1.36 × 10^−7^	2.92 × 10^−7^	1.36 × 10^−4^

^1^ ADI, acceptable daily intake. ^2^ GECDE, global estimate of chronic dietary exposure; GECDE*_i_* includes the exposure term for commodity *i* and mean-intake contributions from the remaining monitored commodities. ^3^ LB, lower bound. ^4^ UB, upper bound.

**Table 6:** Acute dietary exposure and risk estimates for detected pesticides with established acute reference doses.

Analyte	Commodity	*C_max,i_* (mg/kg)	ARfD (mg/kg b.w.)	GEADE*_i_* (mg/kg b.w./Day)	%ARfD (%)
Bifenthrin	Horse meat	0.011	0.010	1.69 × 10^−5^	0.169
Flubendiamide	Goose meat	0.015	0.200	1.62 × 10^−4^	0.0809

## Data Availability

The original contributions presented in this study are included in the article/Supplementary Material. Further inquiries can be directed to the corresponding authors.

## Supplementary Materials

The following supporting information can be downloaded at: https://www.mdpi.com/article/10.3390/molecules31213991/s1, Table S1: Individual LC–MS/MS validation results for pesticide residues in five representative minor-species matrices; Table S2: Individual GC–MS/MS validation results for pesticide residues in five representative minor-species matrices; Table S3: Commodity-specific intake values, surrogate assignments, and LOQ assignments used in the dietary exposure assessment; Table S4: LC–MS/MS multiple reaction monitoring (MRM) parameters and retention times for the target pesticide analytes; Table S5: GC–MS/MS multiple reaction monitoring (MRM) parameters and retention times for the target pesticide analytes.

## References

[1] OECD (2007). Test No. 505: Residues in Livestock.

[2] Hwang M., Lee S.C., Park J.-H., Choi J., Lee H.-J. (2023). Statistical methods for handling nondetected results in food chemical monitoring data to improve food risk assessments. Food Sci. Nutr..

[3] Medina Pastor P., Carrasco Cabrera L., Di Piazza G., González Ciria C., European Food Safety Authority (2026). The 2024 European Union report on pesticide residues in food. EFSA J..

[4] Kang H.S., Kim M., Kim E.J., Choe W.-J. (2020). Determination of 66 pesticide residues in livestock products using QuEChERS and GC–MS/MS. Food Sci. Biotechnol..

[5] Shin Y., Kim C.J., Baek S., Kim L., Son K.-A., Lee H.-D., Kim D., Kim J.-H., Noh H.H. (2020). Liquid chromatography-tandem mass spectrometry for the simultaneous analysis of 353 pesticides in the edible insect *Tenebrio molitor* larvae (mealworms). Molecules.

[6] Saito-Shida S., Saito M., Tsutsumi T. (2025). Pesticide residue analysis of edible insects: Method development and market survey in Japan. Food Addit. Contam. Part A Chem. Anal. Control Expo. Risk Assess..

[7] Camara M.A., Fuster A., Oliva J. (2020). Determination of pesticide residues in edible snails with QuEChERS coupled to GC-MS/MS. Food Addit. Contam. Part A Chem. Anal. Control Expo. Risk Assess..

[8] Anastassiades M., Lehotay S.J., Štajnbaher D., Schenck F.J. (2003). Fast and easy multiresidue method employing acetonitrile extraction/partitioning and “dispersive solid-phase extraction” for the determination of pesticide residues in produce. J. AOAC Int..

[9] Rejczak T., Tuzimski T. (2015). A review of recent developments and trends in the QuEChERS sample preparation approach. Open Chem..

[10] Lichtmannegger K., Fischer R., Steemann F.X., Unterluggauer H., Masselter S. (2015). Alternative QuEChERS-based modular approach for pesticide residue analysis in food of animal origin. Anal. Bioanal. Chem..

[11] Lee S.-H., Kwak S.-Y., Sarker A., Moon J.-K., Kim J.-E. (2022). Optimization of a multi-residue analytical method during determination of pesticides in meat products by GC-MS/MS. Foods.

[12] Kim B.J., Yang S.-H., Choi H. (2022). Simultaneous determination of pyrethroid insecticides in foods of animal origins using the modified QuEChERS method and gas chromatography-mass spectrometry. Foods.

[13] Golge O., Liman T., Kabak B. (2021). Determination of more than 500 pesticide residues in hen eggs by liquid chromatography-tandem mass spectrometry (LC-MS/MS) and gas chromatography-tandem mass spectrometry (GC/MS/MS). Food Sci. Anim. Resour..

[14] Jia Q., Liao G.-Q., Chen L., Qian Y.-Z., Yan X., Qiu J. (2024). Pesticide residues in animal-derived food: Current state and perspectives. Food Chem..

[15] Ninga E., Lehotay S.J., Sapozhnikova Y., Lightfield A.R., Strahan G.D., Monteiro S.H. (2022). Analysis of pesticides, veterinary drugs, and environmental contaminants in goat and lamb by the QuEChERSER mega-method. Anal. Methods.

[16] Sun Q., Huang Y., Liu J., Li P., Gou Y., Hua Y. (2025). Method development, residue level analysis, and dietary risk assessment of 25 novel pesticide residues in animal-derived foods based on modified QuEChERS–HPLC-MS/MS. Microchem. J..

[17] Noh H.H., Kim C.J., Kim S.-H., Eun H.-R., Shin Y., Jeong W.T. (2025). GC–MS/MS–based multiresidue pesticide analysis in mealworm (*Tenebrio molitor*) larvae: Optimization of standard QuEChERS-based method to minimize matrix effects. Food Chem. X.

[18] World Health Organization, Food and Agriculture Organization of the United Nations (2020). Dietary exposure assessment for chemicals in food. Principles and Methods for the Risk Assessment of Chemicals in Food.

[19] European Food Safety Authority (2010). Management of left-censored data in dietary exposure assessment of chemical substances. EFSA J..

[20] European Parliament and Council (2005). Regulation (EC) No 396/2005 of the European Parliament and of the Council of 23 February 2005 on maximum residue levels of pesticides in or on food and feed of plant and animal origin and amending Council Directive 91/414/EEC. Off. J. Eur. Union.

[21] Tongchai P., Sawarng N., Wongta A., Jaitham U., Sutan K., Kawichai S., Jung C., Chuttong B., Hongsibsong S. (2025). Optimization and validation of a QuEChERS-based method combined with gas chromatography–tandem mass spectrometry for analyzing pesticide residues in edible insect samples. Molecules.

[22] Wang J., Zhang B., Zhu J., Ji J., Liu D., Gao R., Ma Y. (2023). Ferric chloride assisted QuEChERS method for separate detection of bifenazate and bifenazate-diazene in citrus fruits and its field validation. Food Chem..

[23] Hamdache S., Sleiman M., de Sainte-Claire P., Jaber F., Richard C. (2018). Unravelling the reactivity of bifenazate in water and on vegetables: Kinetics and byproducts. Sci. Total Environ..

[24] Alvarez F., Arena M., Auteri D., Borroto J., Brancato A., Carrasco Cabrera L., Castoldi A.F., Chiusolo A., Colagiorgi A., European Food Safety Authority (2021). Updated peer review of the pesticide risk assessment of the active substance bifenazate. EFSA J..

[25] Joint FAO/WHO Meeting on Pesticide Residues (2008). Pyrimethanil. Pesticide Residues in Food 2007, Evaluations Part I—Residues.

[26] Schieber A. (2018). Reactions of quinones—Mechanisms, structures, and prospects for food research. J. Agric. Food Chem..

[27] Lewis K.A., Tzilivakis J., Warner D.J., Green A. (2016). An international database for pesticide risk assessments and management. Hum. Ecol. Risk Assess..

[28] Faustman C., Sun Q., Mancini R., Suman S.P. (2010). Myoglobin and lipid oxidation interactions: Mechanistic bases and control. Meat Sci..

[29] Xia K., Atkins J., Foster C., Armbrust K. (2010). Analysis of cyromazine in poultry feed using the QuEChERS method coupled with LC-MS/MS. J. Agric. Food Chem..

[30] Steinborn A., Alder L., Spitzke M., Dörk D., Anastassiades M. (2017). Development of a QuEChERS-based method for the simultaneous determination of acidic pesticides, their esters, and conjugates following alkaline hydrolysis. J. Agric. Food Chem..

[31] Shin Y., Lee J., Kim J.-H. (2018). A simultaneous multiresidue analysis for 203 pesticides in soybean using florisil solid-phase extraction and gas chromatography–tandem mass spectrometry. Appl. Biol. Chem..

[32] Kim D.-J., Oh E.-B., Moon J.-H., Choi J.-W., Kim T.-H., Lee S.-H., Park J.-Y., Kwon C.-H., Kyung K.-S. (2025). Nationwide monitoring and risk assessment of pesticide residues in fishery products. Toxics.

[33] World Health Organization, Food and Agriculture Organization of the United Nations (2015). Pesticide Residues in Food 2015: Report of the Joint FAO/WHO Meeting on Pesticide Residues.

[34] World Health Organization, Food and Agriculture Organization of the United Nations (2017). Pesticide Residues in Food 2017: Report of the Joint FAO/WHO Meeting on Pesticide Residues.

[35] Codex Alimentarius Commission (2010). Guidelines on Good Laboratory Practice in Pesticide Residue Analysis.

[36] Dong-A University (2025). A Study on the Investigation of Residual Substances in Various Minor Livestock Species.

[37] Boobis A., Cerniglia C., Chicoine A., Fattori V., Lipp M., Reuss R., Verger P., Tritscher A. (2017). Characterizing chronic and acute health risks of residues of veterinary drugs in food: Latest methodological developments by the joint FAO/WHO expert committee on food additives. Crit. Rev. Toxicol..

[38] World Health Organization (2016). Evaluation of Certain Veterinary Drug Residues in Food: Eighty-First Report of the Joint FAO/WHO Expert Committee on Food Additives.

[39] Chung H.-R., Kim M.-S., Song T.-J., Lee J.-K., Lim K.-M., Choi S.-H., Joo H.-J., Ko A.-R., Kwon N.-J. (2018). Estimating the Amounts of Foods, Consumed but Not Calculated in KNHANES, for Dietary Exposure Assessment of Pesticides.

[40] Sardar S.W., Byeon G.D., Choi J.Y., Ham H.J., Ishag A.E.S.A., Hur J.H. (2022). Residual characteristics and safety assessment of the insecticides spiromesifen and chromafenozide in lettuce and perilla. Sci. Rep..

[41] Rural Development Administration (2026). Standards for Registration of Pesticides and Active Ingredients: Annex 4-2, Acceptable Daily Intakes of Pesticides.

[42] World Health Organization, Food and Agriculture Organization of the United Nations (2009). Pesticide Residues in Food 2009: Report of the Joint FAO/WHO Meeting on Pesticide Residues.

[43] World Health Organization, Food and Agriculture Organization of the United Nations (2011). Pesticide Residues in Food 2010: Report of the Joint FAO/WHO Meeting on Pesticide Residues.

