# Supplementary material for: QuEChERS-Based Multiresidue Pesticide Analysis in Minor Livestock Products and Other Animal-Derived Foods: Performance Evaluation in Selected Matrices and a Market Survey in Korea
Source: Molecules. 2026 Nov 5;31(21):3991. doi: 10.3390/molecules31213991 (PMC13764761; doi:10.3390/molecules31213991)
Supplement: Supplementary file 1 [file molecules-31-03991-s001.zip › molecules-4621475-supplementary-final.pdf]

Table S1. Individual LC–MS/MS validation results for pesticide residues in five representative minor-species matrices.

No.	Analyte	Matrix	LOQ (mg/kg)	r^2	Recovery at 0.01 mg/kg (%, RSD %)	Recovery at 0.02 mg/kg (%, RSD %)	Recovery at 0.1 mg/kg (%, RSD %)
1	Abamectin	Lamb	0.004	0.9967	77.8 (26.4)	72.6 (21.4)	94.8 (10.6)
		Duck meat	0.01	0.9971	94.7 (31.4)	94.0 (22.0)	93.4 (2.8)
		Quail egg	0.001	0.9960	82.5 (16.5)	76.6 (14.2)	80.6 (19.7)
		Mealworm	0.004	0.9960	80.4 (30.4)	97.4 (16.3)	82.3 (11.5)
		Edible frog	0.002	0.9853	96.4 (28.5)	74.4 (21.9)	88.7 (8.1)
2	Acephate	Lamb	0.004	0.9957	86.7 (13.2)	88.7 (12.7)	82.5 (7.5)
		Duck meat	0.001	0.9914	88.8 (10.0)	87.8 (6.4)	91.4 (4.3)
		Quail egg	0.001	0.9927	87.2 (14.7)	88.8 (5.0)	87.7 (7.0)
		Mealworm	0.004	0.9952	60.7 (23.5)	75.8 (7.4)	88.7 (9.6)
		Edible frog	0.01	0.9963	75.7 (21.1)	74.5 (11.0)	87.4 (2.9)
3	Acetamiprid	Lamb	0.001	0.9961	107.5 (3.8)	96.5 (17.7)	95.8 (9.2)
		Duck meat	0.001	0.9965	107.6 (3.7)	105.5 (6.6)	99.7 (4.1)
		Quail egg	0.001	0.9915	106.6 (8.8)	99.1 (5.6)	102.5 (2.8)
		Mealworm	0.001	0.9974	102.9 (4.4)	100.6 (6.9)	101.7 (6.0)
		Edible frog	0.001	0.9943	107.7 (8.2)	106.1 (3.2)	94.3 (4.7)
4	Acetamiprid-N-desmethyl (IM-2-1)	Lamb	0.001	0.9926	96.8 (12.3)	102.1 (8.7)	98.7 (4.5)
		Duck meat	0.001	0.9937	90.3 (28.6)	99.2 (12.3)	95.5 (12.2)
		Quail egg	0.002	0.9920	102.6 (9.0)	92.5 (19.2)	96.6 (3.1)
		Mealworm	0.001	0.9975	104.1 (3.6)	100.2 (4.2)	100.5 (5.3)
		Edible frog	0.001	0.9911	116.0 (14.5)	101.1 (11.2)	95.3 (5.3)
5	Afidopyropen	Lamb	0.02	0.9952	- [1]	119.0 (14.0)	93.6 (21.3)
		Duck meat	0.01	0.9972	67.3 (21.4)	91.8 (21.4)	95.5 (14.9)
		Quail egg	0.02	0.9819	-	87.5 (21.5)	103.7 (13.0)
		Mealworm	0.01	0.9997	60.1 (24.2)	67.1 (15.2)	95.7 (20.8)
		Edible frog	0.002	0.9682	62.7 (28.7)	80.9 (21.8)	86.5 (20.1)
6	Alachlor	Lamb	0.001	0.9963	105.2 (12.3)	95.8 (9.2)	94.2 (2.5)
		Duck meat	0.001	0.9931	109.3 (6.4)	101.8 (10.8)	92.7 (5.0)
		Quail egg	0.001	0.9935	96.8 (8.3)	105.7 (4.1)	94.4 (8.4)
		Mealworm	0.001	0.9946	109.2 (8.8)	101.0 (9.7)	95.7 (5.6)
		Edible frog	0.001	0.9973	99.5 (9.4)	99.4 (4.5)	99.3 (2.1)
7	Aldicarb	Lamb	0.002	0.9948	114.2 (34.8)	102.5 (17.7)	119.2 (4.9)
		Duck meat	0.004	0.9986	65.7 (17.9)	105.3 (18.4)	91.3 (8.1)
		Quail egg	0.002	0.9995	89.2 (14.2)	93.6 (19.1)	109.1 (6.0)
		Mealworm	0.01	0.9951	100.6 (27.5)	96.6 (21.1)	89.4 (12.9)
		Edible frog	0.001	0.9908	116.7 (30.9)	104.7 (15.3)	105.8 (7.2)
8	Aldicarb-sulfone	Lamb	0.001	0.9983	102.7 (12.0)	97.9 (11.8)	100.1 (4.1)
		Duck meat	0.001	0.9953	107.7 (7.8)	107.3 (6.0)	98.2 (7.9)
		Quail egg	0.001	0.9968	99.5 (9.1)	101.5 (10.0)	98.4 (7.6)
		Mealworm	0.001	0.9929	99.6 (14.4)	104.1 (7.2)	101.0 (7.4)
		Edible frog	0.001	0.9940	110.4 (13.6)	107.5 (11.8)	99.5 (4.6)

No.	Analyte	Matrix	LOQ (mg/kg)	r^2	Recovery at 0.01 mg/kg (%, RSD %)	Recovery at 0.02 mg/kg (%, RSD %)	Recovery at 0.1 mg/kg (%, RSD %)
9	Aldicarb-sulfoxide	Lamb	0.001	0.9953	95.5 (5.9)	96.1 (10.0)	93.1 (4.4)
		Duck meat	0.001	0.9954	98.5 (6.6)	97.0 (6.9)	88.2 (7.5)
		Quail egg	0.001	0.9951	93.0 (7.5)	92.3 (5.9)	93.9 (4.8)
		Mealworm	0.001	0.9969	93.3 (12.0)	86.4 (5.2)	97.4 (2.4)
		Edible frog	0.002	0.9951	98.9 (13.2)	95.2 (8.1)	98.4 (4.5)
10	Ametoctradin	Lamb	0.001	0.9982	81.9 (7.2)	87.9 (3.6)	86.9 (5.9)
		Duck meat	0.001	0.9988	89.7 (6.8)	78.1 (9.5)	84.2 (7.1)
		Quail egg	0.001	0.9933	93.3 (5.2)	88.9 (3.5)	91.6 (2.0)
		Mealworm	0.001	0.9960	82.9 (8.0)	80.2 (5.4)	82.8 (3.9)
		Edible frog	0.001	0.9968	96.2 (6.4)	91.1 (3.9)	86.0 (2.5)
11	Azinphos-methyl	Lamb	0.002	0.9902	93.0 (16.1)	90.1 (12.9)	90.2 (12.1)
		Duck meat	0.002	0.9994	72.2 (30.9)	95.5 (18.9)	95.1 (15.5)
		Quail egg	0.004	0.9985	87.6 (30.9)	90.1 (16.1)	94.6 (15.4)
		Mealworm	0.004	0.9974	101.7 (14.9)	115.1 (13.8)	103.6 (8.2)
		Edible frog	0.002	0.9919	101.0 (25.2)	85.8 (19.0)	100.8 (11.9)
12	Azoxystrobin	Lamb	0.001	0.9969	93.7 (7.6)	90.6 (9.1)	87.0 (8.4)
		Duck meat	0.001	0.9923	95.4 (18.3)	98.9 (3.8)	104.1 (4.2)
		Quail egg	0.004	0.9987	101.6 (6.1)	100.1 (3.9)	104.2 (4.3)
		Mealworm	0.001	0.9976	95.3 (13.2)	104.7 (4.2)	106.0 (5.3)
		Edible frog	0.001	0.9977	103.4 (12.7)	86.4 (21.7)	93.0 (21.1)
13	Bendiocarb	Lamb	0.001	0.9986	97.5 (3.4)	101.3 (3.5)	99.4 (2.9)
		Duck meat	0.001	0.9987	99.8 (7.6)	99.2 (5.4)	93.7 (1.9)
		Quail egg	0.001	0.9965	91.7 (4.3)	91.5 (9.2)	93.3 (4.6)
		Mealworm	0.001	0.9983	105.2 (5.8)	105.0 (5.6)	102.5 (3.9)
		Edible frog	0.001	0.9949	112.0 (13.1)	101.8 (6.6)	100.8 (3.8)
14	Bentazone	Lamb	0.001	0.9973	73.0 (7.5)	72.7 (14.0)	72.0 (7.0)
		Duck meat	0.004	0.9919	60.6 (10.9)	72.5 (4.3)	76.9 (6.4)
		Quail egg	0.002	0.9963	81.3 (8.9)	77.9 (6.1)	74.8 (4.9)
		Mealworm	0.001	0.9926	86.8 (14.0)	88.6 (3.5)	80.0 (8.2)
		Edible frog	0.002	0.9901	76.7 (23.6)	82.0 (5.9)	73.6 (2.3)
15	Benzovindiflupyr	Lamb	0.001	0.9948	111.2 (12.4)	96.9 (8.7)	95.0 (4.5)
		Duck meat	0.001	0.9963	88.7 (18.7)	103.7 (7.3)	99.0 (5.3)
		Quail egg	0.001	0.9970	96.1 (11.8)	98.7 (10.8)	95.0 (14.4)
		Mealworm	0.001	0.9928	95.9 (12.4)	107.4 (6.4)	102.1 (2.8)
		Edible frog	0.001	0.9953	97.3 (10.4)	88.2 (12.4)	90.7 (18.3)
16	Bifenazate	Lamb	0.001	0.9976	19.8 (20.4)	18.3 (20.9)	17.0 (9.7)
		Duck meat	0.001	0.9925	82.9 (11.1)	73.8 (12.0)	72.5 (10.4)
		Quail egg	0.001	0.9966	86.9 (3.3)	93.9 (5.8)	100.6 (4.2)
		Mealworm	0.001	0.9918	110.3 (8.1)	93.2 (14.6)	96.6 (3.2)
		Edible frog	0.001	0.9964	111.4 (7.6)	94.7 (4.4)	98.9 (3.1)
17	Bifenazate-diazene	Lamb	0.001	0.9972	119.3 (8.6)	113.3 (10.1)	114.8 (5.5)
		Duck meat	0.001	0.9978	97.4 (7.1)	101.5 (7.0)	102.4 (2.6)
		Quail egg	0.001	0.9987	96.7 (6.7)	93.8 (5.8)	87.8 (4.3)
		Mealworm	0.001	0.9978	92.9 (11.0)	88.7 (6.6)	93.9 (5.9)
		Edible frog	0.001	0.9975	97.3 (6.9)	88.4 (5.5)	85.1 (10.0)

No.	Analyte	Matrix	LOQ (mg/kg)	r^2	Recovery at 0.01 mg/kg (%, RSD %)	Recovery at 0.02 mg/kg (%, RSD %)	Recovery at 0.1 mg/kg (%, RSD %)
18	Bioresmethrin	Lamb	0.001	0.9996	80.0 (16.6)	94.9 (10.7)	94.7 (17.7)
		Duck meat	0.001	0.9942	87.1 (6.7)	72.3 (16.6)	72.6 (21.5)
		Quail egg	0.001	0.9954	85.0 (7.7)	82.6 (7.9)	82.0 (6.9)
		Mealworm	0.004	0.9922	74.3 (23.0)	78.5 (21.1)	78.7 (10.1)
		Edible frog	0.001	0.9886	108.6 (16.1)	84.9 (19.7)	91.9 (10.3)
19	Bixafen	Lamb	0.001	0.9958	111.7 (21.5)	101.3 (7.9)	97.2 (6.6)
		Duck meat	0.001	0.9982	97.5 (11.6)	87.5 (17.6)	99.7 (4.1)
		Quail egg	0.001	0.9910	92.6 (12.7)	95.0 (7.8)	94.6 (7.9)
		Mealworm	0.001	0.9927	110.2 (11.9)	103.5 (11.9)	105.1 (5.6)
		Edible frog	0.001	0.9926	114.2 (14.2)	101.8 (2.4)	93.2 (7.0)
20	Boscalid	Lamb	0.001	0.9908	86.9 (15.4)	92.6 (14.7)	99.4 (4.7)
		Duck meat	0.001	0.9978	99.5 (10.2)	97.8 (15.1)	94.4 (3.5)
		Quail egg	0.001	0.9956	76.5 (17.7)	83.8 (5.6)	93.4 (7.4)
		Mealworm	0.001	0.9957	98.4 (19.5)	92.9 (13.9)	96.5 (7.0)
		Edible frog	0.001	0.9930	98.8 (1.6)	98.2 (11.3)	93.0 (13.7)
21	Carbaryl	Lamb	0.001	0.9975	99.9 (4.7)	93.8 (7.0)	96.2 (5.7)
		Duck meat	0.001	0.9985	99.9 (5.0)	93.8 (3.8)	97.1 (3.5)
		Quail egg	0.001	0.9987	94.2 (5.1)	98.8 (5.1)	95.8 (4.2)
		Mealworm	0.001	0.9983	105.1 (5.9)	102.3 (2.0)	101.4 (2.5)
		Edible frog	0.001	0.9969	109.6 (5.7)	101.8 (6.3)	100.7 (3.8)
22	Carbendazim	Lamb	0.001	0.9991	85.9 (7.6)	80.4 (14.5)	86.7 (4.4)
		Duck meat	0.001	0.9987	88.0 (4.2)	87.6 (6.1)	82.0 (7.6)
		Quail egg	0.001	0.9978	89.0 (5.7)	88.9 (5.7)	90.2 (4.4)
		Mealworm	0.001	0.9976	84.0 (6.1)	80.5 (3.8)	77.9 (4.7)
		Edible frog	0.001	0.9970	93.9 (6.1)	90.7 (6.1)	89.7 (3.8)
23	Carbofuran	Lamb	0.001	0.9980	97.1 (8.7)	97.8 (3.0)	98.7 (3.6)
		Duck meat	0.001	0.9981	101.3 (3.5)	101.2 (2.8)	99.3 (1.2)
		Quail egg	0.001	0.9985	95.7 (4.2)	96.7 (7.7)	97.6 (2.1)
		Mealworm	0.001	0.9990	97.5 (6.4)	97.3 (2.6)	100.5 (3.2)
		Edible frog	0.001	0.9988	109.4 (9.8)	104.4 (4.9)	100.7 (3.6)
24	Carbofuran-3-hydroxy	Lamb	0.001	0.9940	103.4 (6.6)	98.5 (10.9)	94.7 (9.5)
		Duck meat	0.001	0.9938	96.2 (21.2)	97.0 (6.6)	91.8 (9.1)
		Quail egg	0.001	0.9917	95.3 (18.4)	92.0 (14.1)	101.1 (4.0)
		Mealworm	0.001	0.9908	112.2 (9.0)	97.1 (6.2)	102.3 (6.3)
		Edible frog	0.001	0.9927	108.0 (17.1)	99.3 (6.4)	95.2 (4.1)
25	Carpropamide	Lamb	0.001	0.9992	96.2 (5.2)	96.4 (3.3)	94.5 (3.1)
		Duck meat	0.001	0.9989	96.6 (7.7)	97.8 (5.4)	92.6 (2.9)
		Quail egg	0.001	0.9916	97.4 (9.2)	104.9 (8.3)	99.2 (4.0)
		Mealworm	0.001	0.9992	95.5 (4.1)	98.2 (2.2)	98.3 (2.6)
		Edible frog	0.001	0.9911	103.3 (6.5)	85.5 (16.4)	93.9 (13.3)
26	Chlorantraniliprole	Lamb	0.002	0.9979	85.7 (25.1)	88.8 (8.5)	99.6 (5.8)
		Duck meat	0.001	0.9926	99.3 (12.0)	92.8 (6.0)	98.3 (3.3)
		Quail egg	0.004	0.9968	89.1 (25.2)	94.4 (12.6)	104.7 (11.3)
		Mealworm	0.001	0.9967	105.1 (21.5)	105.7 (7.5)	106.0 (8.4)
		Edible frog	0.001	0.9940	105.3 (15.6)	107.4 (7.1)	95.8 (4.2)

No.	Analyte	Matrix	LOQ (mg/kg)	r^2	Recovery at 0.01 mg/kg (%, RSD %)	Recovery at 0.02 mg/kg (%, RSD %)	Recovery at 0.1 mg/kg (%, RSD %)
27	Chromafenozide	Lamb	0.001	0.9999	117.4 (12.5)	119.4 (16.6)	119.1 (2.7)
		Duck meat	0.001	0.9960	101.2 (13.9)	99.0 (5.2)	95.9 (5.6)
		Quail egg	0.001	0.9984	102.6 (6.0)	100.1 (1.7)	98.6 (5.0)
		Mealworm	0.002	0.9984	99.9 (9.6)	97.8 (18.6)	102.3 (3.7)
		Edible frog	0.001	0.9923	105.6 (14.6)	101.9 (5.9)	102.9 (3.7)
28	Clofentezine	Lamb	0.001	0.9905	101.4 (5.2)	97.6 (3.1)	94.1 (5.5)
		Duck meat	0.002	0.9945	91.2 (21.5)	105.1 (6.1)	100.9 (3.4)
		Quail egg	0.001	0.9919	97.9 (24.3)	82.8 (19.1)	83.7 (18.8)
		Mealworm	0.001	0.9954	83.7 (16.0)	82.2 (15.1)	84.0 (13.8)
		Edible frog	0.001	0.9972	96.6 (20.5)	94.0 (18.5)	92.3 (12.2)
29	Clothianidin	Lamb	0.002	0.9960	100.1 (14.4)	102.2 (12.0)	100.5 (9.7)
		Duck meat	0.001	0.9976	87.3 (15.9)	94.9 (3.8)	93.8 (4.3)
		Quail egg	0.001	0.9936	101.0 (8.0)	97.7 (8.2)	96.4 (1.7)
		Mealworm	0.001	0.9968	91.1 (12.6)	98.3 (7.6)	100.3 (2.7)
		Edible frog	0.001	0.9928	106.1 (21.2)	97.2 (7.3)	91.4 (3.2)
30	Coumaphos	Lamb	0.001	0.9979	95.4 (8.7)	94.5 (8.3)	91.6 (6.2)
		Duck meat	0.001	0.9965	96.2 (5.7)	94.7 (9.1)	93.1 (6.3)
		Quail egg	0.001	0.9978	98.3 (5.2)	94.8 (8.2)	96.4 (7.1)
		Mealworm	0.001	0.9974	90.4 (12.6)	88.0 (7.6)	102.9 (4.5)
		Edible frog	0.001	0.9963	99.5 (28.8)	90.3 (19.8)	90.6 (10.3)
31	Coumaphos-oxon	Lamb	0.001	0.9978	99.2 (10.2)	95.5 (9.0)	102.9 (5.0)
		Duck meat	0.001	0.9983	94.5 (8.2)	89.6 (9.1)	94.4 (1.9)
		Quail egg	0.001	0.9974	90.0 (9.8)	94.1 (12.8)	99.4 (3.6)
		Mealworm	0.001	0.9983	97.4 (8.8)	98.9 (8.2)	102.8 (4.1)
		Edible frog	0.001	0.9972	107.5 (10.4)	98.0 (5.2)	92.7 (9.0)
32	Cyanazine	Lamb	0.001	0.9983	93.5 (4.5)	90.6 (8.0)	96.5 (3.9)
		Duck meat	0.001	0.9993	91.6 (5.9)	95.3 (4.8)	92.0 (1.1)
		Quail egg	0.001	0.9985	96.4 (4.4)	93.2 (6.6)	97.5 (2.3)
		Mealworm	0.001	0.9985	95.6 (3.6)	97.2 (3.5)	99.0 (8.8)
		Edible frog	0.001	0.9986	112.5 (10.5)	99.6 (5.7)	95.5 (3.1)
33	Cyantraniliprole	Lamb	0.001	0.9981	101.9 (7.9)	101.9 (4.6)	98.5 (2.2)
		Duck meat	0.001	0.9983	100.2 (4.3)	99.5 (4.4)	96.8 (4.7)
		Quail egg	0.001	0.9977	102.1 (3.8)	105.9 (4.4)	96.6 (9.0)
		Mealworm	0.001	0.9971	105.3 (4.2)	104.5 (3.0)	106.6 (3.1)
		Edible frog	0.001	0.9953	110.5 (7.4)	103.5 (5.5)	96.0 (2.2)
34	Cyclaniliprole	Lamb	0.001	0.9941	108.2 (8.4)	99.8 (15.3)	98.6 (9.1)
		Duck meat	0.001	0.9944	91.6 (20.7)	85.5 (8.8)	82.9 (4.9)
		Quail egg	0.002	0.9928	93.9 (12.0)	94.4 (21.9)	95.7 (9.2)
		Mealworm	0.01	0.9987	119.7 (9.2)	104.6 (8.2)	96.4 (7.1)
		Edible frog	0.001	0.9923	78.2 (15.7)	93.6 (17.0)	100.7 (8.0)
35	Cyproconazole	Lamb	0.001	0.9958	77.8 (15.2)	89.1 (9.3)	93.0 (5.8)
		Duck meat	0.001	0.9929	98.2 (14.5)	90.9 (10.1)	95.3 (4.5)
		Quail egg	0.001	0.9970	98.0 (14.7)	96.1 (3.8)	93.9 (3.6)
		Mealworm	0.001	0.9945	95.5 (9.9)	93.7 (15.0)	87.5 (7.4)
		Edible frog	0.001	0.9961	103.4 (9.5)	101.5 (7.8)	97.8 (2.0)

No.	Analyte	Matrix	LOQ (mg/kg)	r^2	Recovery at 0.01 mg/kg (%, RSD %)	Recovery at 0.02 mg/kg (%, RSD %)	Recovery at 0.1 mg/kg (%, RSD %)
36	Cyromazine	Lamb	0.002	0.9916	68.0 (31.1)	68.1 (9.0)	45.7 (15.2)
		Duck meat	0.002	0.9912	47.6 (22.7)	41.7 (9.6)	39.4 (18.5)
		Quail egg	0.01	0.9978	83.7 (29.7)	70.8 (18.2)	72.5 (10.5)
		Mealworm	0.04	0.9990	-	-	49.5 (9.7)
		Edible frog	0.002	0.9912	74.2 (18.8)	92.2 (36.2)	75.0 (11.0)
37	Diazinon	Lamb	0.001	0.9991	89.0 (4.6)	90.2 (5.7)	93.6 (4.0)
		Duck meat	0.001	0.9980	95.1 (7.4)	90.7 (1.8)	89.7 (2.5)
		Quail egg	0.001	0.9948	87.7 (11.7)	97.4 (9.6)	102.1 (2.6)
		Mealworm	0.001	0.9945	88.3 (13.5)	98.8 (2.9)	99.3 (2.8)
		Edible frog	0.001	0.9991	98.3 (11.9)	94.6 (3.1)	96.7 (5.2)
38	Dichlorvos	Lamb	0.004	0.9817	24.7 (34.7)	40.6 (24.6)	61.5 (6.5)
		Duck meat	0.01	0.9949	-	-	87.3 (6.5)
		Quail egg	0.04	0.9942	-	-	104.8 (8.5)
		Mealworm	0.01	0.9883	38.1 (29.5)	64.3 (12.5)	93.2 (5.4)
		Edible frog	0.01	0.9561	-	-	102.8 (9.8)
39	Diflubenzuron	Lamb	0.002	0.9913	99.7 (27.0)	91.1 (24.0)	89.7 (9.9)
		Duck meat	0.002	0.9900	101.2 (26.7)	96.3 (21.8)	97.6 (8.9)
		Quail egg	0.004	0.9957	65.1 (18.6)	81.7 (18.3)	89.2 (11.8)
		Mealworm	0.002	0.9934	97.7 (27.1)	88.9 (18.6)	99.9 (14.5)
		Edible frog	0.001	0.9933	92.5 (23.4)	106.2 (21.2)	105.5 (7.5)
40	Dimethoate	Lamb	0.002	0.9986	104.4 (3.0)	94.6 (6.7)	84.3 (24.4)
		Duck meat	0.001	0.9991	94.2 (19.3)	102.7 (2.4)	96.5 (4.5)
		Quail egg	0.001	0.9980	97.4 (4.9)	97.5 (4.2)	97.7 (1.4)
		Mealworm	0.001	0.9933	97.7 (10.9)	102.9 (8.0)	103.0 (8.9)
		Edible frog	0.001	0.9954	104.4 (11.4)	101.5 (2.1)	97.8 (2.2)
41	Dimethomorph-E	Lamb	0.001	0.9960	90.0 (9.4)	84.3 (17.7)	96.1 (5.9)
		Duck meat	0.001	0.9977	99.6 (3.2)	88.9 (12.7)	89.0 (6.3)
		Quail egg	0.001	0.9914	90.2 (7.2)	95.1 (16.1)	107.5 (2.7)
		Mealworm	0.001	0.9924	107.6 (5.6)	106.7 (14.6)	98.1 (11.2)
		Edible frog	0.001	0.9959	106.3 (18.8)	104.4 (4.4)	98.4 (2.2)
42	Dimethomorph-Z	Lamb	0.001	0.9947	90.1 (15.6)	99.1 (5.8)	96.8 (11.0)
		Duck meat	0.001	0.9986	96.7 (4.2)	98.6 (2.9)	93.3 (3.6)
		Quail egg	0.001	0.9946	97.5 (5.4)	98.5 (6.4)	95.6 (2.5)
		Mealworm	0.001	0.9951	102.1 (3.9)	99.4 (2.1)	101.0 (2.2)
		Edible frog	0.001	0.9962	108.6 (8.8)	98.0 (5.7)	98.7 (3.8)
43	Dinotefuran	Lamb	0.001	0.9952	100.3 (11.7)	90.3 (8.7)	98.1 (4.7)
		Duck meat	0.002	0.9961	103.8 (8.6)	107.9 (12.0)	95.1 (5.2)
		Quail egg	0.001	0.9905	88.2 (21.7)	93.7 (14.8)	97.3 (2.8)
		Mealworm	0.001	0.9901	92.4 (14.0)	98.7 (8.2)	98.5 (6.2)
		Edible frog	0.002	0.9940	118.7 (13.3)	104.6 (19.8)	96.3 (5.7)
44	Disulfoton	Lamb	0.004	0.9830	88.4 (21.1)	105.9 (16.7)	88.8 (27.7)
		Duck meat	0.004	0.9948	80.9 (28.4)	85.1 (11.9)	84.4 (12.7)
		Quail egg	0.002	0.9998	79.7 (25.0)	76.9 (20.1)	91.7 (6.0)
		Mealworm	0.01	0.9964	101.6 (27.8)	78.7 (16.0)	91.1 (17.5)
		Edible frog	0.004	0.9901	106.0 (30.3)	98.9 (21.8)	99.8 (11.4)

No.	Analyte	Matrix	LOQ (mg/kg)	r^2	Recovery at 0.01 mg/kg (%, RSD %)	Recovery at 0.02 mg/kg (%, RSD %)	Recovery at 0.1 mg/kg (%, RSD %)
45	Disulfoton-sulfone	Lamb	0.001	0.9985	97.3 (8.5)	97.0 (4.5)	96.7 (3.7)
		Duck meat	0.001	0.9993	108.4 (4.8)	97.3 (5.0)	98.3 (4.2)
		Quail egg	0.001	0.9983	98.8 (5.9)	97.1 (4.0)	100.6 (4.2)
		Mealworm	0.001	0.9966	111.8 (5.1)	102.4 (7.6)	105.4 (7.9)
		Edible frog	0.001	0.9984	110.6 (11.6)	102.3 (5.3)	100.1 (4.6)
46	Disulfoton-sulfoxide	Lamb	0.001	0.9973	97.3 (4.5)	101.6 (5.6)	98.4 (5.0)
		Duck meat	0.001	0.9973	95.6 (3.4)	101.6 (3.3)	96.7 (7.1)
		Quail egg	0.001	0.9982	95.0 (4.2)	95.3 (4.2)	97.0 (3.4)
		Mealworm	0.001	0.9977	104.4 (0.0)	98.1 (4.5)	100.8 (3.4)
		Edible frog	0.001	0.9961	106.2 (16.6)	100.9 (2.3)	101.8 (1.4)
47	Demeton-S	Lamb	0.001	0.9950	92.0 (20.9)	89.2 (9.1)	96.8 (5.8)
		Duck meat	0.001	0.9960	102.4 (8.7)	99.0 (14.1)	101.5 (2.1)
		Quail egg	0.001	0.9963	98.0 (9.0)	99.3 (13.0)	95.8 (6.0)
		Mealworm	0.001	0.9966	90.3 (18.2)	94.0 (13.0)	87.5 (6.8)
		Edible frog	0.001	0.9930	110.8 (19.5)	100.4 (3.9)	100.9 (6.1)
48	Demeton-S-sulfone	Lamb	0.002	0.9970	92.3 (21.0)	105.3 (4.9)	100.7 (4.7)
		Duck meat	0.001	0.9910	100.5 (4.9)	107.1 (6.2)	98.3 (16.7)
		Quail egg	0.001	0.9901	99.5 (8.5)	105.5 (7.9)	104.0 (4.8)
		Mealworm	0.001	0.9946	104.4 (10.8)	103.4 (4.7)	108.4 (5.3)
		Edible frog	0.001	0.9913	112.4 (15.6)	105.2 (5.6)	103.6 (2.3)
49	Demeton-S-sulfoxide	Lamb	0.001	0.9901	94.4 (30.8)	98.8 (7.6)	91.2 (24.7)
		Duck meat	0.001	0.9974	95.7 (11.4)	98.0 (10.0)	87.6 (19.1)
		Quail egg	0.001	0.9990	91.6 (8.8)	93.3 (5.3)	94.8 (7.1)
		Mealworm	0.001	0.9982	98.6 (3.6)	104.1 (2.6)	102.1 (2.3)
		Edible frog	0.001	0.9977	104.7 (10.5)	101.3 (3.0)	97.7 (1.7)
50	Edifenphos	Lamb	0.001	0.9985	97.4 (7.2)	91.2 (6.7)	98.0 (4.2)
		Duck meat	0.001	0.9986	104.4 (5.9)	93.2 (3.4)	93.0 (3.4)
		Quail egg	0.001	0.9985	92.1 (9.0)	84.5 (13.1)	89.4 (12.4)
		Mealworm	0.001	0.9985	103.6 (9.4)	101.5 (6.0)	93.2 (4.6)
		Edible frog	0.001	0.9984	105.0 (3.1)	92.6 (10.9)	95.0 (4.0)
51	Epoxiconazole	Lamb	0.001	0.9990	97.8 (8.9)	93.2 (3.5)	94.8 (2.5)
		Duck meat	0.001	0.9971	94.9 (3.2)	97.7 (4.0)	94.4 (2.3)
		Quail egg	0.001	0.9976	93.1 (3.8)	100.3 (4.1)	98.8 (1.3)
		Mealworm	0.001	0.9993	99.2 (2.9)	97.2 (2.9)	94.9 (1.8)
		Edible frog	0.001	0.9963	108.3 (7.6)	99.9 (1.8)	98.4 (3.5)
52	Ethiofencarb	Lamb	0.001	0.9941	91.4 (4.4)	95.4 (3.2)	101.2 (5.4)
		Duck meat	0.001	0.9957	107.2 (8.0)	102.4 (6.9)	100.6 (4.8)
		Quail egg	0.001	0.9942	94.1 (3.5)	101.4 (4.3)	94.7 (5.8)
		Mealworm	0.001	0.9942	93.3 (8.7)	102.0 (4.3)	103.8 (7.5)
		Edible frog	0.001	0.9928	116.6 (7.1)	101.6 (6.7)	99.4 (3.8)
53	Ethiprole	Lamb	0.001	0.9917	88.3 (26.3)	87.8 (16.2)	93.7 (11.2)
		Duck meat	0.002	0.9924	93.2 (18.0)	95.0 (19.5)	95.0 (5.3)
		Quail egg	0.002	0.9923	97.7 (21.8)	89.5 (12.2)	98.4 (7.7)
		Mealworm	0.001	0.9911	107.0 (12.3)	101.5 (14.5)	103.0 (12.2)
		Edible frog	0.002	0.9954	101.9 (21.7)	104.6 (14.6)	97.4 (12.0)

No.	Analyte	Matrix	LOQ (mg/kg)	r^2	Recovery at 0.01 mg/kg (%, RSD %)	Recovery at 0.02 mg/kg (%, RSD %)	Recovery at 0.1 mg/kg (%, RSD %)
54	Etrimfos	Lamb	0.001	0.9981	92.7 (6.5)	89.2 (2.2)	90.5 (4.7)
		Duck meat	0.001	0.9973	92.6 (6.9)	90.6 (6.0)	91.9 (4.5)
		Quail egg	0.001	0.9969	85.5 (18.0)	92.1 (20.0)	96.3 (11.2)
		Mealworm	0.001	0.9983	89.4 (7.7)	91.0 (5.3)	95.3 (6.3)
		Edible frog	0.001	0.9917	104.3 (14.4)	100.2 (4.2)	95.0 (13.7)
55	Famoxadone	Lamb	0.004	0.9946	66.1 (28.0)	79.4 (18.7)	97.4 (19.7)
		Duck meat	0.01	0.9801	81.2 (61.3)	98.2 (21.0)	104.0 (8.2)
		Quail egg	0.01	0.9917	85.8 (57.1)	79.6 (17.1)	96.3 (10.1)
		Mealworm	0.01	0.9904	119.5 (10.4)	113.5 (17.7)	112.8 (8.2)
		Edible frog	0.01	0.9943	111.4 (21.9)	92.9 (18.1)	97.0 (11.8)
56	Fenamidone	Lamb	0.001	0.9967	98.7 (2.1)	90.1 (11.6)	94.2 (10.9)
		Duck meat	0.001	0.9963	104.3 (6.7)	94.6 (3.0)	90.1 (10.4)
		Quail egg	0.001	0.9984	96.2 (8.6)	104.4 (5.3)	99.0 (2.7)
		Mealworm	0.001	0.9967	109.8 (6.8)	105.0 (7.1)	97.5 (9.5)
		Edible frog	0.001	0.9979	108.3 (13.1)	103.0 (3.2)	95.7 (3.8)
57	Fenarimol	Lamb	0.001	0.9990	78.7 (30.9)	70.0 (24.1)	98.2 (11.2)
		Duck meat	0.004	0.9953	108.9 (38.0)	106.0 (14.6)	102.8 (7.2)
		Quail egg	0.004	0.9962	80.9 (27.3)	74.9 (21.1)	87.2 (8.8)
		Mealworm	0.01	0.9902	119.9 (22.5)	94.8 (19.4)	85.6 (14.3)
		Edible frog	0.004	0.9900	103.3 (24.8)	109.8 (15.6)	107.2 (9.7)
58	Fenbuconazole	Lamb	0.001	0.9986	97.3 (4.3)	96.9 (5.0)	96.0 (2.4)
		Duck meat	0.001	0.9976	94.1 (8.5)	96.7 (6.4)	93.2 (6.1)
		Quail egg	0.001	0.9965	104.6 (6.0)	101.7 (7.2)	97.8 (6.2)
		Mealworm	0.001	0.9987	97.5 (6.5)	93.1 (2.4)	96.8 (3.8)
		Edible frog	0.001	0.9979	108.2 (8.9)	99.2 (6.3)	95.8 (3.7)
59	Fenhexamid	Lamb	0.001	0.9989	60.7 (26.7)	67.8 (18.5)	55.4 (15.1)
		Duck meat	0.001	0.9905	85.8 (14.4)	77.4 (7.4)	77.3 (3.3)
		Quail egg	0.001	0.9969	76.1 (11.0)	81.6 (7.4)	71.8 (16.2)
		Mealworm	0.001	0.9925	70.0 (28.9)	71.3 (13.3)	73.8 (20.4)
		Edible frog	0.001	0.9962	76.7 (31.4)	84.7 (8.3)	80.4 (5.5)
60	Fenobucarb	Lamb	0.001	0.9953	99.6 (4.2)	93.4 (2.4)	97.2 (3.1)
		Duck meat	0.004	0.9963	97.1 (16.4)	102.9 (2.7)	94.4 (3.8)
		Quail egg	0.001	0.9962	96.8 (4.6)	97.9 (2.9)	99.9 (1.7)
		Mealworm	0.002	0.9964	103.6 (2.7)	91.2 (16.0)	101.3 (8.4)
		Edible frog	0.001	0.9978	102.1 (16.8)	97.0 (9.1)	96.7 (5.0)
61	Fenpyroximate	Lamb	0.001	0.9909	78.8 (9.8)	81.5 (8.4)	83.8 (7.5)
		Duck meat	0.001	0.9975	87.8 (7.4)	84.2 (7.9)	84.1 (7.0)
		Quail egg	0.001	0.9970	85.7 (9.4)	82.5 (9.3)	92.1 (8.5)
		Mealworm	0.001	0.9954	74.1 (7.2)	70.3 (6.8)	86.7 (5.9)
		Edible frog	0.001	0.9978	96.5 (13.7)	89.5 (7.2)	82.1 (4.5)
62	Fensulfothion	Lamb	0.001	0.9955	94.8 (11.3)	94.0 (11.3)	98.5 (5.0)
		Duck meat	0.001	0.9958	105.4 (7.3)	100.4 (8.9)	97.6 (4.0)
		Quail egg	0.001	0.9958	97.5 (15.4)	101.5 (11.3)	95.6 (5.7)
		Mealworm	0.001	0.9960	97.2 (12.9)	100.4 (16.0)	106.6 (7.7)
		Edible frog	0.001	0.9971	93.4 (16.7)	105.9 (6.0)	98.5 (6.8)

No.	Analyte	Matrix	LOQ (mg/kg)	r^2	Recovery at 0.01 mg/kg (%, RSD %)	Recovery at 0.02 mg/kg (%, RSD %)	Recovery at 0.1 mg/kg (%, RSD %)
63	Ferimzone-E	Lamb	0.001	0.9988	91.6 (4.0)	89.2 (3.4)	89.6 (3.3)
		Duck meat	0.001	0.9977	95.5 (2.2)	95.7 (6.3)	91.4 (5.1)
		Quail egg	0.001	0.9980	97.2 (7.5)	97.5 (1.9)	94.9 (2.3)
		Mealworm	0.001	0.9992	86.0 (4.2)	85.6 (4.7)	89.5 (4.7)
		Edible frog	0.001	0.9983	105.6 (6.7)	101.5 (4.2)	97.6 (4.4)
64	Ferimzone-Z	Lamb	0.001	0.9981	88.2 (6.2)	91.2 (6.0)	92.2 (3.9)
		Duck meat	0.001	0.9952	100.3 (7.3)	95.6 (4.5)	97.0 (5.2)
		Quail egg	0.001	0.9970	91.9 (11.3)	93.7 (9.7)	89.2 (3.8)
		Mealworm	0.001	0.9993	93.6 (7.1)	90.5 (2.7)	91.6 (3.6)
		Edible frog	0.001	0.9963	107.3 (8.5)	102.0 (4.3)	98.2 (4.8)
65	Fipronil	Lamb	0.001	0.9975	95.3 (13.6)	104.0 (5.4)	97.3 (5.6)
		Duck meat	0.001	0.9922	102.6 (8.0)	105.3 (11.5)	91.7 (17.6)
		Quail egg	0.001	0.9902	107.9 (6.2)	108.7 (11.2)	98.1 (5.3)
		Mealworm	0.001	0.9976	109.3 (13.6)	104.3 (9.0)	98.5 (16.8)
		Edible frog	0.001	0.9962	115.5 (14.9)	105.7 (6.2)	97.0 (3.6)
66	Fipronil-sulfone	Lamb	0.001	0.9950	98.6 (9.5)	90.1 (11.3)	99.0 (4.0)
		Duck meat	0.001	0.9963	97.7 (15.6)	95.3 (10.5)	95.4 (4.4)
		Quail egg	0.001	0.9876	84.0 (30.4)	109.5 (21.7)	105.2 (13.4)
		Mealworm	0.001	0.9984	92.4 (11.6)	98.9 (14.0)	99.6 (9.3)
		Edible frog	0.001	0.9988	100.3 (8.8)	99.5 (5.6)	94.7 (7.2)
67	Flubendiamide	Lamb	0.001	0.9961	97.7 (9.8)	95.0 (11.4)	79.3 (33.0)
		Duck meat	0.001	0.9951	99.9 (24.8)	104.0 (7.4)	97.4 (3.4)
		Quail egg	0.001	0.9965	111.3 (5.2)	100.6 (14.3)	100.0 (4.5)
		Mealworm	0.002	0.9923	106.2 (5.9)	99.3 (13.8)	107.5 (3.0)
		Edible frog	0.001	0.9919	109.2 (10.9)	102.5 (2.9)	99.7 (2.2)
68	Fludioxonil	Lamb	0.002	0.9983	84.8 (30.4)	98.3 (17.1)	104.7 (13.4)
		Duck meat	0.001	0.9933	74.2 (30.9)	94.9 (19.5)	84.6 (15.7)
		Quail egg	0.002	0.9950	97.7 (31.0)	92.6 (7.7)	87.2 (11.4)
		Mealworm	0.001	0.9966	77.8 (30.4)	88.6 (22.0)	102.3 (8.4)
		Edible frog	0.002	0.9986	114.0 (16.8)	96.6 (13.3)	88.0 (6.4)
69	Flufenoxuron	Lamb	0.001	0.9987	92.2 (6.8)	88.9 (5.5)	91.3 (3.7)
		Duck meat	0.001	0.9987	88.5 (11.9)	87.6 (5.1)	82.3 (18.1)
		Quail egg	0.001	0.9959	95.0 (5.4)	89.0 (16.0)	90.9 (7.1)
		Mealworm	0.001	0.9989	94.1 (3.6)	90.0 (4.1)	93.6 (4.1)
		Edible frog	0.001	0.9971	110.8 (7.2)	100.9 (9.3)	102.3 (6.6)
70	Flumethrin	Lamb	0.04	0.9643	-	-	103.4 (10.9)
		Duck meat	0.02	0.9970	-	99.6 (48.0)	75.3 (18.3)
		Quail egg	0.1	0.9982	-	-	77.4 (13.0)
		Mealworm	0.1	0.9925	-	-	74.6 (16.3)
		Edible frog	0.04	0.9914	-	-	107.9 (11.6)
71	Flumioxazin	Lamb	0.02	0.9707	-	97.1 (30.4)	103.3 (15.6)
		Duck meat	0.01	0.9867	83.7 (24.1)	91.3 (16.1)	99.8 (12.6)
		Quail egg	0.01	0.9913	107.7 (20.6)	107.9 (21.1)	100.7 (7.2)
		Mealworm	0.004	0.9965	87.0 (29.8)	90.1 (21.2)	114.9 (8.4)
		Edible frog	0.01	0.9807	97.3 (18.2)	86.6 (20.0)	92.2 (9.2)

No.	Analyte	Matrix	LOQ (mg/kg)	r^2	Recovery at 0.01 mg/kg (%, RSD %)	Recovery at 0.02 mg/kg (%, RSD %)	Recovery at 0.1 mg/kg (%, RSD %)
72	Fluopicolide	Lamb	0.001	0.9997	103.0 (4.0)	102.3 (3.8)	96.0 (13.3)
		Duck meat	0.001	0.9984	102.8 (3.1)	102.2 (5.2)	85.9 (15.3)
		Quail egg	0.001	0.9971	99.4 (5.1)	100.2 (8.0)	97.4 (2.1)
		Mealworm	0.002	0.9986	107.0 (8.4)	111.2 (3.8)	93.9 (15.6)
		Edible frog	0.001	0.9962	110.8 (11.7)	106.2 (5.1)	100.4 (4.0)
73	Fluralaner	Lamb	0.001	0.9952	102.6 (10.7)	104.2 (3.7)	103.1 (5.3)
		Duck meat	0.001	0.9932	91.4 (10.3)	91.3 (14.8)	97.0 (4.3)
		Quail egg	0.001	0.9938	106.1 (5.3)	90.4 (14.8)	99.4 (1.9)
		Mealworm	0.001	0.9943	109.6 (4.7)	104.1 (19.0)	97.0 (11.2)
		Edible frog	0.001	0.9919	101.6 (9.7)	91.8 (5.6)	97.4 (8.2)
74	Flutolanil	Lamb	0.001	0.9972	91.7 (15.8)	101.4 (3.8)	97.5 (6.0)
		Duck meat	0.001	0.9977	98.9 (7.4)	95.7 (5.3)	95.5 (5.9)
		Quail egg	0.001	0.9964	95.7 (9.1)	93.6 (5.7)	95.5 (5.7)
		Mealworm	0.001	0.9979	100.4 (10.4)	102.6 (3.7)	102.3 (8.5)
		Edible frog	0.001	0.9938	109.8 (12.0)	104.3 (3.3)	96.9 (4.7)
75	Flutriafol	Lamb	0.001	0.9981	94.7 (7.5)	93.3 (5.9)	97.6 (2.4)
		Duck meat	0.001	0.9971	94.8 (10.3)	96.0 (5.3)	98.0 (4.3)
		Quail egg	0.001	0.9972	92.3 (7.0)	93.2 (5.8)	95.4 (5.1)
		Mealworm	0.001	0.9980	94.0 (10.7)	94.2 (2.6)	99.4 (5.3)
		Edible frog	0.001	0.9944	112.2 (11.5)	101.2 (3.5)	96.7 (1.6)
76	Fluxapyroxad	Lamb	0.001	0.9971	92.5 (17.0)	103.2 (2.9)	92.4 (18.7)
		Duck meat	0.001	0.9958	103.3 (8.0)	102.0 (6.6)	94.9 (7.1)
		Quail egg	0.001	0.9972	94.7 (11.1)	94.4 (8.2)	97.9 (6.9)
		Mealworm	0.001	0.9942	114.6 (16.5)	111.8 (5.0)	115.0 (9.2)
		Edible frog	0.001	0.9959	96.3 (15.6)	102.3 (7.1)	92.7 (7.3)
77	Imazalil	Lamb	0.001	0.9984	92.3 (4.8)	89.8 (1.3)	91.5 (6.2)
		Duck meat	0.001	0.9972	96.0 (9.3)	91.9 (5.3)	91.3 (3.0)
		Quail egg	0.001	0.9956	92.7 (10.1)	95.8 (3.8)	93.7 (7.3)
		Mealworm	0.001	0.9991	85.7 (5.0)	85.5 (4.8)	89.1 (4.9)
		Edible frog	0.001	0.9930	110.2 (8.4)	102.2 (4.0)	93.8 (8.1)
78	Indoxacarb	Lamb	0.001	0.9956	96.7 (20.5)	93.7 (16.7)	96.8 (9.2)
		Duck meat	0.001	0.9942	97.4 (12.6)	96.6 (11.0)	100.6 (7.7)
		Quail egg	0.001	0.9966	97.9 (20.4)	104.7 (8.5)	97.9 (5.2)
		Mealworm	0.001	0.9942	97.9 (11.5)	97.8 (7.5)	103.5 (5.5)
		Edible frog	0.001	0.9912	112.8 (13.2)	101.0 (9.1)	102.9 (7.1)
79	Isofetamid	Lamb	0.001	0.9998	96.0 (11.4)	93.1 (20.0)	98.1 (1.9)
		Duck meat	0.001	0.9927	93.0 (13.3)	92.8 (9.1)	98.0 (4.3)
		Quail egg	0.001	0.9996	98.1 (5.7)	97.3 (4.2)	96.5 (1.1)
		Mealworm	0.001	0.9921	101.6 (0.0)	96.5 (18.9)	104.5 (3.6)
		Edible frog	0.001	0.9989	97.8 (28.7)	99.0 (6.0)	101.0 (2.9)
80	Isoprocarb	Lamb	0.001	0.9988	100.3 (8.2)	95.7 (7.1)	98.5 (4.3)
		Duck meat	0.001	0.9980	97.4 (6.2)	94.7 (5.7)	95.1 (4.5)
		Quail egg	0.001	0.9967	97.5 (2.6)	92.9 (4.5)	95.6 (5.2)
		Mealworm	0.001	0.9975	102.5 (2.1)	98.7 (4.9)	99.3 (2.6)
		Edible frog	0.001	0.9974	114.1 (12.8)	102.6 (6.0)	100.4 (3.5)

No.	Analyte	Matrix	LOQ (mg/kg)	r^2	Recovery at 0.01 mg/kg (%, RSD %)	Recovery at 0.02 mg/kg (%, RSD %)	Recovery at 0.1 mg/kg (%, RSD %)
81	Isoprothiolane	Lamb	0.001	0.9979	108.4 (4.0)	101.0 (1.7)	93.5 (8.0)
		Duck meat	0.001	0.9968	99.4 (7.0)	100.8 (6.3)	95.2 (2.7)
		Quail egg	0.001	0.9984	98.1 (3.7)	89.7 (9.5)	93.5 (3.4)
		Mealworm	0.001	0.9905	103.9 (9.2)	99.7 (3.0)	100.2 (8.5)
		Edible frog	0.001	0.9953	103.8 (17.0)	105.7 (5.3)	100.3 (4.7)
82	Isopyrazam	Lamb	0.001	0.9904	101.0 (3.9)	100.3 (18.3)	95.0 (13.0)
		Duck meat	0.001	0.9964	93.0 (3.5)	92.7 (9.4)	82.4 (11.7)
		Quail egg	0.001	0.9923	96.8 (7.0)	99.4 (8.7)	96.9 (4.0)
		Mealworm	0.001	0.9916	88.6 (13.4)	99.0 (11.6)	102.2 (4.7)
		Edible frog	0.001	0.9933	102.3 (7.8)	97.4 (5.1)	91.2 (7.2)
83	Ivermectin	Lamb	0.004	0.9967	85.4 (19.1)	98.0 (21.3)	97.6 (12.2)
		Duck meat	0.004	0.9938	89.3 (12.9)	91.1 (14.0)	82.6 (14.1)
		Quail egg	0.01	0.9875	120.0 (7.2)	97.9 (16.8)	77.1 (19.3)
		Mealworm	0.01	0.9919	105.7 (18.9)	88.0 (3.5)	75.6 (19.6)
		Edible frog	0.004	0.9956	91.8 (13.6)	88.7 (19.4)	77.5 (14.5)
84	Kresoxim-methyl	Lamb	0.002	0.9930	103.3 (16.2)	92.2 (20.8)	85.4 (18.2)
		Duck meat	0.001	0.9885	110.4 (16.5)	90.2 (17.5)	105.5 (6.4)
		Quail egg	0.001	0.9945	83.8 (28.3)	86.4 (21.8)	81.2 (18.9)
		Mealworm	0.004	0.9922	106.7 (17.1)	116.1 (15.3)	95.3 (19.1)
		Edible frog	0.001	0.9963	95.4 (23.0)	76.3 (21.6)	70.3 (28.5)
85	Lufenuron	Lamb	0.001	0.9982	96.8 (11.8)	89.1 (5.1)	82.7 (7.4)
		Duck meat	0.001	0.9959	102.6 (10.9)	95.8 (8.1)	93.2 (9.6)
		Quail egg	0.001	0.9978	81.7 (20.9)	85.4 (9.0)	92.2 (4.8)
		Mealworm	0.002	0.9979	92.0 (11.8)	98.3 (6.0)	100.6 (6.5)
		Edible frog	0.001	0.9910	107.6 (19.4)	94.6 (8.2)	94.9 (9.7)
86	Malathion	Lamb	0.001	0.9973	89.2 (13.0)	86.3 (6.6)	94.2 (2.9)
		Duck meat	0.001	0.9992	91.9 (9.4)	94.0 (8.3)	94.5 (1.9)
		Quail egg	0.001	0.9975	97.0 (8.1)	99.9 (3.1)	97.1 (4.4)
		Mealworm	0.001	0.9985	100.2 (5.4)	107.6 (4.5)	99.6 (3.6)
		Edible frog	0.001	0.9980	108.6 (9.7)	99.5 (6.7)	100.7 (3.6)
87	Metaflumizone-E	Lamb	0.001	0.9954	95.6 (16.6)	104.5 (4.7)	98.8 (15.4)
		Duck meat	0.001	0.9957	94.1 (12.0)	92.2 (10.5)	85.5 (18.6)
		Quail egg	0.001	0.9937	92.8 (6.9)	97.8 (10.5)	102.5 (7.3)
		Mealworm	0.001	0.9902	87.4 (20.7)	101.9 (7.3)	94.6 (15.8)
		Edible frog	0.001	0.9996	96.2 (9.2)	93.0 (7.8)	80.2 (11.5)
88	Metaflumizone-Z	Lamb	0.001	0.9973	100.5 (6.4)	93.4 (9.1)	94.6 (6.2)
		Duck meat	0.001	0.9988	94.7 (8.8)	95.4 (5.7)	94.5 (5.7)
		Quail egg	0.001	0.9988	95.3 (8.8)	92.0 (5.7)	99.5 (4.6)
		Mealworm	0.001	0.9971	100.6 (5.6)	98.6 (2.3)	103.2 (1.4)
		Edible frog	0.001	0.9957	103.7 (13.4)	96.7 (2.4)	95.0 (3.7)
89	Methamidophos	Lamb	0.001	0.9939	86.1 (4.6)	86.0 (11.6)	88.6 (3.2)
		Duck meat	0.001	0.9963	90.7 (4.1)	92.0 (6.6)	85.0 (4.5)
		Quail egg	0.001	0.9984	83.3 (15.1)	82.9 (9.0)	85.2 (2.5)
		Mealworm	0.002	0.9931	82.7 (12.9)	77.4 (5.3)	77.6 (2.3)
		Edible frog	0.001	0.9993	111.3 (13.8)	91.4 (10.0)	87.1 (7.2)

No.	Analyte	Matrix	LOQ (mg/kg)	r^2	Recovery at 0.01 mg/kg (%, RSD %)	Recovery at 0.02 mg/kg (%, RSD %)	Recovery at 0.1 mg/kg (%, RSD %)
90	Methiocarb	Lamb	0.001	0.9968	92.5 (6.7)	90.4 (8.6)	94.8 (10.8)
		Duck meat	0.001	0.9964	100.4 (5.0)	92.4 (6.3)	87.8 (5.4)
		Quail egg	0.001	0.9944	94.3 (4.2)	95.2 (5.0)	99.0 (2.1)
		Mealworm	0.001	0.9943	100.9 (6.8)	101.8 (11.9)	93.6 (13.1)
		Edible frog	0.001	0.9976	107.8 (18.7)	98.9 (6.5)	99.4 (3.6)
91	Methomyl	Lamb	0.001	0.9942	100.5 (8.9)	88.4 (13.1)	96.2 (3.4)
		Duck meat	0.001	0.9944	103.4 (10.0)	97.9 (9.4)	99.7 (5.1)
		Quail egg	0.001	0.9980	101.2 (4.5)	99.6 (4.9)	95.4 (7.7)
		Mealworm	0.001	0.9971	99.1 (11.9)	106.5 (6.2)	105.5 (6.0)
		Edible frog	0.001	0.9931	107.4 (12.8)	102.4 (3.0)	103.2 (8.6)
92	Methoprene	Lamb	0.001	0.9993	73.6 (21.9)	78.7 (11.5)	67.2 (21.0)
		Duck meat	0.002	0.9953	65.7 (8.2)	80.2 (9.7)	81.0 (11.0)
		Quail egg	0.001	0.9988	83.7 (10.0)	73.0 (16.2)	78.4 (6.8)
		Mealworm	0.002	0.9970	88.9 (18.1)	72.3 (21.9)	75.0 (7.6)
		Edible frog	0.001	0.9971	86.5 (7.5)	83.4 (17.0)	88.2 (5.8)
93	Methoxyfenozide	Lamb	0.001	0.9986	100.0 (2.8)	91.0 (7.1)	83.6 (20.4)
		Duck meat	0.001	0.9972	104.0 (6.1)	104.2 (6.4)	93.4 (13.8)
		Quail egg	0.001	0.9978	97.5 (9.2)	95.6 (6.7)	97.4 (3.5)
		Mealworm	0.001	0.9971	102.0 (6.9)	102.3 (4.8)	101.4 (7.0)
		Edible frog	0.001	0.9974	103.5 (10.2)	98.3 (2.2)	99.2 (4.0)
94	Metrafenone	Lamb	0.001	0.9937	92.7 (3.5)	80.8 (11.5)	94.5 (5.2)
		Duck meat	0.001	0.9927	73.6 (14.6)	72.6 (19.2)	88.4 (4.3)
		Quail egg	0.001	0.9981	86.2 (8.6)	83.5 (5.9)	90.6 (2.7)
		Mealworm	0.001	0.9989	95.2 (8.9)	90.2 (14.6)	93.7 (5.9)
		Edible frog	0.001	1.0000	99.9 (8.9)	91.3 (9.7)	97.3 (3.8)
95	Monocrotophos	Lamb	0.001	0.9964	102.5 (11.2)	91.9 (2.6)	99.9 (4.3)
		Duck meat	0.001	0.9951	100.6 (15.4)	98.3 (3.6)	101.1 (6.3)
		Quail egg	0.001	0.9936	100.1 (5.9)	102.6 (6.9)	98.8 (6.8)
		Mealworm	0.001	0.9953	103.0 (3.6)	103.7 (10.7)	108.9 (7.0)
		Edible frog	0.001	0.9939	109.2 (13.5)	100.6 (8.2)	93.7 (10.8)
96	Myclobutanil	Lamb	0.001	0.9981	90.7 (18.1)	81.5 (10.9)	79.9 (25.3)
		Duck meat	0.001	0.9936	94.0 (18.9)	102.6 (7.1)	98.4 (11.1)
		Quail egg	0.002	0.9941	74.5 (14.0)	92.4 (9.3)	92.3 (10.3)
		Mealworm	0.001	0.9928	100.1 (13.3)	90.7 (17.3)	109.5 (6.4)
		Edible frog	0.001	0.9937	115.9 (15.0)	114.0 (5.9)	109.3 (4.1)
97	Novaluron	Lamb	0.001	0.9967	97.9 (5.9)	94.2 (7.5)	99.6 (4.2)
		Duck meat	0.001	0.9974	95.5 (9.8)	93.5 (7.9)	93.4 (3.8)
		Quail egg	0.001	0.9976	74.5 (14.0)	84.6 (11.4)	97.3 (1.2)
		Mealworm	0.001	0.9957	99.7 (0.0)	96.1 (4.8)	99.7 (4.9)
		Edible frog	0.001	0.9915	101.1 (7.2)	93.2 (10.6)	96.6 (8.0)
98	Orysastrobin	Lamb	0.001	0.9956	99.6 (6.3)	92.8 (4.7)	97.6 (7.1)
		Duck meat	0.001	0.9989	99.2 (4.0)	95.7 (5.0)	96.4 (2.3)
		Quail egg	0.001	0.9986	95.5 (4.9)	93.8 (2.4)	99.1 (4.4)
		Mealworm	0.001	0.9989	104.0 (6.6)	104.6 (5.0)	104.7 (7.7)
		Edible frog	0.001	0.9971	105.9 (9.3)	97.8 (5.8)	97.6 (2.6)

No.	Analyte	Matrix	LOQ (mg/kg)	r^2	Recovery at 0.01 mg/kg (%, RSD %)	Recovery at 0.02 mg/kg (%, RSD %)	Recovery at 0.1 mg/kg (%, RSD %)
99	Oxamyl	Lamb	0.001	0.9954	101.2 (10.1)	102.9 (4.4)	101.6 (10.3)
		Duck meat	0.002	0.9902	112.1 (1.7)	108.6 (10.8)	113.1 (4.2)
		Quail egg	0.001	0.9961	97.9 (8.8)	95.4 (5.5)	92.3 (5.5)
		Mealworm	0.001	0.9986	104.4 (6.2)	96.6 (12.0)	104.5 (6.1)
		Edible frog	0.001	0.9952	112.4 (10.4)	99.6 (7.3)	101.7 (2.9)
100	Oxathiapiprolin	Lamb	0.001	0.9972	101.1 (16.1)	95.6 (7.0)	96.0 (3.2)
		Duck meat	0.001	0.9980	112.9 (11.4)	101.5 (7.8)	93.1 (6.9)
		Quail egg	0.001	0.9974	102.0 (14.0)	99.3 (12.7)	103.6 (6.1)
		Mealworm	0.002	0.9961	88.5 (11.4)	97.2 (5.8)	101.3 (11.0)
		Edible frog	0.001	0.9974	111.0 (23.6)	101.4 (8.1)	98.1 (6.3)
101	Penconazole	Lamb	0.001	0.9994	88.0 (15.2)	90.3 (6.1)	90.8 (5.2)
		Duck meat	0.001	0.9945	91.9 (9.6)	85.9 (20.9)	95.7 (5.9)
		Quail egg	0.001	0.9984	74.4 (23.4)	85.9 (18.9)	85.2 (9.8)
		Mealworm	0.001	0.9973	84.0 (10.3)	86.9 (9.5)	92.7 (3.4)
		Edible frog	0.001	0.9990	106.6 (9.8)	92.2 (15.1)	80.5 (21.2)
102	Pencycuron	Lamb	0.001	0.9925	85.7 (6.8)	76.9 (11.9)	90.5 (7.7)
		Duck meat	0.001	0.9900	94.4 (5.0)	90.7 (3.4)	74.5 (21.2)
		Quail egg	0.001	0.9958	94.5 (12.2)	87.6 (16.9)	77.5 (20.5)
		Mealworm	0.002	0.9988	82.7 (20.3)	88.4 (15.6)	88.8 (13.7)
		Edible frog	0.001	0.9996	106.2 (10.2)	99.7 (9.3)	88.2 (21.1)
103	Pendimethalin	Lamb	0.001	0.9954	82.0 (11.3)	83.4 (3.5)	82.4 (14.4)
		Duck meat	0.001	0.9925	82.5 (14.7)	83.7 (6.1)	86.0 (5.1)
		Quail egg	0.001	0.9966	86.7 (7.7)	82.3 (5.9)	78.1 (9.0)
		Mealworm	0.004	0.9991	93.8 (3.0)	72.9 (9.0)	75.4 (11.1)
		Edible frog	0.001	0.9916	97.9 (13.3)	96.0 (7.7)	95.5 (7.7)
104	Phorate	Lamb	0.001	0.9904	68.0 (27.7)	79.9 (13.2)	89.0 (7.2)
		Duck meat	0.001	0.9927	89.3 (6.9)	92.4 (9.6)	90.0 (2.6)
		Quail egg	0.001	0.9962	80.9 (10.7)	85.3 (9.9)	91.7 (4.2)
		Mealworm	0.004	0.9989	104.6 (20.7)	99.9 (16.2)	93.1 (4.7)
		Edible frog	0.001	0.9952	99.3 (28.6)	91.2 (18.6)	90.1 (10.7)
105	Phorate-sulfone	Lamb	0.001	0.9959	95.4 (11.0)	96.6 (9.2)	99.4 (3.1)
		Duck meat	0.001	0.9992	97.8 (6.7)	97.7 (9.2)	96.1 (3.6)
		Quail egg	0.001	0.9985	90.7 (7.4)	90.3 (7.0)	98.8 (4.0)
		Mealworm	0.001	0.9981	102.0 (5.5)	105.6 (8.3)	107.1 (2.9)
		Edible frog	0.001	0.9956	108.2 (12.3)	107.4 (8.4)	100.2 (4.6)
106	Phorate-sulfoxide	Lamb	0.001	0.9974	101.9 (1.9)	97.6 (4.8)	101.8 (4.7)
		Duck meat	0.001	0.9990	102.8 (3.1)	100.0 (4.2)	97.1 (2.4)
		Quail egg	0.001	0.9976	93.1 (3.2)	96.5 (1.7)	98.4 (5.4)
		Mealworm	0.001	0.9988	106.1 (5.0)	101.6 (6.3)	102.1 (3.7)
		Edible frog	0.001	0.9978	110.3 (8.9)	102.0 (2.8)	97.4 (5.3)
107	Phorate-oxon	Lamb	0.001	0.9987	99.8 (6.5)	98.2 (2.7)	98.0 (6.9)
		Duck meat	0.001	0.9971	101.2 (3.8)	96.6 (5.3)	92.8 (3.6)
		Quail egg	0.001	0.9953	93.3 (5.8)	97.6 (7.5)	100.9 (3.1)
		Mealworm	0.001	0.9990	105.8 (2.7)	105.4 (5.6)	102.3 (5.1)
		Edible frog	0.001	0.9979	107.6 (8.9)	104.6 (2.1)	100.0 (1.6)

No.	Analyte	Matrix	LOQ (mg/kg)	r^2	Recovery at 0.01 mg/kg (%, RSD %)	Recovery at 0.02 mg/kg (%, RSD %)	Recovery at 0.1 mg/kg (%, RSD %)
108	Phorate-oxon-sulfone	Lamb	0.001	0.9956	109.1 (6.6)	101.6 (4.9)	101.5 (3.3)
		Duck meat	0.001	0.9970	88.4 (11.2)	98.6 (6.9)	98.5 (2.6)
		Quail egg	0.001	0.9959	97.1 (3.6)	95.6 (4.6)	100.2 (4.9)
		Mealworm	0.001	0.9988	105.2 (7.2)	99.4 (5.3)	107.7 (2.6)
		Edible frog	0.001	0.9942	111.2 (16.2)	105.9 (2.4)	101.8 (3.4)
109	Phorate-oxon-sulfoxide	Lamb	0.001	0.9967	96.4 (5.8)	95.9 (3.0)	101.1 (4.1)
		Duck meat	0.001	0.9953	99.5 (21.2)	100.0 (9.2)	98.2 (4.9)
		Quail egg	0.001	0.9990	92.2 (2.4)	97.3 (9.1)	97.9 (2.4)
		Mealworm	0.001	0.9975	101.9 (7.6)	99.4 (6.4)	102.2 (9.1)
		Edible frog	0.002	0.9979	110.8 (9.8)	104.6 (4.5)	97.3 (3.8)
110	Phosalone	Lamb	0.001	0.9979	98.3 (5.6)	97.1 (4.8)	93.3 (3.9)
		Duck meat	0.001	0.9961	95.0 (5.1)	95.8 (5.3)	94.7 (5.0)
		Quail egg	0.001	0.9965	99.2 (6.7)	98.1 (12.3)	101.5 (4.5)
		Mealworm	0.001	0.9940	92.1 (7.4)	96.2 (5.5)	98.9 (2.9)
		Edible frog	0.001	0.9911	107.0 (12.3)	97.8 (8.0)	100.2 (3.6)
111	Phoxim	Lamb	0.001	0.9924	86.0 (2.9)	92.8 (6.5)	79.4 (12.8)
		Duck meat	0.001	0.9981	90.9 (20.8)	98.2 (8.6)	94.8 (10.5)
		Quail egg	0.001	0.9947	107.3 (11.8)	95.4 (8.6)	107.1 (11.6)
		Mealworm	0.001	0.9909	84.5 (12.5)	86.8 (3.1)	105.3 (6.3)
		Edible frog	0.001	0.9976	99.3 (3.4)	106.6 (9.1)	102.8 (10.6)
112	Picoxystrobin	Lamb	0.001	0.9919	97.7 (9.1)	100.8 (4.1)	100.8 (13.5)
		Duck meat	0.001	0.9979	101.0 (3.3)	90.6 (18.7)	70.5 (20.2)
		Quail egg	0.001	0.9992	95.8 (5.9)	96.6 (8.0)	99.7 (3.0)
		Mealworm	0.001	0.9970	99.7 (4.4)	94.5 (7.0)	102.1 (2.6)
		Edible frog	0.001	0.9985	106.0 (10.0)	93.4 (3.4)	95.0 (2.9)
113	Piperonyl butoxide	Lamb	0.001	0.9991	89.0 (7.0)	86.2 (11.2)	85.7 (13.9)
		Duck meat	0.001	0.9947	100.2 (3.9)	75.7 (19.7)	83.2 (13.1)
		Quail egg	0.001	0.9938	91.9 (4.2)	87.4 (15.3)	96.2 (3.3)
		Mealworm	0.001	0.9953	95.6 (6.8)	93.9 (10.3)	88.1 (12.3)
		Edible frog	0.001	0.9962	90.0 (17.6)	92.5 (5.0)	92.1 (7.8)
114	Pirimiphos-methyl	Lamb	0.001	0.9974	89.8 (4.9)	96.3 (2.6)	87.0 (6.0)
		Duck meat	0.001	0.9973	86.8 (6.7)	88.7 (5.6)	88.8 (6.5)
		Quail egg	0.001	0.9983	98.3 (1.9)	91.8 (4.4)	96.0 (7.1)
		Mealworm	0.001	0.9979	89.0 (5.3)	86.6 (8.1)	83.6 (6.2)
		Edible frog	0.001	0.9944	105.6 (10.6)	98.9 (4.9)	96.6 (4.7)
115	Profenofos	Lamb	0.001	0.9966	89.6 (7.9)	84.7 (10.0)	93.7 (5.1)
		Duck meat	0.001	0.9985	87.5 (5.3)	85.8 (6.5)	86.6 (4.2)
		Quail egg	0.001	0.9982	91.2 (2.1)	87.6 (4.3)	85.3 (9.2)
		Mealworm	0.001	0.9950	90.2 (3.9)	79.2 (14.5)	89.5 (6.2)
		Edible frog	0.001	0.9972	117.3 (10.6)	105.7 (5.5)	110.3 (5.7)
116	Propamocarb	Lamb	0.001	0.9976	80.9 (4.5)	81.3 (3.6)	93.8 (2.7)
		Duck meat	0.001	0.9945	81.0 (7.4)	91.0 (5.3)	91.8 (1.8)
		Quail egg	0.001	0.9946	81.2 (4.0)	88.1 (5.1)	85.8 (2.7)
		Mealworm	0.001	0.9974	76.0 (4.8)	81.8 (6.3)	84.7 (8.3)
		Edible frog	0.001	0.9965	85.7 (5.6)	86.0 (6.3)	85.9 (5.3)

No.	Analyte	Matrix	LOQ (mg/kg)	r^2	Recovery at 0.01 mg/kg (%, RSD %)	Recovery at 0.02 mg/kg (%, RSD %)	Recovery at 0.1 mg/kg (%, RSD %)
117	Propargite	Lamb	0.001	0.9981	87.8 (3.4)	87.4 (4.4)	86.7 (7.2)
		Duck meat	0.001	0.9910	86.0 (9.4)	80.6 (13.5)	88.2 (8.9)
		Quail egg	0.001	0.9965	92.2 (11.3)	84.0 (10.4)	90.8 (9.3)
		Mealworm	0.001	0.9983	96.1 (3.2)	93.0 (1.3)	92.1 (4.1)
		Edible frog	0.001	0.9917	102.6 (7.5)	94.3 (5.9)	96.3 (4.4)
118	Propoxur	Lamb	0.001	0.9990	95.5 (5.8)	95.0 (7.6)	97.5 (3.5)
		Duck meat	0.001	0.9990	97.9 (3.7)	96.2 (3.4)	96.4 (1.9)
		Quail egg	0.001	0.9987	92.4 (4.1)	93.9 (3.7)	96.3 (3.8)
		Mealworm	0.001	0.9962	105.3 (2.7)	102.3 (3.5)	105.2 (7.1)
		Edible frog	0.001	0.9976	109.5 (7.5)	99.1 (4.1)	102.6 (2.3)
119	Pydiflumetofen	Lamb	0.001	0.9940	101.7 (3.1)	90.2 (13.7)	91.1 (19.8)
		Duck meat	0.001	0.9941	94.3 (9.6)	91.5 (12.8)	82.1 (21.0)
		Quail egg	0.001	0.9974	96.6 (10.6)	93.9 (8.8)	82.7 (21.7)
		Mealworm	0.001	0.9994	86.0 (23.7)	89.2 (3.8)	91.1 (19.3)
		Edible frog	0.001	0.9974	105.1 (13.7)	99.0 (5.1)	94.4 (9.9)
120	Pyraclostrobin	Lamb	0.001	0.9966	103.3 (3.2)	101.9 (2.8)	97.2 (5.8)
		Duck meat	0.001	0.9956	90.6 (8.8)	94.7 (2.0)	92.2 (6.3)
		Quail egg	0.001	0.9969	97.9 (7.7)	95.2 (6.6)	94.9 (8.2)
		Mealworm	0.001	0.9964	96.0 (10.7)	94.1 (5.5)	98.2 (5.8)
		Edible frog	0.001	0.9955	112.6 (10.4)	102.3 (4.2)	97.1 (4.8)
121	Pyrimethanil	Lamb	0.001	0.9946	84.7 (14.4)	98.5 (15.6)	90.4 (6.1)
		Duck meat	0.001	0.9980	73.0 (23.6)	81.6 (8.3)	87.4 (11.4)
		Quail egg	0.001	0.9944	97.9 (22.6)	81.9 (17.4)	91.9 (3.5)
		Mealworm	0.002	0.9947	78.9 (21.2)	71.7 (15.9)	72.2 (11.1)
		Edible frog	0.001	0.9988	105.2 (22.0)	84.6 (19.7)	91.3 (9.9)
122	Pyrimethanil-4-hydroxy	Lamb	0.001	0.9985	7.5 (48.8)	5.7 (17.6)	5.4 (35.2)
		Duck meat	0.001	0.9957	69.1 (15.1)	70.1 (11.8)	70.4 (6.9)
		Quail egg	0.001	0.9982	89.5 (17.7)	85.3 (6.6)	90.0 (5.7)
		Mealworm	0.001	0.9900	82.8 (8.3)	89.9 (10.3)	79.5 (8.1)
		Edible frog	0.001	0.9950	102.8 (8.2)	90.2 (13.6)	94.8 (4.2)
123	Pyrimethanil-5-hydroxy	Lamb	0.004	0.9972	6.4 (27.3)	1.9 (78.1)	0.6 (25.4)
		Duck meat	0.001	0.9962	60.5 (3.7)	46.6 (9.8)	46.7 (13.4)
		Quail egg	0.001	0.9940	101.2 (8.7)	98.2 (9.6)	91.6 (4.1)
		Mealworm	0.001	0.9923	80.0 (8.2)	86.0 (9.3)	82.5 (5.4)
		Edible frog	0.001	0.9918	118.9 (19.2)	100.8 (7.8)	90.0 (3.2)
124	Pyriproxyfen	Lamb	0.004	0.9956	94.9 (12.7)	88.2 (11.9)	87.4 (21.7)
		Duck meat	0.001	0.9962	86.9 (2.1)	82.4 (10.4)	82.1 (7.0)
		Quail egg	0.001	0.9943	78.4 (11.6)	85.8 (5.8)	80.3 (6.2)
		Mealworm	0.001	0.9986	78.7 (14.2)	78.3 (4.1)	75.8 (8.2)
		Edible frog	0.001	0.9971	103.9 (9.7)	88.2 (15.0)	101.7 (5.0)
125	Saflufenacil	Lamb	0.001	0.9977	51.1 (20.7)	51.9 (10.4)	59.0 (5.4)
		Duck meat	0.004	0.9974	60.4 (5.2)	51.9 (7.7)	54.9 (8.9)
		Quail egg	0.001	0.9964	66.6 (7.3)	70.0 (3.6)	70.5 (7.0)
		Mealworm	0.001	0.9990	70.2 (13.5)	77.0 (2.9)	79.0 (5.8)
		Edible frog	0.001	0.9978	49.7 (18.3)	50.4 (8.0)	52.5 (7.5)

No.	Analyte	Matrix	LOQ (mg/kg)	r^2	Recovery at 0.01 mg/kg (%, RSD %)	Recovery at 0.02 mg/kg (%, RSD %)	Recovery at 0.1 mg/kg (%, RSD %)
126	Sedaxane	Lamb	0.001	0.9976	99.7 (6.0)	99.2 (4.7)	81.8 (23.6)
		Duck meat	0.001	0.9976	102.1 (4.5)	97.3 (9.7)	96.6 (2.5)
		Quail egg	0.001	0.9991	90.7 (4.3)	92.9 (2.7)	101.4 (2.5)
		Mealworm	0.001	0.9976	105.9 (4.7)	99.5 (3.6)	99.6 (15.1)
		Edible frog	0.001	0.9906	110.2 (10.2)	103.9 (3.7)	99.8 (7.0)
127	Simazine	Lamb	0.001	0.9978	94.1 (11.8)	94.4 (5.6)	94.4 (3.0)
		Duck meat	0.001	0.9982	93.2 (5.3)	96.6 (5.2)	91.3 (2.7)
		Quail egg	0.001	0.9970	100.0 (4.4)	97.5 (3.6)	96.7 (6.4)
		Mealworm	0.001	0.9987	87.0 (10.7)	93.2 (8.8)	97.1 (3.5)
		Edible frog	0.001	0.9986	109.9 (8.1)	101.2 (5.9)	95.2 (3.4)
128	Spinosyn-A	Lamb	0.001	0.9956	77.6 (2.9)	81.9 (6.5)	77.5 (4.7)
		Duck meat	0.001	0.9955	80.8 (6.8)	80.3 (5.7)	82.8 (3.7)
		Quail egg	0.001	0.9960	85.1 (8.5)	83.8 (5.5)	85.7 (7.8)
		Mealworm	0.001	0.9987	81.7 (4.2)	83.8 (5.6)	84.9 (5.5)
		Edible frog	0.001	0.9934	92.7 (6.8)	82.9 (1.5)	81.2 (7.5)
129	Spinosyn-D	Lamb	0.001	0.9977	80.6 (5.6)	80.5 (3.0)	81.0 (5.1)
		Duck meat	0.001	0.9995	86.7 (4.7)	78.2 (4.0)	83.6 (3.2)
		Quail egg	0.001	0.9984	81.4 (3.8)	78.1 (4.0)	78.6 (2.0)
		Mealworm	0.001	0.9970	84.2 (6.9)	85.9 (3.8)	90.3 (3.0)
		Edible frog	0.001	0.9958	90.4 (7.7)	81.7 (7.7)	80.2 (8.2)
130	Spiromesifen	Lamb	0.004	0.9973	84.6 (14.7)	91.3 (7.8)	91.1 (13.4)
		Duck meat	0.004	0.9927	97.6 (22.1)	102.5 (14.6)	108.7 (9.7)
		Quail egg	0.001	0.9911	89.2 (13.5)	80.7 (11.9)	85.3 (9.5)
		Mealworm	0.004	0.9953	92.0 (23.8)	81.8 (5.6)	86.5 (5.6)
		Edible frog	0.001	0.9959	105.1 (16.1)	86.9 (11.8)	86.6 (9.3)
131	Sulfoxaflor	Lamb	0.002	0.9941	89.4 (31.6)	91.3 (19.4)	86.7 (12.8)
		Duck meat	0.001	0.9942	91.0 (28.0)	100.0 (8.6)	91.1 (7.1)
		Quail egg	0.001	0.9913	103.6 (16.0)	105.0 (10.5)	100.3 (6.3)
		Mealworm	0.002	0.9928	117.0 (13.6)	112.8 (13.8)	116.9 (8.7)
		Edible frog	0.001	0.9944	111.9 (9.5)	104.9 (17.2)	98.1 (5.8)
132	Tebuconazole	Lamb	0.001	0.9984	99.7 (4.2)	95.6 (8.4)	95.3 (9.8)
		Duck meat	0.001	0.9978	98.4 (9.4)	94.2 (4.7)	80.1 (21.9)
		Quail egg	0.001	0.9965	94.1 (2.5)	95.0 (3.6)	89.8 (7.0)
		Mealworm	0.001	0.9981	91.7 (14.2)	80.7 (21.3)	86.6 (7.8)
		Edible frog	0.001	0.9972	91.1 (27.5)	97.7 (1.2)	97.1 (1.6)
133	Tebufenozide	Lamb	0.001	0.9920	109.3 (6.6)	112.0 (7.6)	98.3 (9.7)
		Duck meat	0.001	0.9957	105.7 (3.4)	89.8 (20.5)	86.4 (21.8)
		Quail egg	0.001	0.9973	104.3 (3.8)	97.8 (9.9)	98.1 (6.3)
		Mealworm	0.001	0.9965	107.1 (3.7)	101.3 (10.7)	111.1 (6.1)
		Edible frog	0.001	0.9963	112.5 (15.8)	93.3 (9.0)	97.6 (2.8)
134	Teflubenzuron	Lamb	0.001	0.9903	85.1 (26.2)	85.1 (7.7)	95.1 (8.9)
		Duck meat	0.002	0.9957	93.5 (12.3)	101.5 (18.9)	96.5 (13.6)
		Quail egg	0.002	0.9914	95.8 (18.7)	79.9 (21.6)	88.2 (9.7)
		Mealworm	0.002	0.9991	85.8 (23.1)	82.0 (20.5)	87.5 (5.5)
		Edible frog	0.002	0.9830	109.2 (13.5)	99.5 (5.6)	106.7 (18.3)

No.	Analyte	Matrix	LOQ (mg/kg)	r^2	Recovery at 0.01 mg/kg (%, RSD %)	Recovery at 0.02 mg/kg (%, RSD %)	Recovery at 0.1 mg/kg (%, RSD %)
135	Tetraconazole	Lamb	0.001	0.9995	89.1 (20.8)	72.3 (37.4)	96.4 (5.3)
		Duck meat	0.001	0.9997	91.9 (13.3)	81.8 (17.9)	94.5 (1.7)
		Quail egg	0.001	0.9969	98.1 (7.2)	99.6 (4.8)	98.0 (3.0)
		Mealworm	0.001	0.9957	111.2 (3.6)	104.9 (10.0)	98.3 (12.4)
		Edible frog	0.001	0.9973	112.4 (6.9)	100.1 (3.2)	98.0 (1.7)
136	Thiacloprid	Lamb	0.001	0.9937	98.9 (15.3)	95.9 (8.9)	99.7 (11.1)
		Duck meat	0.001	0.9921	102.5 (14.2)	98.8 (11.5)	96.4 (5.3)
		Quail egg	0.001	0.9959	99.8 (3.3)	99.6 (5.5)	98.6 (4.7)
		Mealworm	0.001	0.9961	97.9 (5.6)	105.1 (2.9)	103.7 (6.4)
		Edible frog	0.01	0.9849	89.0 (10.8)	95.3 (4.3)	103.9 (2.4)
137	Thiamethoxam	Lamb	0.001	0.9925	106.5 (11.2)	96.1 (8.3)	106.6 (4.0)
		Duck meat	0.001	0.9989	104.0 (9.2)	102.6 (4.4)	96.4 (4.0)
		Quail egg	0.001	0.9974	104.3 (7.2)	103.4 (4.6)	98.3 (5.6)
		Mealworm	0.001	0.9986	96.9 (9.2)	107.6 (4.3)	98.8 (6.9)
		Edible frog	0.001	0.9918	116.5 (12.4)	104.0 (9.5)	97.0 (4.3)
138	Tiadinil	Lamb	0.004	0.9998	62.5 (21.0)	77.3 (8.0)	84.8 (18.5)
		Duck meat	0.004	0.9988	87.3 (23.6)	84.7 (7.4)	89.6 (17.0)
		Quail egg	0.002	0.9909	101.7 (25.7)	101.3 (6.8)	104.2 (9.1)
		Mealworm	0.002	0.9917	79.6 (30.6)	84.6 (12.4)	86.7 (16.4)
		Edible frog	0.002	0.9932	90.1 (9.8)	93.5 (8.0)	93.2 (8.1)
139	Tolfenpyrad	Lamb	0.001	0.9918	85.6 (6.4)	88.7 (3.5)	89.7 (7.3)
		Duck meat	0.01	0.9954	77.4 (9.7)	85.7 (5.0)	85.9 (9.6)
		Quail egg	0.001	0.9942	85.5 (7.3)	80.4 (5.9)	83.1 (12.3)
		Mealworm	0.001	0.9966	78.4 (19.7)	86.3 (11.6)	84.5 (7.7)
		Edible frog	0.001	0.9972	106.2 (14.7)	92.8 (12.7)	96.6 (10.4)
140	Trichlorfon	Lamb	0.04	0.9908	-	-	83.3 (23.7)
		Duck meat	0.04	0.9962	-	-	100.4 (6.6)
		Quail egg	0.04	0.9966	-	-	89.4 (2.8)
		Mealworm	0.04	0.9955	-	-	99.4 (4.2)
		Edible frog	0.04	0.9988	-	-	99.1 (1.8)
141	Tricyclazole	Lamb	0.001	0.9911	97.9 (7.6)	98.0 (4.0)	94.2 (5.1)
		Duck meat	0.001	0.9972	97.1 (7.2)	91.9 (2.7)	88.9 (2.0)
		Quail egg	0.001	0.9982	93.0 (6.4)	94.9 (7.6)	90.8 (6.6)
		Mealworm	0.001	0.9984	84.4 (9.6)	89.0 (6.1)	89.1 (2.4)
		Edible frog	0.001	0.9981	98.4 (15.8)	95.5 (4.3)	87.9 (3.3)
142	Triflumezopyrim	Lamb	0.001	0.9966	88.9 (11.9)	94.7 (13.7)	93.7 (4.5)
		Duck meat	0.001	0.9946	106.7 (16.8)	94.4 (11.4)	99.8 (10.0)
		Quail egg	0.001	0.9934	90.5 (10.2)	91.6 (10.5)	97.3 (8.1)
		Mealworm	0.001	0.9975	102.0 (6.6)	89.7 (5.7)	98.7 (1.8)
		Edible frog	0.001	0.9902	104.1 (17.0)	99.0 (10.4)	100.7 (4.2)
143	Triforine	Lamb	0.01	0.9881	118.4 (31.5)	92.9 (11.7)	94.8 (12.1)
		Duck meat	0.01	0.9775	70.3 (28.4)	82.0 (21.4)	102.8 (18.9)
		Quail egg	0.01	0.9975	60.3 (40.3)	79.5 (21.5)	90.2 (18.5)
		Mealworm	0.01	0.9991	119.1 (18.2)	113.0 (13.7)	98.6 (17.0)
		Edible frog	0.01	0.9858	64.8 (32.0)	99.0 (10.4)	88.3 (18.0)

No.	Analyte	Matrix	LOQ (mg/kg)	r^2	Recovery at 0.01 mg/kg (%, RSD %)	Recovery at 0.02 mg/kg (%, RSD %)	Recovery at 0.1 mg/kg (%, RSD %)
144	Flonicamid	Lamb	0.004	0.9977	99.4 (20.9)	101.7 (16.5)	92.2 (10.9)
		Duck meat	0.002	0.9909	118.8 (31.5)	97.6 (18.5)	100.0 (11.2)
		Quail egg	0.002	0.9962	88.9 (21.0)	86.9 (11.6)	96.4 (18.4)
		Mealworm	0.004	0.9925	118.3 (11.8)	113.5 (7.4)	104.2 (6.1)
		Edible frog	0.004	0.9982	112.3 (10.2)	98.9 (14.7)	98.0 (12.5)
145	Flonicamid-TFNA-AM	Lamb	0.001	0.9919	97.4 (24.7)	103.7 (9.9)	90.5 (7.8)
		Duck meat	0.001	0.9968	96.5 (18.4)	92.6 (14.5)	86.7 (8.3)
		Quail egg	0.001	0.9909	81.8 (24.8)	87.4 (19.9)	92.9 (6.8)
		Mealworm	0.01	0.9912	100.3 (16.5)	94.6 (21.8)	110.9 (5.6)
		Edible frog	0.001	0.9878	80.5 (19.4)	96.1 (9.5)	90.4 (8.1)
146	Imidacloprid	Lamb	0.001	0.9945	85.5 (31.7)	95.3 (11.3)	92.3 (22.0)
		Duck meat	0.002	0.9979	95.3 (10.7)	101.0 (6.1)	95.8 (3.8)
		Quail egg	0.001	0.9963	89.5 (13.8)	100.4 (11.3)	96.3 (3.3)
		Mealworm	0.001	0.9976	107.0 (4.4)	99.7 (6.5)	104.3 (5.7)
		Edible frog	0.001	0.9985	114.9 (9.9)	101.5 (11.9)	96.6 (5.2)
147	Imidacloprid-olefin	Lamb	0.1	0.9931	-	-	72.9 (23.5)
		Duck meat	0.04	0.9775	-	-	89.6 (15.9)
		Quail egg	0.1	0.9821	-	-	72.4 (16.3)
		Mealworm	0.02	0.9914	-	102.9 (48.9)	89.2 (19.8)
		Edible frog	0.04	0.9630	-	-	119.8 (16.8)
148	Imidacloprid-urea	Lamb	0.001	0.9959	84.4 (20.2)	91.1 (6.9)	94.9 (5.2)
		Duck meat	0.002	0.9952	94.4 (5.7)	93.0 (4.0)	88.0 (5.3)
		Quail egg	0.001	0.9976	87.4 (28.4)	85.1 (12.1)	90.2 (8.3)
		Mealworm	0.001	0.9972	81.8 (13.0)	84.1 (4.5)	86.6 (6.0)
		Edible frog	0.001	0.9947	95.5 (13.4)	101.4 (8.6)	88.3 (4.1)
149	Spinetoram-J (XDE-175-J)	Lamb	0.001	0.9973	80.4 (15.4)	79.8 (5.2)	75.2 (11.4)
		Duck meat	0.001	0.9982	83.9 (9.8)	78.8 (3.3)	80.2 (3.5)
		Quail egg	0.001	0.9967	80.7 (5.3)	78.6 (7.5)	79.1 (5.6)
		Mealworm	0.001	0.9984	79.5 (6.5)	77.9 (12.1)	83.8 (4.8)
		Edible frog	0.001	0.9974	97.0 (10.0)	85.8 (11.2)	82.8 (3.6)
150	Spinetoram-L (XDE-175-L)	Lamb	0.001	0.9947	81.3 (6.0)	73.1 (19.9)	76.8 (10.7)
		Duck meat	0.001	0.9974	83.6 (10.5)	78.4 (8.2)	77.3 (4.7)
		Quail egg	0.001	0.9949	64.5 (23.8)	78.1 (9.3)	77.3 (3.5)
		Mealworm	0.001	0.9968	77.9 (13.9)	70.9 (12.1)	76.4 (6.7)
		Edible frog	0.001	0.9909	94.1 (27.8)	75.2 (7.8)	81.8 (6.1)

[1] -, not determined or not available in the final report.

Table S2. Individual GC–MS/MS validation results for pesticide residues in five representative minor-species matrices.

No.	Analyte	Matrix	LOQ (mg/kg)	r^2	Recovery at 0.01 mg/kg (%, RSD %)	Recovery at 0.02 mg/kg (%, RSD %)	Recovery at 0.1 mg/kg (%, RSD %)
1	Aldrin	Lamb	0.006	0.9962	115.6 (2.6)	104.2 (0.4)	92.2 (1.3)
		Duck meat	0.006	0.9907	93.1 (4.0)	92.6 (5.2)	87.3 (3.8)
		Quail egg	0.006	0.9986	91.7 (2.4)	83.3 (1.0)	73.1 (1.5)
		Mealworm	0.006	0.9974	96.5 (6.2)	73.2 (1.9)	76.2 (3.0)
		Edible frog	0.006	0.9995	82.0 (13.3)	84.3 (4.1)	80.6 (3.4)
2	Dieldrin	Lamb	0.006	0.9995	82.9 (12.1)	83.8 (11.6)	84.5 (5.6)
		Duck meat	0.006	0.9944	82.1 (4.1)	83.7 (3.4)	75.5 (2.9)
		Quail egg	0.006	0.9981	79.8 (9.1)	89.9 (4.3)	79.7 (6.7)
		Mealworm	0.006	0.9989	105.9 (5.3)	73.1 (0.5)	80.5 (10.2)
		Edible frog	0.006	0.9995	91.5 (14.8)	89.4 (2.5)	80.7 (2.5)
3	Atrazine	Lamb	0.006	0.9999	94.8 (4.4)	93.8 (2.0)	97.2 (1.2)
		Duck meat	0.006	0.9948	100.0 (6.3)	103.6 (12.6)	106.8 (2.6)
		Quail egg	0.006	0.9997	102.6 (8.0)	105.5 (7.3)	101.0 (0.6)
		Mealworm	0.006	0.9948	107.7 (7.7)	79.8 (11.5)	81.3 (4.6)
		Edible frog	0.006	0.9954	95.6 (6.8)	88.3 (18.6)	80.1 (9.4)
4	BHC-α	Lamb	0.006	0.9952	97.3 (2.3)	97.4 (4.0)	96.1 (2.6)
		Duck meat	0.006	0.9998	96.4 (6.2)	89.4 (1.8)	86.0 (8.7)
		Quail egg	0.006	0.9967	98.6 (0.6)	96.7 (11.6)	99.3 (2.2)
		Mealworm	0.006	0.9989	111.2 (3.1)	73.1 (1.8)	76.0 (6.0)
		Edible frog	0.006	0.9987	85.9 (7.3)	85.1 (1.4)	89.7 (1.7)
5	BHC-β	Lamb	0.006	0.9999	85.8 (4.6)	98.4 (4.3)	99.9 (1.6)
		Duck meat	0.006	0.9964	105.3 (9.9)	95.5 (6.3)	95.1 (7.5)
		Quail egg	0.006	0.9983	105.4 (9.0)	96.6 (8.3)	89.4 (6.4)
		Mealworm	0.006	0.9978	90.6 (14.7)	78.4 (4.2)	82.8 (0.8)
		Edible frog	0.006	0.9987	78.6 (4.5)	83.1 (8.1)	79.9 (6.6)
6	BHC-γ (Lindane)	Lamb	0.006	0.9930	101.9 (3.1)	101.9 (3.4)	94.8 (2.0)
		Duck meat	0.006	0.9983	94.4 (15.9)	103.9 (14.4)	94.4 (12.2)
		Quail egg	0.006	0.9992	117.4 (1.7)	110.8 (9.3)	92.9 (4.9)
		Mealworm	0.006	0.9992	89.7 (11.0)	76.0 (7.2)	78.7 (7.0)
		Edible frog	0.006	0.9987	85.9 (7.3)	85.1 (1.4)	89.7 (1.7)
7	BHC-δ	Lamb	0.006	0.9984	91.8 (3.2)	92.9 (2.7)	98.6 (5.2)
		Duck meat	0.006	0.9908	85.0 (10.8)	92.4 (7.5)	95.5 (4.5)
		Quail egg	0.006	0.9985	109.5 (7.6)	94.1 (2.5)	88.2 (2.5)
		Mealworm	0.006	0.9992	108.3 (9.6)	78.7 (7.0)	77.3 (0.3)
		Edible frog	0.006	0.9992	79.7 (13.6)	79.0 (7.3)	86.5 (9.8)
8	Bifenthrin	Lamb	0.006	0.9974	75.9 (6.0)	78.4 (4.6)	80.3 (1.2)
		Duck meat	0.006	1.0000	84.8 (3.5)	80.4 (2.5)	76.4 (1.8)
		Quail egg	0.006	0.9994	75.7 (6.5)	82.0 (5.1)	73.2 (3.2)
		Mealworm	0.006	0.9927	108.4 (4.5)	80.8 (1.0)	80.9 (0.5)
		Edible frog	0.006	0.9991	89.9 (1.0)	91.4 (0.2)	82.6 (2.0)

No.	Analyte	Matrix	LOQ (mg/kg)	r^2	Recovery at 0.01 mg/kg (%, RSD %)	Recovery at 0.02 mg/kg (%, RSD %)	Recovery at 0.1 mg/kg (%, RSD %)
9	Bitertanol	Lamb	0.006	0.9971	106.1 (5.5)	109.7 (4.6)	82.3 (10.3)
		Duck meat	0.006	0.9997	109.2 (9.1)	110.4 (3.6)	100.3 (5.8)
		Quail egg	0.006	0.9957	94.5 (3.9)	82.2 (4.6)	98.4 (2.8)
		Mealworm	0.006	0.9926	89.5 (6.3)	72.7 (6.4)	87.6 (1.2)
		Edible frog	0.006	0.9951	79.6 (14.4)	77.5 (3.5)	85.7 (12.4)
10	Buprofezin	Lamb	0.006	0.9967	80.1 (7.9)	81.9 (6.5)	91.1 (5.8)
		Duck meat	0.006	0.9995	95.1 (18.5)	91.2 (10.7)	88.0 (4.2)
		Quail egg	0.006	0.9988	94.0 (15.3)	86.4 (6.4)	81.1 (4.0)
		Mealworm	0.006	0.9998	103.2 (1.9)	80.5 (9.7)	76.9 (1.2)
		Edible frog	0.006	0.9964	87.2 (12.0)	93.3 (5.8)	80.4 (1.7)
11	Chlordane-cis	Lamb	0.006	0.9921	76.0 (5.3)	77.4 (5.0)	75.1 (2.6)
		Duck meat	0.006	0.9993	77.9 (6.2)	76.7 (6.1)	74.5 (4.4)
		Quail egg	0.006	0.9993	84.8 (7.5)	84.3 (2.7)	75.0 (2.5)
		Mealworm	0.006	0.9943	112.4 (6.1)	71.7 (0.6)	73.6 (2.9)
		Edible frog	0.006	0.9998	86.4 (4.1)	85.7 (5.0)	82.3 (2.8)
12	Chlordane-trans	Lamb	0.006	0.9959	85.9 (11.7)	78.1 (5.2)	72.5 (3.2)
		Duck meat	0.006	0.9991	65.3 (3.6)	72.7 (2.4)	71.9 (2.6)
		Quail egg	0.006	0.9975	81.5 (4.5)	84.6 (3.7)	75.5 (2.6)
		Mealworm	0.006	0.9944	109.5 (1.2)	73.8 (5.0)	70.7 (1.2)
		Edible frog	0.006	0.9996	88.7 (8.3)	89.9 (3.4)	82.1 (1.8)
13	Oxychlordane	Lamb	0.006	0.9982	85.1 (8.0)	82.5 (3.9)	76.0 (2.4)
		Duck meat	0.006	0.9996	75.3 (3.5)	73.9 (4.3)	72.0 (1.7)
		Quail egg	0.006	0.9991	91.7 (12.2)	84.5 (12.8)	78.8 (1.7)
		Mealworm	0.006	0.9966	101.9 (13.7)	96.0 (9.5)	72.8 (2.4)
		Edible frog	0.006	0.9978	88.6 (6.8)	86.6 (9.2)	83.8 (1.3)
14	Chlorfenapyr	Lamb	0.006	0.9998	69.7 (6.5)	91.6 (13.8)	85.1 (8.9)
		Duck meat	0.006	0.9933	80.5 (14.0)	104.5 (4.7)	105.9 (2.9)
		Quail egg	0.006	0.9952	95.4 (12.5)	103.2 (6.6)	86.0 (14.3)
		Mealworm	0.006	0.9985	97.4 (11.7)	76.8 (4.2)	90.0 (8.2)
		Edible frog	0.006	0.9992	82.0 (9.1)	83.6 (11.6)	88.5 (5.4)
15	Chlorfenvinphos-E	Lamb	0.006	0.9971	105.6 (10.8)	96.6 (6.4)	93.5 (7.7)
		Duck meat	0.006	0.9987	104.9 (10.1)	101.8 (2.4)	100.1 (3.3)
		Quail egg	0.006	0.9996	101.9 (5.6)	89.9 (6.0)	82.0 (6.4)
		Mealworm	0.006	0.9994	83.2 (12.7)	73.6 (2.5)	86.0 (1.1)
		Edible frog	0.006	0.9949	74.8 (3.3)	82.3 (3.3)	79.2 (10.0)
16	Chlorfenvinphos-Z	Lamb	0.006	0.9994	101.5 (7.1)	96.8 (8.3)	81.5 (1.3)
		Duck meat	0.006	0.9968	77.1 (6.2)	84.2 (6.5)	95.5 (10.5)
		Quail egg	0.006	0.9997	95.2 (8.8)	95.4 (7.0)	82.2 (12.7)
		Mealworm	0.006	0.9995	84.2 (16.1)	78.7 (4.7)	84.2 (0.7)
		Edible frog	0.006	1.0000	76.1 (2.6)	79.8 (5.6)	75.8 (2.4)
17	Chlorobenzilate	Lamb	0.006	0.9972	97.5 (3.6)	96.3 (2.9)	91.5 (0.5)
		Duck meat	0.006	0.9998	90.8 (9.1)	96.6 (0.6)	90.6 (4.3)
		Quail egg	0.006	0.9955	91.2 (3.3)	91.5 (8.2)	92.2 (6.0)
		Mealworm	0.006	0.9991	112.0 (3.1)	72.3 (3.4)	80.1 (1.5)
		Edible frog	0.006	0.9996	83.9 (2.8)	88.9 (4.7)	84.4 (2.3)

No.	Analyte	Matrix	LOQ (mg/kg)	r^2	Recovery at 0.01 mg/kg (%, RSD %)	Recovery at 0.02 mg/kg (%, RSD %)	Recovery at 0.1 mg/kg (%, RSD %)
18	Chlorpropham	Lamb	0.006	0.9968	100.3 (4.7)	105.2 (7.1)	95.0 (8.0)
		Duck meat	0.006	0.9984	107.2 (4.0)	104.8 (5.4)	99.9 (0.9)
		Quail egg	0.006	0.9980	104.5 (3.1)	106.7 (11.0)	78.8 (4.7)
		Mealworm	0.006	0.9974	108.2 (7.8)	80.8 (12.4)	82.7 (1.2)
		Edible frog	0.006	0.9978	101.2 (5.0)	99.4 (10.8)	80.7 (3.3)
19	Chlorpyrifos	Lamb	0.006	0.9938	87.8 (14.0)	90.1 (6.1)	96.4 (1.3)
		Duck meat	0.006	0.9970	72.1 (12.6)	78.2 (5.3)	87.7 (7.8)
		Quail egg	0.006	0.9972	107.9 (5.8)	94.8 (10.2)	82.1 (10.5)
		Mealworm	0.006	0.9988	111.9 (6.5)	75.7 (1.8)	77.2 (1.6)
		Edible frog	0.006	0.9984	78.3 (9.4)	83.0 (6.6)	83.3 (6.0)
20	Chlorpyrifos-methyl	Lamb	0.006	0.9998	84.4 (2.5)	101.0 (3.7)	94.8 (5.1)
		Duck meat	0.006	0.9976	96.1 (8.9)	101.3 (4.9)	97.2 (3.8)
		Quail egg	0.006	0.9989	109.0 (3.8)	104.5 (8.4)	90.2 (1.5)
		Mealworm	0.006	0.9983	86.1 (7.5)	75.0 (0.7)	78.8 (3.8)
		Edible frog	0.006	0.9966	70.4 (3.5)	75.0 (3.0)	78.8 (3.4)
21-1	Cyfluthrin-1	Lamb	0.006	0.9998	76.5 (8.9)	90.4 (4.8)	87.6 (7.4)
		Duck meat	0.006	0.9991	92.0 (4.1)	87.2 (4.0)	97.7 (4.4)
		Quail egg	0.006	0.9985	78.4 (6.9)	84.2 (10.0)	87.9 (1.9)
		Mealworm	0.006	0.9994	107.1 (6.3)	79.7 (8.4)	76.7 (1.9)
		Edible frog	0.006	0.9999	78.1 (15.3)	77.5 (10.0)	87.3 (5.2)
21-2	Cyfluthrin-2	Lamb	0.006	0.9992	93.1 (4.6)	77.2 (5.1)	76.9 (6.3)
		Duck meat	0.006	0.9980	96.1 (7.6)	84.1 (4.8)	92.7 (6.2)
		Quail egg	0.006	0.9981	82.3 (4.2)	79.6 (4.9)	87.0 (2.1)
		Mealworm	0.006	0.9968	98.6 (12.3)	77.2 (5.6)	79.0 (1.8)
		Edible frog	0.006	0.9986	101.0 (6.9)	84.7 (12.5)	85.0 (9.9)
21-3	Cyfluthrin-3	Lamb	0.006	0.9990	79.8 (0.9)	82.4 (9.1)	76.4 (4.7)
		Duck meat	0.006	0.9996	92.5 (4.4)	94.9 (8.8)	89.4 (2.1)
		Quail egg	0.006	0.9997	96.6 (2.1)	84.1 (14.3)	86.3 (7.8)
		Mealworm	0.006	0.9993	77.2 (10.0)	76.2 (12.1)	78.1 (3.0)
		Edible frog	0.006	0.9992	77.7 (13.9)	83.1 (10.4)	76.6 (6.3)
21-4	Cyfluthrin-4	Lamb	0.006	0.9978	77.4 (6.1)	88.9 (11.1)	85.2 (7.3)
		Duck meat	0.006	0.9990	93.1 (2.8)	81.4 (3.4)	92.1 (8.8)
		Quail egg	0.006	0.9955	93.1 (7.7)	81.1 (8.8)	83.0 (11.2)
		Mealworm	0.006	0.9996	113.6 (1.7)	96.3 (17.9)	89.0 (6.1)
		Edible frog	0.006	0.9931	86.0 (12.8)	77.8 (6.8)	102.4 (5.2)
22	Cyhalothrin-γ	Lamb	0.006	0.9996	96.8 (1.4)	94.0 (2.5)	82.7 (0.7)
		Duck meat	0.006	0.9993	107.5 (2.5)	107.8 (5.3)	95.3 (3.3)
		Quail egg	0.006	0.9995	83.0 (1.6)	89.5 (4.7)	88.0 (1.5)
		Mealworm	0.006	0.9991	106.9 (1.6)	77.2 (1.8)	82.8 (1.1)
		Edible frog	0.006	0.9987	84.9 (5.3)	95.4 (6.6)	84.1 (2.2)
23	Cyhalothrin-λ	Lamb	0.006	0.9991	77.9 (5.2)	87.3 (2.7)	86.1 (2.7)
		Duck meat	0.006	0.9975	92.8 (13.9)	97.1 (7.7)	99.3 (2.1)
		Quail egg	0.006	0.9999	92.7 (6.9)	97.1 (1.6)	87.8 (6.2)
		Mealworm	0.006	0.9998	91.1 (2.8)	72.5 (0.7)	85.0 (3.3)
		Edible frog	0.006	0.9985	81.3 (6.3)	90.2 (4.1)	90.0 (3.6)

No.	Analyte	Matrix	LOQ (mg/kg)	r^2	Recovery at 0.01 mg/kg (%, RSD %)	Recovery at 0.02 mg/kg (%, RSD %)	Recovery at 0.1 mg/kg (%, RSD %)
24-1	Cypermethrin-1	Lamb	0.006	0.9995	92.8 (2.6)	88.7 (9.2)	86.1 (2.1)
		Duck meat	0.006	0.9997	84.0 (2.1)	86.1 (3.2)	91.0 (4.9)
		Quail egg	0.006	0.9962	85.4 (3.4)	91.9 (10.4)	80.8 (4.8)
		Mealworm	0.006	0.9997	95.1 (11.6)	78.2 (2.3)	74.3 (2.5)
		Edible frog	0.006	0.9970	102.5 (12.2)	90.2 (5.6)	89.4 (9.2)
24-2	Cypermethrin-2	Lamb	0.006	0.9999	93.3 (3.0)	90.9 (2.6)	83.9 (6.1)
		Duck meat	0.006	0.9988	102.8 (5.5)	100.7 (3.9)	99.2 (7.3)
		Quail egg	0.006	0.9990	84.2 (3.3)	77.9 (9.8)	89.6 (3.4)
		Mealworm	0.006	0.9985	113.8 (5.1)	78.6 (8.7)	111.0 (1.5)
		Edible frog	0.006	0.9996	70.0 (10.1)	75.0 (2.5)	76.0 (1.2)
24-3	Cypermethrin-3	Lamb	0.006	0.9979	105.8 (4.6)	88.2 (17.6)	82.5 (14.2)
		Duck meat	0.006	0.9982	105.9 (8.9)	97.9 (18.8)	101.1 (2.9)
		Quail egg	0.006	0.9990	86.5 (13.3)	83.9 (5.1)	81.2 (4.6)
		Mealworm	0.006	0.9974	83.1 (5.2)	88.7 (12.3)	89.1 (3.4)
		Edible frog	0.006	0.9968	84.4 (13.5)	102.3 (6.8)	104.4 (3.7)
24-4	Cypermethrin-4	Lamb	0.006	0.9996	84.5 (13.0)	90.3 (15.0)	77.5 (3.9)
		Duck meat	0.006	0.9904	90.7 (11.1)	95.3 (7.5)	96.4 (5.2)
		Quail egg	0.006	0.9993	91.3 (11.8)	92.3 (5.5)	80.3 (7.4)
		Mealworm	0.006	0.9952	89.5 (4.8)	75.6 (7.8)	89.1 (4.7)
		Edible frog	0.006	0.9978	96.1 (8.8)	83.8 (9.2)	78.0 (3.6)
25	Cyprodinil	Lamb	0.006	0.9922	82.1 (1.9)	83.8 (2.7)	83.6 (4.0)
		Duck meat	0.006	0.9927	91.0 (2.6)	81.8 (4.6)	88.7 (2.4)
		Quail egg	0.006	0.9945	83.6 (10.3)	97.8 (6.8)	79.2 (2.2)
		Mealworm	0.006	0.9997	107.2 (4.3)	91.0 (4.4)	82.4 (1.1)
		Edible frog	0.006	0.9978	74.2 (0.6)	79.5 (5.1)	78.6 (10.3)
26	DDT-p,p'	Lamb	0.006	0.9967	84.2 (9.9)	73.9 (1.7)	72.2 (2.4)
		Duck meat	0.006	0.9994	79.0 (0.8)	74.4 (3.3)	75.1 (0.6)
		Quail egg	0.006	0.9952	85.4 (9.2)	83.0 (9.9)	73.3 (3.0)
		Mealworm	0.006	0.9959	108.7 (14.6)	76.0 (5.6)	81.3 (1.4)
		Edible frog	0.006	0.9977	77.4 (7.9)	75.7 (2.1)	72.9 (3.2)
27	DDT-o,p'	Lamb	0.006	0.9967	84.1 (1.5)	82.4 (2.8)	75.0 (1.2)
		Duck meat	0.006	0.9994	73.7 (1.6)	72.3 (0.9)	72.7 (2.9)
		Quail egg	0.006	0.9994	93.9 (8.8)	97.5 (6.4)	80.1 (5.2)
		Mealworm	0.006	0.9994	108.3 (6.0)	70.6 (0.7)	73.0 (1.6)
		Edible frog	0.006	0.9992	72.9 (4.0)	78.1 (4.3)	79.4 (2.6)
28	DDE-p,p'	Lamb	0.006	0.9986	101.0 (2.1)	93.1 (1.3)	72.4 (0.8)
		Duck meat	0.006	0.9997	72.6 (3.4)	78.0 (2.6)	79.5 (1.4)
		Quail egg	0.006	0.9982	81.1 (1.8)	75.4 (7.9)	76.4 (6.2)
		Mealworm	0.006	0.9939	114.6 (3.9)	74.9 (2.2)	73.7 (1.4)
		Edible frog	0.006	0.9973	86.8 (2.0)	85.9 (4.2)	80.6 (2.0)
29	DDD-p,p'	Lamb	0.006	0.9976	89.1 (1.1)	81.1 (2.5)	82.1 (1.4)
		Duck meat	0.006	0.9970	78.9 (3.0)	78.2 (4.6)	72.4 (2.1)
		Quail egg	0.006	0.9982	86.0 (4.7)	87.1 (6.1)	72.4 (1.9)
		Mealworm	0.006	0.9997	100.6 (12.0)	76.8 (0.6)	72.6 (0.6)
		Edible frog	0.006	0.9994	75.2 (7.4)	84.1 (2.0)	75.3 (2.3)

No.	Analyte	Matrix	LOQ (mg/kg)	r^2	Recovery at 0.01 mg/kg (%, RSD %)	Recovery at 0.02 mg/kg (%, RSD %)	Recovery at 0.1 mg/kg (%, RSD %)
30-1	Deltamethrin-1	Lamb	0.006	0.9983	108.6 (9.5)	88.3 (11.4)	82.8 (6.4)
		Duck meat	0.006	0.9984	108.1 (5.9)	88.9 (11.6)	91.0 (5.4)
		Quail egg	0.006	0.9944	105.6 (9.5)	84.1 (7.7)	76.2 (6.5)
		Mealworm	0.006	0.9940	81.1 (10.6)	97.8 (1.4)	103.0 (4.8)
		Edible frog	0.006	0.9946	87.0 (18.2)	101.3 (11.6)	92.4 (4.4)
30-2	Deltamethrin-2	Lamb	0.006	0.9969	77.4 (3.6)	79.5 (3.4)	84.1 (2.2)
		Duck meat	0.006	0.9902	97.4 (6.8)	99.3 (2.6)	90.9 (6.1)
		Quail egg	0.006	0.9976	98.0 (15.7)	87.7 (8.0)	77.2 (3.9)
		Mealworm	0.006	0.9953	87.4 (2.6)	77.1 (7.9)	76.6 (2.5)
		Edible frog	0.006	0.9929	79.4 (6.2)	80.4 (8.6)	76.1 (6.7)
31	Dimethenamid	Lamb	0.006	0.978	90.2 (0.9)	93.4 (2.0)	98.6 (0.9)
		Duck meat	0.006	0.9996	98.6 (7.2)	102.8 (3.3)	97.7 (2.1)
		Quail egg	0.006	1.0000	102.7 (4.8)	103.8 (5.3)	97.3 (1.4)
		Mealworm	0.006	0.9992	98.6 (4.2)	72.7 (3.9)	81.2 (4.5)
		Edible frog	0.006	0.9989	87.5 (3.5)	90.1 (6.8)	82.6 (2.9)
32	Dimethipin	Lamb	0.006	0.9990	115.8 (3.2)	103.2 (5.2)	104.7 (2.1)
		Duck meat	0.006	0.9939	108.4 (9.0)	114.2 (5.5)	105.9 (8.8)
		Quail egg	0.01	0.9975	107.2 (8.9)	105.7 (10.0)	76.6 (5.7)
		Mealworm	0.006	0.9984	96.9 (4.5)	79.9 (7.2)	82.3 (10.8)
		Edible frog	0.01	0.9976	93.4 (5.6)	116.6 (1.5)	78.9 (3.5)
33	Diphenylamine	Lamb	0.006	0.9976	80.4 (2.7)	91.4 (1.8)	95.2 (2.5)
		Duck meat	0.006	0.9999	82.9 (6.9)	93.7 (6.1)	98.9 (2.1)
		Quail egg	0.006	0.9982	103.9 (4.5)	85.1 (4.5)	91.5 (17.5)
		Mealworm	0.006	0.9989	82.7 (12.3)	77.7 (2.3)	79.8 (2.8)
		Edible frog	0.006	0.9990	85.0 (12.7)	86.2 (11.6)	75.9 (1.5)
34	Endosulfan-α	Lamb	0.006	0.9965	74.9 (6.7)	80.4 (2.4)	83.5 (2.0)
		Duck meat	0.006	0.9998	83.8 (3.7)	80.8 (3.7)	73.4 (1.0)
		Quail egg	0.006	0.9993	78.5 (1.5)	86.0 (1.7)	84.5 (4.3)
		Mealworm	0.006	0.9995	110.3 (3.5)	73.8 (1.7)	73.3 (0.5)
		Edible frog	0.006	0.9997	75.5 (1.0)	83.8 (5.7)	84.6 (3.8)
35	Endosulfan-β	Lamb	0.006	0.9999	98.6 (6.8)	97.7 (4.1)	85.6 (3.3)
		Duck meat	0.006	0.9977	94.2 (14.8)	88.9 (8.7)	79.2 (3.8)
		Quail egg	0.006	0.9983	75.0 (9.5)	84.1 (8.9)	85.1 (10.4)
		Mealworm	0.006	0.9993	106.8 (6.2)	74.8 (4.6)	84.9 (8.4)
		Edible frog	0.006	0.9974	74.0 (3.8)	83.7 (12.6)	84.2 (5.5)
36	Endosulfan sulfate	Lamb	0.006	0.9998	97.9 (9.3)	91.0 (5.4)	80.5 (3.6)
		Duck meat	0.006	0.986	72.0 (3.7)	77.0 (7.4)	78.3 (5.9)
		Quail egg	0.006	0.9978	75.7 (2.8)	81.2 (1.5)	76.1 (2.4)
		Mealworm	0.006	0.9996	106.9 (5.3)	77.6 (1.3)	80.6 (1.1)
		Edible frog	0.006	0.9965	85.9 (5.3)	79.5 (8.7)	73.7 (2.0)
37	Endrin	Lamb	0.006	0.9989	98.1 (18.5)	91.1 (11.0)	84.0 (3.2)
		Duck meat	0.006	0.9993	81.8 (15.7)	77.6 (8.7)	75.0 (2.1)
		Quail egg	0.006	0.9950	84.5 (13.2)	85.7 (6.7)	90.4 (8.7)
		Mealworm	0.006	0.9984	102.7 (2.5)	89.0 (5.3)	75.7 (3.6)
		Edible frog	0.006	0.9993	87.6 (11.0)	101.5 (4.4)	83.5 (0.7)

No.	Analyte	Matrix	LOQ (mg/kg)	r^2	Recovery at 0.01 mg/kg (%, RSD %)	Recovery at 0.02 mg/kg (%, RSD %)	Recovery at 0.1 mg/kg (%, RSD %)
38	Endrin-δ-keto	Lamb	0.006	0.9994	83.9 (4.5)	89.8 (7.6)	93.6 (5.5)
		Duck meat	0.006	0.9998	97.7 (17.1)	87.9 (1.6)	86.8 (1.4)
		Quail egg	0.006	0.9983	111.8 (4.3)	88.4 (13.2)	93.9 (6.6)
		Mealworm	0.006	0.9996	95.3 (5.2)	82.4 (3.0)	79.3 (0.8)
		Edible frog	0.006	0.9998	88.6 (9.6)	77.5 (8.1)	85.4 (2.3)
39	EPN	Lamb	0.006	0.9996	84.9 (4.4)	85.5 (3.9)	86.1 (3.8)
		Duck meat	0.006	0.9943	90.9 (6.9)	96.6 (8.6)	103.4 (7.8)
		Quail egg	0.006	0.9997	84.1 (10.9)	86.9 (2.4)	91.8 (7.5)
		Mealworm	0.006	0.9996	84.6 (6.6)	79.3 (9.7)	84.5 (7.6)
		Edible frog	0.006	0.9994	86.5 (9.7)	87.1 (9.6)	72.2 (2.4)
40	Ethion	Lamb	0.006	0.9959	99.2 (8.2)	94.0 (2.5)	88.4 (5.4)
		Duck meat	0.006	0.9996	82.7 (4.8)	88.8 (2.1)	91.1 (3.7)
		Quail egg	0.006	0.9985	86.4 (13.6)	80.9 (3.2)	80.0 (8.3)
		Mealworm	0.006	0.9999	113.2 (5.2)	83.7 (6.5)	86.5 (2.4)
		Edible frog	0.006	0.9982	80.9 (12.5)	76.8 (12.5)	80.9 (6.2)
41	Ethoprophos	Lamb	0.006	0.9991	109.6 (8.3)	99.9 (4.7)	93.5 (1.9)
		Duck meat	0.006	0.9999	106.8 (13.7)	99.4 (3.8)	95.4 (5.0)
		Quail egg	0.006	0.9996	109.3 (3.2)	110.0 (1.3)	89.8 (4.7)
		Mealworm	0.006	0.9997	111.9 (2.7)	73.8 (1.9)	78.2 (5.6)
		Edible frog	0.006	0.9990	73.3 (2.3)	89.3 (5.1)	79.7 (6.6)
42	Etofenprox	Lamb	0.006	0.9992	74.6 (4.7)	78.8 (5.7)	73.2 (2.2)
		Duck meat	0.006	0.9992	80.8 (2.8)	77.7 (6.1)	79.3 (0.9)
		Quail egg	0.006	0.9995	77.6 (0.9)	76.9 (7.4)	72.1 (0.7)
		Mealworm	0.006	0.9996	96.9 (14.2)	100.2 (3.7)	85.4 (8.7)
		Edible frog	0.006	0.9986	65.8 (6.8)	79.9 (3.9)	77.9 (5.9)
43	Etoxazole	Lamb	0.006	0.9969	86.2 (2.3)	91.0 (6.0)	88.8 (1.0)
		Duck meat	0.006	0.9998	91.2 (7.4)	94.2 (3.0)	87.0 (1.7)
		Quail egg	0.006	0.9987	85.1 (5.8)	93.2 (5.7)	85.2 (2.6)
		Mealworm	0.006	0.9998	91.3 (0.8)	75.1 (5.6)	77.2 (1.6)
		Edible frog	0.006	0.9984	78.9 (4.9)	86.5 (4.9)	79.1 (2.6)
44	Fenitrothion	Lamb	0.006	0.9920	110.4 (6.3)	103.8 (1.2)	95.4 (3.5)
		Duck meat	0.006	0.9983	99.5 (17.1)	111.1 (4.8)	107.8 (4.5)
		Quail egg	0.01	0.9993	115.6 (4.7)	89.8 (6.5)	86.3 (13.6)
		Mealworm	0.006	0.9949	100.8 (14.1)	82.0 (3.5)	89.1 (8.0)
		Edible frog	0.006	0.9989	73.2 (4.0)	76.4 (7.3)	70.8 (6.4)
45	Fenoxanil	Lamb	0.006	0.9997	101.7 (10.9)	108.7 (5.1)	81.1 (2.5)
		Duck meat	0.006	0.9992	100.6 (10.7)	103.7 (5.6)	93.2 (4.0)
		Quail egg	0.006	0.9995	99.1 (8.1)	85.9 (9.0)	85.6 (6.6)
		Mealworm	0.006	0.9994	79.1 (8.2)	75.0 (4.9)	88.7 (3.2)
		Edible frog	0.006	0.9993	79.9 (7.0)	79.0 (8.5)	78.0 (3.3)
46	Fenpropathrin	Lamb	0.006	0.9977	102.5 (8.7)	95.3 (3.0)	94.2 (6.9)
		Duck meat	0.006	0.9999	101.5 (5.3)	102.1 (2.1)	90.8 (3.9)
		Quail egg	0.006	0.9979	79.1 (5.3)	89.8 (3.3)	84.0 (1.8)
		Mealworm	0.006	0.9981	95.8 (6.3)	73.4 (1.9)	80.8 (3.0)
		Edible frog	0.006	0.9999	75.6 (7.6)	80.8 (3.6)	81.9 (2.8)

No.	Analyte	Matrix	LOQ (mg/kg)	r^2	Recovery at 0.01 mg/kg (%, RSD %)	Recovery at 0.02 mg/kg (%, RSD %)	Recovery at 0.1 mg/kg (%, RSD %)
47	Fenthion	Lamb	0.006	0.9958	102.2 (11.3)	118.8 (12.4)	100.8 (6.3)
		Duck meat	0.006	0.9986	84.0 (14.1)	88.7 (18.2)	101.7 (6.4)
		Quail egg	0.006	0.9952	98.3 (10.8)	88.0 (13.3)	90.1 (10.8)
		Mealworm	0.006	0.9992	110.0 (8.5)	85.7 (8.8)	79.4 (9.9)
		Edible frog	0.006	0.9992	73.5 (1.5)	80.2 (11.0)	76.3 (3.0)
48-1	Fenvalerate/Esfenvalerate-1	Lamb	0.006	0.9992	79.7 (6.4)	83.5 (4.6)	86.8 (3.5)
		Duck meat	0.006	0.9964	93.7 (8.0)	93.0 (6.1)	94.5 (17.6)
		Quail egg	0.006	0.9989	78.9 (2.7)	85.9 (5.5)	79.7 (3.8)
		Mealworm	0.006	0.9978	108.0 (6.0)	81.4 (5.1)	118.8 (1.1)
		Edible frog	0.006	0.9999	78.0 (1.5)	74.1 (7.4)	78.7 (3.8)
48-2	Fenvalerate/Esfenvalerate-2	Lamb	0.006	0.9981	84.9 (6.6)	82.6 (3.1)	78.6 (4.8)
		Duck meat	0.006	0.9918	82.8 (17.6)	88.4 (13.1)	88.8 (8.5)
		Quail egg	0.006	0.9931	76.1 (5.5)	75.3 (6.2)	80.0 (0.4)
		Mealworm	0.006	0.9950	107.6 (9.1)	90.3 (10.1)	98.2 (3.4)
		Edible frog	0.006	0.9953	75.9 (10.4)	83.4 (3.7)	76.6 (2.8)
49	Flusilazole	Lamb	0.006	0.9997	94.1 (13.6)	90.9 (5.8)	87.0 (2.3)
		Duck meat	0.006	0.9931	107.4 (11.3)	107.4 (1.5)	92.5 (4.0)
		Quail egg	0.006	0.9999	87.0 (2.7)	87.7 (13.0)	87.2 (7.5)
		Mealworm	0.006	0.9984	88.8 (15.1)	71.9 (2.6)	84.0 (6.3)
		Edible frog	0.006	0.9920	84.6 (3.9)	80.1 (10.8)	76.0 (5.6)
50	Heptachlor	Lamb	0.006	0.9926	91.3 (4.1)	86.4 (3.4)	81.7 (4.5)
		Duck meat	0.006	0.9976	73.6 (3.1)	71.6 (1.5)	67.3 (3.4)
		Quail egg	0.006	0.9996	93.3 (4.8)	84.8 (8.5)	73.1 (2.9)
		Mealworm	0.006	0.9980	110.2 (7.8)	76.8 (1.1)	70.8 (0.4)
		Edible frog	0.006	0.9998	78.1 (7.4)	78.0 (5.5)	78.2 (2.7)
51	Isofenphos	Lamb	0.006	0.9951	97.4 (7.1)	96.7 (7.5)	96.4 (5.7)
		Duck meat	0.006	0.9997	96.9 (6.3)	98.0 (4.6)	95.9 (3.6)
		Quail egg	0.006	0.993	108.4 (1.2)	109.3 (9.3)	92.9 (6.4)
		Mealworm	0.006	0.9997	81.1 (10.2)	73.8 (3.7)	82.5 (2.0)
		Edible frog	0.006	0.9999	83.7 (5.0)	87.3 (2.8)	82.1 (3.6)
52	Mecarbam	Lamb	0.006	0.9956	103.5 (12.0)	81.3 (7.5)	76.1 (1.5)
		Duck meat	0.006	0.9948	112.2 (3.1)	101.1 (11.5)	101.9 (6.7)
		Quail egg	0.006	0.9992	84.5 (3.3)	101.9 (4.7)	76.5 (6.2)
		Mealworm	0.006	0.9972	102.2 (9.0)	96.5 (13.4)	106.9 (9.4)
		Edible frog	0.006	0.9993	83.2 (2.8)	83.4 (12.7)	79.3 (5.0)
53	Methacrifos	Lamb	0.006	0.9986	102.8 (3.5)	96.9 (2.2)	96.4 (2.3)
		Duck meat	0.006	0.9996	102.1 (4.7)	102.9 (2.9)	101.7 (6.0)
		Quail egg	0.006	0.9981	103.7 (6.4)	92.4 (6.4)	94.3 (2.8)
		Mealworm	0.006	0.9996	96.8 (4.4)	75.0 (3.5)	83.5 (5.2)
		Edible frog	0.006	0.9972	79.0 (10.8)	81.9 (7.6)	78.6 (2.9)
54	Methidathion	Lamb	0.006	0.9993	97.6 (4.5)	95.5 (5.2)	81.6 (4.0)
		Duck meat	0.006	0.9903	112.1 (1.9)	101.6 (4.0)	102.3 (6.3)
		Quail egg	0.006	0.9997	76.3 (6.0)	99.0 (11.9)	89.3 (6.6)
		Mealworm	0.006	0.9994	101.9 (5.7)	89.6 (7.1)	89.7 (1.5)
		Edible frog	0.006	0.9970	65.0 (5.3)	73.1 (3.5)	80.5 (4.6)

No.	Analyte	Matrix	LOQ (mg/kg)	r^2	Recovery at 0.01 mg/kg (%, RSD %)	Recovery at 0.02 mg/kg (%, RSD %)	Recovery at 0.1 mg/kg (%, RSD %)
55	Parathion	Lamb	0.006	0.994	113.3 (4.1)	111.0 (2.5)	98.7 (4.8)
		Duck meat	0.006	0.9950	85.1 (7.8)	91.6 (10.3)	98.0 (9.0)
		Quail egg	0.006	0.9990	112.8 (8.6)	107.7 (5.3)	96.6 (7.5)
		Mealworm	0.006	0.9965	97.2 (14.0)	78.0 (10.9)	101.6 (6.1)
		Edible frog	0.006	0.9964	75.0 (4.8)	74.2 (3.4)	76.0 (5.4)
56	Penthiopyrad	Lamb	0.006	0.9997	95.2 (11.6)	96.0 (6.6)	77.6 (6.8)
		Duck meat	0.006	0.9940	93.0 (2.1)	96.8 (3.4)	100.7 (2.4)
		Quail egg	0.006	0.9922	113.2 (0.7)	92.2 (11.2)	90.6 (4.3)
		Mealworm	0.006	0.9993	100.6 (10.6)	72.9 (5.2)	93.4 (1.6)
		Edible frog	0.006	0.9984	75.7 (2.1)	83.4 (9.0)	82.5 (10.4)
57	Permethrin-cis	Lamb	0.006	0.9998	75.2 (2.7)	74.4 (1.3)	73.9 (2.7)
		Duck meat	0.006	0.9999	84.0 (9.3)	76.7 (3.0)	75.1 (4.2)
		Quail egg	0.006	0.9990	78.0 (3.3)	77.8 (3.3)	72.0 (4.0)
		Mealworm	0.006	0.9981	89.8 (8.6)	79.9 (12.1)	97.0 (2.3)
		Edible frog	0.006	0.9992	74.3 (7.4)	83.3 (1.3)	75.7 (2.6)
58	Permethrin-trans	Lamb	0.006	0.9994	76.6 (4.1)	84.6 (7.8)	79.5 (1.2)
		Duck meat	0.006	0.9965	67.3 (3.8)	81.2 (1.6)	79.9 (4.1)
		Quail egg	0.006	0.9965	80.9 (5.6)	82.0 (6.7)	79.6 (3.9)
		Mealworm	0.006	0.9988	83.0 (5.7)	84.0 (8.1)	78.2 (3.1)
		Edible frog	0.006	0.9998	77.7 (3.6)	85.8 (13.2)	76.4 (5.5)
59	Phenthoate	Lamb	0.006	0.9969	104.1 (9.7)	83.2 (5.4)	75.1 (3.2)
		Duck meat	0.006	0.9994	113.1 (1.2)	101.6 (13.9)	93.3 (5.2)
		Quail egg	0.006	0.9979	93.6 (11.9)	85.2 (1.1)	86.5 (4.2)
		Mealworm	0.006	0.9991	93.4 (18.6)	86.6 (9.8)	81.2 (3.4)
		Edible frog	0.006	0.9988	77.6 (7.0)	81.2 (8.1)	81.9 (4.8)
60	Phosmet	Lamb	0.04	0.9917	-	-	87.6 (5.8)
		Duck meat	0.02	0.9914	-	107.2 (4.5)	111.3 (1.9)
		Quail egg	0.04	0.9981	-	-	73.1 (2.2)
		Mealworm	0.006	0.9939	93.9 (4.9)	92.4 (8.1)	90.1 (2.8)
		Edible frog	0.04	0.9959	-	-	98.6 (4.4)
61	Phthalide (Fthalide)	Lamb	0.006	0.9912	111.5 (5.2)	114.3 (2.7)	104.7 (6.8)
		Duck meat	0.006	0.9989	91.2 (8.9)	94.6 (3.1)	96.8 (8.4)
		Quail egg	0.006	0.9992	74.6 (3.7)	77.8 (5.1)	78.7 (0.6)
		Mealworm	0.006	0.9992	87.9 (2.6)	77.7 (10.6)	75.2 (4.3)
		Edible frog	0.006	0.9957	73.5 (4.2)	83.3 (5.4)	71.4 (1.8)
62	Pirimicarb	Lamb	0.006	0.9981	98.0 (3.5)	96.7 (3.8)	93.3 (4.6)
		Duck meat	0.006	0.9991	86.8 (8.5)	92.9 (8.4)	96.5 (7.0)
		Quail egg	0.006	0.9988	91.2 (8.8)	94.3 (3.7)	94.5 (5.4)
		Mealworm	0.006	0.9998	85.3 (13.9)	74.8 (4.5)	85.2 (2.5)
		Edible frog	0.006	0.9903	74.6 (3.3)	78.7 (2.5)	81.6 (6.8)
63-1	Propiconazole-1	Lamb	0.006	0.9998	88.8 (7.0)	103.5 (1.4)	94.6 (3.5)
		Duck meat	0.006	0.9999	105.3 (8.8)	97.2 (0.8)	91.5 (2.5)
		Quail egg	0.006	0.9972	105.4 (6.7)	96.0 (1.3)	89.0 (0.9)
		Mealworm	0.006	0.9992	108.9 (6.2)	77.8 (2.6)	81.6 (1.0)
		Edible frog	0.006	0.9980	79.9 (6.6)	77.6 (9.1)	79.1 (3.9)

No.	Analyte	Matrix	LOQ (mg/kg)	r^2	Recovery at 0.01 mg/kg (%, RSD %)	Recovery at 0.02 mg/kg (%, RSD %)	Recovery at 0.1 mg/kg (%, RSD %)
63-2	Propiconazole-2	Lamb	0.006	0.9972	101.9 (2.9)	102.5 (3.5)	93.4 (2.9)
		Duck meat	0.006	0.9963	97.2 (7.4)	101.8 (10.3)	95.0 (6.7)
		Quail egg	0.006	0.9988	96.6 (8.4)	87.9 (3.6)	89.3 (5.9)
		Mealworm	0.006	0.9973	111.8 (5.4)	82.4 (6.9)	82.1 (3.4)
		Edible frog	0.006	0.9995	83.7 (6.1)	82.7 (8.0)	84.6 (4.4)
64	Pyridaben	Lamb	0.006	0.9998	68.2 (2.5)	83.1 (1.5)	81.4 (4.5)
		Duck meat	0.006	0.9998	79.5 (1.9)	79.5 (3.3)	78.8 (4.4)
		Quail egg	0.006	0.9996	73.7 (5.5)	78.0 (4.6)	74.0 (1.0)
		Mealworm	0.006	0.9997	106.8 (6.4)	70.9 (1.1)	72.6 (2.4)
		Edible frog	0.006	0.9962	83.5 (2.6)	82.5 (2.5)	74.1 (2.2)
65	Quinoxyfen	Lamb	0.006	0.9996	76.4 (1.2)	76.0 (2.1)	74.9 (0.7)
		Duck meat	0.006	0.9996	69.2 (6.8)	80.3 (5.8)	82.6 (1.7)
		Quail egg	0.006	0.9946	78.4 (9.9)	83.6 (6.0)	80.0 (1.5)
		Mealworm	0.006	0.9991	109.0 (1.8)	74.3 (1.5)	77.4 (5.8)
		Edible frog	0.006	0.9987	67.7 (3.5)	76.6 (5.4)	78.4 (4.2)
66	Quintozene	Lamb	0.006	0.9930	89.3 (6.9)	89.0 (1.4)	80.9 (4.7)
		Duck meat	0.006	0.9997	80.1 (1.2)	74.8 (1.8)	72.7 (2.5)
		Quail egg	0.006	0.9956	81.9 (3.0)	85.0 (8.0)	80.3 (1.3)
		Mealworm	0.006	1.0000	106.7 (5.0)	73.3 (1.7)	73.6 (3.4)
		Edible frog	0.006	0.9989	65.8 (4.5)	78.4 (6.1)	79.5 (6.3)
67	Pentachloroaniline	Lamb	0.006	0.9984	105.0 (4.9)	99.9 (4.5)	93.2 (3.4)
		Duck meat	0.006	0.9993	77.9 (8.7)	77.3 (9.4)	74.4 (5.3)
		Quail egg	0.006	0.9961	75.2 (6.4)	79.4 (12.7)	74.2 (2.4)
		Mealworm	0.006	0.9966	113.2 (2.3)	74.6 (6.5)	72.2 (1.5)
		Edible frog	0.006	0.9958	71.6 (0.4)	79.8 (0.8)	82.4 (5.2)
68	Spirodiclofen	Lamb	0.006	0.9999	91.7 (4.0)	89.3 (12.2)	90.5 (6.8)
		Duck meat	0.006	0.9976	69.5 (1.5)	78.6 (6.2)	82.4 (0.8)
		Quail egg	0.006	0.9993	83.2 (15.2)	78.4 (7.9)	75.7 (2.6)
		Mealworm	0.006	0.9995	112.9 (1.7)	82.8 (7.0)	70.8 (2.1)
		Edible frog	0.006	0.9991	85.3 (6.2)	78.9 (7.3)	71.1 (1.2)
69	Terbufos	Lamb	0.006	0.9962	91.3 (6.0)	95.6 (2.6)	94.5 (2.5)
		Duck meat	0.006	0.9999	97.9 (8.7)	93.4 (5.2)	89.6 (3.4)
		Quail egg	0.006	0.9993	108.4 (8.0)	102.3 (7.2)	89.8 (2.8)
		Mealworm	0.006	0.9994	99.6 (2.8)	71.8 (2.3)	76.7 (2.0)
		Edible frog	0.006	0.9993	78.4 (6.8)	81.4 (5.8)	84.3 (2.9)
70	Thifluzamide	Lamb	0.006	0.9997	111.7 (3.9)	101.8 (2.6)	89.3 (5.1)
		Duck meat	0.006	0.9979	97.1 (5.9)	107.7 (7.9)	103.8 (2.6)
		Quail egg	0.006	0.9974	88.4 (7.1)	92.2 (2.3)	86.4 (3.9)
		Mealworm	0.006	0.9988	83.0 (9.5)	72.1 (3.6)	85.4 (2.8)
		Edible frog	0.006	0.9946	74.1 (2.2)	77.8 (6.2)	73.2 (2.0)
71	Triadimefon	Lamb	0.006	0.9994	73.4 (4.7)	83.2 (0.4)	89.2 (4.7)
		Duck meat	0.006	0.9993	90.2 (16.1)	92.0 (12.5)	104.4 (5.8)
		Quail egg	0.006	0.9988	75.5 (4.8)	100.4 (12.5)	83.1 (3.3)
		Mealworm	0.006	0.9987	96.4 (9.0)	77.0 (10.0)	87.9 (3.0)
		Edible frog	0.006	0.9967	85.6 (16.0)	86.0 (6.4)	78.2 (2.1)

No.	Analyte	Matrix	LOQ (mg/kg)	r^2	Recovery at 0.01 mg/kg (%, RSD %)	Recovery at 0.02 mg/kg (%, RSD %)	Recovery at 0.1 mg/kg (%, RSD %)
72	Triadimenol	Lamb	0.01	0.9950	108.9 (3.1)	84.2 (4.7)	76.9 (1.1)
		Duck meat	0.02	0.9995	-	91.9 (5.8)	103.8 (5.2)
		Quail egg	0.02	0.9984	-	100.8 (4.2)	83.6 (4.7)
		Mealworm	0.006	0.9977	94.6 (13.6)	77.0 (11.5)	83.4 (3.1)
		Edible frog	0.006	0.9963	98.1 (4.5)	111.6 (2.3)	85.3 (0.3)
73	Triazophos	Lamb	0.02	0.9985	-	106.1 (3.8)	79.1 (4.6)
		Duck meat	0.01	0.9978	92.8 (11.4)	109.1 (4.3)	108.1 (7.8)
		Quail egg	0.04	0.9977	-	-	83.1 (6.6)
		Mealworm	0.006	0.9990	107.5 (8.8)	96.6 (12.8)	93.9 (2.7)
		Edible frog	0.01	0.9962	83.7 (13.2)	82.3 (8.8)	81.3 (5.8)
74	Vinclozolin	Lamb	0.006	0.9991	89.7 (8.6)	97.9 (7.5)	97.4 (5.0)
		Duck meat	0.006	0.9973	102.2 (8.8)	103.5 (5.7)	100.1 (12.8)
		Quail egg	0.006	0.9947	103.7 (9.6)	109.1 (12.5)	95.8 (8.7)
		Mealworm	0.006	0.9998	94.7 (8.2)	76.0 (3.5)	81.7 (9.5)
		Edible frog	0.006	0.9948	75.4 (2.8)	77.2 (8.6)	81.4 (6.9)
75	3,5-dichloroaniline	Lamb	0.006	0.9981	74.3 (10.9)	90.1 (3.5)	102.8 (0.7)
		Duck meat	0.006	0.9989	85.2 (4.6)	95.1 (1.0)	98.2 (6.3)
		Quail egg	0.006	0.9991	98.4 (5.6)	103.1 (4.9)	92.7 (0.9)
		Mealworm	0.006	0.9994	89.6 (14.9)	81.8 (11.3)	78.0 (8.6)
		Edible frog	0.006	0.9922	76.8 (4.1)	89.4 (8.4)	78.4 (4.2)

[1] -, not determined or not available in the final report.

Table S3. Commodity-specific intake values, surrogate assignments, and LOQ assignments used in the dietary exposure assessment.

Commodity	Monitoring samples (*n*)	IR_{mean} [1] (g/day)	$IR_{extreme}$ [1] (g/day)	Intake–value assignment [2]	Surrogate commodity	LOQ assignment [3]
Duck meat	15	3.716	647.43	Direct	—	Duck meat
Goat meat	15	0.003	45.945	Direct	—	Lamb meat
Lamb meat	10	0.01	92.127	Direct	—	Lamb meat
Horse meat	10	0.01	92.127	Surrogate	Lamb meat	Lamb meat
Quail egg	10	0.647	21.053	Direct	—	Quail egg
Deer meat	5	0.00005	0.00005	Default	—	Lamb meat
Rabbit meat	5	0.00005	0.00005	Default	—	Lamb meat
Turkey meat	5	0.029	524.39	Direct	—	Duck meat
Goose meat	5	3.716	647.43	Surrogate	Duck meat	Duck meat
Edible frog	2	0.00005	0.00005	Default	—	Edible frog
Edible snail	1	0.00005	0.00005	Default	—	0.01 mg/kg
Edible insect	4	0.00005	0.00005	Default	—	Mealworm
Soft–shelled turtle	3	0.00005	0.00005	Default	—	Edible frog

[1] IR_{mean} denotes the population-mean daily intake, including non-consumers, or its assigned substitute; $IR_{extreme}$ denotes the intake input used in Eqs. (1) and (4). Consumers-only 97.5th-percentile intakes were used where available, whereas consumers-only mean intakes were substituted for goat meat, lamb meat, and quail eggs. The lamb-meat value was also applied to horse meat.

[2] Lamb meat and duck meat were used as intake surrogates for horse meat and goose meat, respectively. For commodities lacking intake data and a suitable surrogate, a common default intake of 0.00005 g/day was assigned to both IR_{mean} and $IR_{extreme}$.

[3] For the UB scenario, analyte–specific LOQs from the validated matrices listed in this column were applied to the corresponding commodities. For edible snail, no comparable validated matrix was available; therefore, an LOQ of 0.01 mg/kg was assigned.

Table S4. LC–MS/MS multiple reaction monitoring (MRM) parameters and retention times for the target pesticide analytes.

No.	Analyte	Ionization mode	Retention time (min)	Quantifier transition		Qualifier transition	
				Precursor > product (m/z)	CE [1] (eV)	Precursor > product (m/z)	CE (eV)
1	Abamectin	Positive	15.67	895.45 > 751.30	–48	895.45 > 151.10	–65
2	Acephate	Positive	3.01	184.10 > 143.00	–9	184.10 > 95.00	–23
3	Acetamiprid	Positive	3.72	223.10 > 126.00	–20	223.10 > 56.10	–15
4	Acetamiprid-N-desmethyl (IM-2-1)	Positive	3.70	209.10 > 126.10	–17	209.10 > 99.10	–35
5	Afidopyropen	Positive	8.34	594.25 > 148.00	–54	594.25 > 202.15	–35
6	Alachlor	Positive	9.28	270.20 > 238.10	–9	270.20 > 162.20	–21
7	Aldicarb	Positive	4.40	213.10 > 89.15	–17	213.10 > 116.10	–12
8	Aldicarb-sulfone	Positive	3.14	240.15 > 223.10	–8	240.15 > 86.20	–22
9	Aldicarb-sulfoxide	Positive	3.08	207.15 > 132.20	–7	207.15 > 41.10	–41
10	Ametoctradin	Positive	11.63	276.30 > 149.10	–36	276.30 > 176.10	–36
11	Azinphos-methyl	Positive	6.98	318.05 > 125.10	–21	318.05 > 260.90	–8
12	Azoxystrobin	Positive	7.43	404.10 > 372.10	–15	404.10 > 329.10	–30
13	Bendiocarb	Positive	5.01	224.10 > 109.10	–18	224.10 > 81.10	–33
14	Bentazone	Negative	4.64	239.20 > 132.15	27	239.20 > 133.05	26
15	Benzovindiflupyr	Positive	10.42	398.05 > 342.15	–19	398.05 > 377.95	–16
16	Bifenazate	Positive	8.72	301.15 > 170.15	–20	301.15 > 198.10	–11
17	Bifenazate-diazene	Positive	11.84	299.15 > 196.10	–30	299.15 > 213.15	–12
18	Bioresmethrin	Positive	15.64	339.20 > 128.10	–46	339.20 > 171.10	–18
19	Bixafen	Positive	9.87	414.10 > 394.10	–15	414.10 > 266.00	–21
20	Boscalid	Positive	7.87	343.10 > 307.10	–18	343.10 > 140.00	–21
21	Carbaryl	Positive	5.42	202.10 > 145.10	–10	202.10 > 127.10	–27
22	Carbendazim	Positive	3.45	192.10 > 160.10	–18	192.10 > 132.10	–30
23	Carbofuran	Positive	5.06	222.10 > 123.10	–21	222.10 > 165.10	–10
24	Carbofuran-3-hydroxy	Positive	3.68	238.15 > 163.00	–15	238.15 > 181.20	–11
25	Carpropamide	Positive	10.44	333.60 > 139.00	–21	333.60 > 103.05	–42
26	Chlorantraniliprole	Positive	6.86	484.00 > 112.10	–60	484.00 > 286.10	–13
27	Chromafenozide	Positive	8.97	394.80 > 175.10	–19	394.80 > 147.00	–44
28	Clofentezine	Positive	11.27	303.00 > 137.95	–15	303.00 > 102.10	–35
29	Clothianidin	Positive	3.58	250.00 > 169.00	–13	250.00 > 131.95	–18
30	Coumaphos	Positive	10.62	363.05 > 227.00	–25	363.05 > 307.00	–18
31	Coumaphos-oxon	Positive	7.12	347.10 > 291.00	–19	347.10 > 210.95	–31
32	Cyanazine	Positive	4.70	240.80 > 214.05	–17	240.80 > 104.00	–30
33	Cyantraniliprole	Negative	5.41	473.00 > 204.20	12	473.00 > 202.05	16
34	Cyclaniliprole	Positive	9.62	601.95 > 112.00	–75	601.95 > 283.90	–18
35	Cyproconazole	Positive	8.26	292.10 > 70.10	–23	292.10 > 125.10	–31
36	Cyromazine	Positive	2.87	167.10 > 85.15	–19	167.10 > 68.15	–31
37	Diazinon	Positive	10.68	305.00 > 169.00	–25	305.00 > 153.00	–25
38	Dichlorvos	Positive	5.00	221.00 > 109.00	–17	221.00 > 78.90	–27
39	Diflubenzuron	Positive	9.55	311.05 > 141.00	–32	311.05 > 158.05	–16
40	Dimethoate	Positive	3.77	230.00 > 198.90	–11	230.00 > 124.95	–22
41	Dimethomorph-E	Positive	7.69	388.00 > 300.90	–25	388.00 > 164.95	–35
42	Dimethomorph-Z	Positive	8.20	388.00 > 300.90	–25	388.00 > 164.95	–35
43	Dinotefuran	Positive	3.10	203.15 > 129.10	–12	203.15 > 114.15	–12
44	Disulfoton	Positive	3.79	275.10 > 61.20	–32	275.10 > 29.05	–51
45	Disulfoton-sulfone	Positive	5.96	307.00 > 97.05	–30	307.00 > 125.05	–16
46	Disulfoton-sulfoxide	Positive	5.86	290.90 > 185.00	–14	290.90 > 213.00	–10
47	Demeton-S	Positive	7.20	259.05 > 89.10	–15	259.05 > 61.15	–34
48	Demeton-S-sulfone	Positive	3.85	291.05 > 235.05	–16	291.05 > 263.00	–13
49	Demeton-S-sulfoxide	Positive	3.79	274.90 > 141.00	–21	274.90 > 197.05	–12
50	Edifenphos	Positive	10.35	311.00 > 109.00	–40	311.00 > 111.00	–25

No.	Analyte	Ionization mode	Retention time (min)	Quantifier transition		Qualifier transition	
				Precursor > product (m/z)	CE [1] (eV)	Precursor > product (m/z)	CE (eV)
51	Epoxiconazole	Positive	9.24	330.00 > 121.00	–21	330.00 > 101.05	–49
52	Ethiofencarb	Positive	5.67	225.60 > 107.10	–16	225.60 > 77.00	–45
53	Ethiprole	Positive	7.64	397.00 > 350.90	–22	397.00 > 255.00	–37
54	Etrimfos	Positive	10.40	292.60 > 125.00	–25	292.60 > 265.00	–17
55	Famoxadone	Positive	10.69	392.20 > 331.15	–11	392.20 > 238.00	–20
56	Fenamidone	Positive	7.71	311.70 > 92.10	–24	311.70 > 236.05	–15
57	Fenarimol	Positive	9.02	331.00 > 268.10	–25	331.00 > 139.00	–37
58	Fenbuconazole	Positive	9.43	336.90 > 125.00	–31	336.90 > 70.05	–21
59	Fenhexamid	Positive	8.89	302.00 > 97.15	–24	302.00 > 55.05	–41
60	Fenobucarb	Positive	7.36	208.20 > 95.10	–17	208.20 > 41.05	–36
61	Fenpyroximate	Positive	14.62	421.60 > 366.05	–16	421.60 > 138.00	–31
62	Fensulfothion	Positive	6.37	309.00 > 281.00	–15	309.00 > 252.95	–20
63	Ferimzone-E	Positive	6.40	255.15 > 132.20	–20	255.15 > 91.10	–31
64	Ferimzone-Z	Positive	7.18	255.15 > 132.20	–20	255.15 > 91.10	–31
65	Fipronil	Negative	9.68	435.00 > 329.95	15	435.00 > 249.95	26
66	Fipronil-sulfone	Negative	10.51	450.85 > 414.80	19	450.85 > 282.10	25
67	Flubendiamide	Negative	10.13	680.70 > 254.10	26	680.70 > 274.10	15
68	Fludioxonil	Negative	7.98	247.00 > 180.00	27	247.00 > 126.00	28
69	Flufenoxuron	Positive	14.06	488.80 > 158.00	–20	488.80 > 140.95	–46
70	Flumethrin	Positive	16.29	527.15 > 266.95	–16	527.15 > 510.05	–11
71	Flumioxazin	Positive	6.88	355.15 > 299.00	–28	355.15 > 327.05	–19
72	Fluopicolide	Positive	8.26	382.50 > 172.90	–23	382.50 > 144.95	–48
73	Fluralaner	Positive	12.33	556.10 > 400.00	–23	556.10 > 160.00	–46
74	Flutolanil	Positive	8.13	323.60 > 242.00	–26	323.60 > 262.00	–19
75	Flutriafol	Positive	6.06	301.60 > 70.05	–23	301.60 > 123.10	–28
76	Fluxapyroxad	Positive	8.21	382.15 > 342.10	–22	382.15 > 362.05	–14
77	Imazalil	Positive	5.60	296.60 > 158.90	–23	296.60 > 200.90	–18
78	Indoxacarb	Positive	11.84	528.10 > 203.05	–39	528.10 > 150.00	–24
79	Isofetamid	Positive	9.00	360.20 > 125.00	–28	360.20 > 210.20	–10
80	Isoprocarb	Positive	6.17	194.20 > 95.15	–15	194.20 > 77.10	–36
81	Isoprothiolane	Positive	8.29	291.10 > 188.95	–21	291.10 > 231.00	–12
82	Isopyrazam	Positive	11.63	360.20 > 244.10	–23	360.20 > 320.10	–20
83	Ivermectin	Positive	17.05	897.50 > 753.40	–46	897.50 > 329.20	–54
84	Kresoxim-methyl	Positive	10.08	314.15 > 222.05	–17	314.15 > 267.15	–9
85	Lufenuron	Positive	13.40	511.00 > 141.10	–40	511.00 > 158.15	–20
86	Malathion	Positive	8.18	330.80 > 99.00	–23	330.80 > 284.90	–8
87	Metaflumizone-E	Positive	13.10	507.00 > 178.15	–27	507.00 > 287.05	–27
88	Metaflumizone-Z	Positive	12.00	507.00 > 178.15	–27	507.00 > 287.05	–27
89	Methamidophos	Positive	2.92	142.00 > 94.00	–18	142.00 > 124.95	–17
90	Methiocarb	Positive	7.70	225.80 > 121.05	–20	225.80 > 169.00	–10
91	Methomyl	Positive	3.30	163.00 > 88.00	–10	163.00 > 106.05	–11
92	Methoprene [2]	Positive	15.80	279.30 > 191.25	–10	279.30 > 121.25	–25
93	Methoxyfenozide	Positive	8.40	369.00 > 149.00	–21	369.00 > 313.10	–8
94	Metrafenone	Positive	11.20	408.90 > 209.00	–15	408.90 > 226.90	–22
95	Monocrotophos	Positive	3.33	223.60 > 126.95	–15	223.60 > 192.95	–8
96	Myclobutanil	Positive	8.40	289.15 > 125.10	–32	289.15 > 70.10	–23
97	Novaluron	Positive	12.31	493.05 > 141.00	–45	493.05 > 158.05	–26
98	Orysastrobin	Positive	8.30	392.10 > 204.95	–15	392.10 > 116.05	–28
99	Oxamyl	Positive	3.17	236.80 > 72.10	–25	236.80 > 90.05	–8
100	Oxathiapiprolin	Positive	8.27	540.15 > 500.05	–25	540.15 > 522.15	–27
101	Penconazole	Positive	10.17	284.00 > 158.95	–30	284.00 > 70.05	–16
102	Pencycuron	Positive	11.34	329.30 > 125.10	–24	329.30 > 218.20	–17
103	Pendimethalin	Positive	13.71	282.10 > 212.00	–12	282.10 > 194.00	–18

No.	Analyte	Ionization mode	Retention time (min)	Quantifier transition		Qualifier transition	
				Precursor > product (m/z)	CE [1] (eV)	Precursor > product (m/z)	CE (eV)
104	Phorate	Positive	11.05	261.00 > 75.15	−10	261.00 > 46.90	−36
105	Phorate-sulfone	Positive	6.03	293.00 > 115.00	−16	293.00 > 171.00	−20
106	Phorate-sulfoxide	Positive	5.88	277.00 > 143.00	−16	277.00 > 199.00	−20
107	Phorate-oxon	Positive	6.82	244.90 > 75.05	−12	244.90 > 47.00	−32
108	Phorate-oxon-sulfone	Positive	3.86	276.55 > 111.05	−24	276.55 > 127.10	−17
109	Phorate-oxon-sulfoxide	Positive	3.71	261.05 > 110.85	−25	261.05 > 153.00	−14
110	Phosalone	Positive	11.01	367.70 > 181.95	−17	367.70 > 111.00	−40
111	Phoxim	Positive	11.01	298.50 > 77.00	−30	298.50 > 128.95	−11
112	Picoxystrobin	Positive	9.90	367.90 > 145.00	−21	367.90 > 205.05	−9
113	Piperonyl butoxide	Positive	13.15	356.25 > 177.10	−15	356.25 > 119.15	−34
114	Pirimiphos-methyl	Positive	11.01	305.70 > 108.00	−31	305.70 > 67.00	−43
115	Profenofos	Positive	12.46	374.80 > 304.75	−18	374.80 > 346.75	−13
116	Propamocarb	Positive	3.04	188.70 > 102.10	−20	188.70 > 74.00	−25
117	Propargite	Positive	14.07	368.10 > 231.25	−12	368.10 > 175.25	−18
118	Propoxur	Positive	5.00	209.80 > 111.00	−14	209.80 > 93.00	−25
119	Pydiflumetofen	Positive	11.39	425.70 > 192.95	−41	425.70 > 406.10	−14
120	Pyraclostrobin	Positive	10.95	388.10 > 163.20	−24	388.10 > 194.20	−13
121	Pyrimethanil	Positive	7.41	200.15 > 107.10	−24	200.15 > 82.15	−26
122	Pyrimethanil-4-hydroxy	Positive	4.05	216.15 > 107.10	−26	216.15 > 109.05	−30
123	Pyrimethanil-5-hydroxy	Positive	5.66	216.15 > 77.15	−47	216.15 > 143.00	−10
124	Pyriproxyfen	Positive	13.38	322.15 > 96.15	−17	322.15 > 78.15	−60
125	Saflufenacil	Positive	7.03	500.80 > 197.90	−45	500.80 > 348.85	−29
126	Sedaxane	Positive	8.57	332.15 > 159.10	−20	332.15 > 292.10	−16
127	Simazine	Positive	5.25	201.90 > 104.00	−26	201.90 > 132.00	−20
128	Spinosyn-A	Positive	10.88	732.00 > 142.10	−30	732.00 > 98.10	−54
129	Spinosyn-D	Positive	11.72	746.30 > 142.10	−31	746.30 > 98.10	−55
130	Spiromesifen	Positive	13.99	371.20 > 273.30	−14	371.20 > 186.95	−29
131	Sulfoxaflor	Positive	3.82	278.05 > 174.10	−10	278.05 > 154.05	−30
132	Tebuconazole	Positive	10.24	308.10 > 70.05	−22	308.10 > 125.05	−40
133	Tebufenozide	Positive	9.87	353.20 > 133.10	−20	353.20 > 297.10	−10
134	Teflubenzuron	Positive	12.99	380.95 > 141.00	−33	380.95 > 158.05	−18
135	Tetraconazole	Positive	9.09	371.80 > 158.95	−30	371.80 > 70.05	−23
136	Thiacloprid	Positive	3.97	253.00 > 126.10	−22	253.00 > 90.10	−38
137	Thiamethoxam	Positive	3.31	291.70 > 211.00	−13	291.70 > 181.00	−22
138	Tiadinil	Negative	8.48	266.05 > 70.90	22	266.05 > 238.05	11
139	Tolfenpyrad	Positive	13.07	383.70 > 197.00	−25	383.70 > 154.05	−42
140	Trichlorfon	Positive	3.78	256.95 > 109.05	−20	256.95 > 127.05	−21
141	Tricyclazole	Positive	4.30	190.05 > 136.10	−27	190.05 > 163.10	−21
142	Triflumezopyrim	Positive	5.16	399.10 > 278.10	−30	399.10 > 121.10	−37
143	Triforine	Positive	6.77	456.90 > 317.95	−24	456.90 > 68.15	−19
144	Flonicamid	Positive	3.31	230.10 > 203.20	−17	230.10 > 148.00	−27
145	Flonicamid-TFNA-AM	Positive	3.18	191.05 > 148.05	−21	191.05 > 98.15	−31
146	Imidacloprid	Positive	3.52	255.80 > 209.00	−16	255.80 > 175.05	−20
147	Imidacloprid-olefin	Negative	3.36	252.05 > 205.15	11	252.05 > 46.05	33
148	Imidacloprid-urea	Positive	3.53	212.10 > 128.00	−20	212.10 > 126.10	−24
149	Spinetoram-J (XDE-175-J)	Positive	11.84	748.50 > 142.20	−31	748.50 > 98.35	−69
150	Spinetoram-L (XDE-175-L)	Positive	12.66	760.50 > 142.15	−31	760.50 > 98.15	−57

[1] Collision energy.

[2] Precursor ion: $[M+H-CH_3OH]^+$.

Table S5. GC–MS/MS multiple reaction monitoring (MRM) parameters and retention times for the target pesticide analytes.

No.	Analyte	Retention time (min)	Quantifier transition		Qualifier transition	
			Precursor > product (m/z)	CE [1] (eV)	Precursor > product (m/z)	CE (eV)
1	Aldrin	9.83	263.00 > 192.00	40	293.00 > 221.00	20
2	Dieldrin	12.74	263.00 > 193.00	30	279.00 > 242.00	10
3	Atrazine	7.05	215.00 > 200.00	5	217.00 > 202.00	15
4	BHC-α	6.63	181.00 > 145.00	20	219.00 > 146.00	25
5	BHC-β	7.11	181.00 > 145.00	20	219.00 > 146.00	25
6	BHC-γ (Lindane)	7.27	181.00 > 145.00	20	219.00 > 146.00	25
7	BHC-δ	7.85	181.00 > 145.00	20	219.00 > 146.00	25
8	Bifenthrin	16.88	181.00 > 166.00	15	181.00 > 167.00	15
9	Bitertanol	20.29	170.00 > 141.00	20	170.00 > 115.00	40
10	Buprofezin	12.86	172.00 > 131.00	5	172.00 > 139.00	5
11	Chlordane-cis	11.93	373.00 > 264.00	20	375.00 > 301.00	15
12	Chlordane-trans	11.55	375.00 > 266.00	15	373.00 > 264.00	10
13	Oxychlordane	11.01	185.00 > 149.00	5	387.00 > 263.00	10
14	Chlorfenapyr	13.16	364.00 > 282.00	27	364.00 > 248.00	24
15	Chlorfenvinphos-E	10.77	267.00 > 159.00	5	295.00 > 267.00	15
16	Chlorfenvinphos-Z	11.08	267.00 > 159.00	5	295.00 > 267.00	15
17	Chlorobenzilate	13.66	251.00 > 139.00	10	251.00 > 111.00	30
18	Chlorpropham	6.18	213.00 > 171.00	5	213.00 > 127.00	15
19	Chlorpyrifos	9.72	314.00 > 258.00	10	314.00 > 286.00	5
20	Chlorpyrifos-methyl	8.54	286.00 > 93.00	20	286.00 > 271.00	20
21-1	Cyfluthrin-1	21.25	226.00 > 206.00	5	226.00 > 199.00	5
21-2	Cyfluthrin-2	21.46	226.00 > 206.00	5	226.00 > 199.00	5
21-3	Cyfluthrin-3	21.56	226.00 > 206.00	5	226.00 > 199.00	5
21-4	Cyfluthrin-4	21.66	226.00 > 206.00	5	226.00 > 199.00	5
22	Cyhalothrin-γ	18.40	197.00 > 161.00	5	197.00 > 141.00	5
23	Cyhalothrin-λ	18.74	197.00 > 161.00	5	197.00 > 141.00	5
24-1	Cypermethrin-1	21.85	163.00 > 127.00	5	181.00 > 127.00	20
24-2	Cypermethrin-2	22.07	163.00 > 127.00	5	181.00 > 127.00	20
24-3	Cypermethrin-3	22.16	163.00 > 127.00	5	181.00 > 127.00	20
24-4	Cypermethrin-4	22.26	163.00 > 127.00	5	181.00 > 127.00	20
25	Cyprodinil	10.65	224.00 > 208.00	25	224.00 > 118.00	40
26	DDT-p,p'	15.14	235.00 > 165.00	20	235.00 > 199.00	20
27	DDT-o,p'	14.00	235.00 > 165.00	20	235.00 > 199.00	20
28	DDE-p,p'	12.62	246.00 > 176.00	25	318.00 > 247.00	10
29	DDD-p,p'	13.92	235.00 > 165.00	30	199.00 > 163.00	30
30-1	Deltamethrin-1	24.59	252.90 > 93.10	21	252.90 > 172.00	6
30-2	Deltamethrin-2	24.96	252.90 > 93.10	21	252.90 > 172.00	6
31	Dimethenamid	8.42	230.00 > 154.00	10	230.00 > 137.00	20
32	Dimethipin	7.04	118.00 > 58.00	5	118.00 > 90.00	5
33	Diphenylamine	5.95	168.00 > 139.00	30	169.00 > 77.00	35
34	Endosulfan-α	12.30	241.00 > 206.00	15	239.00 > 204.00	20
35	Endosulfan-β	13.70	241.00 > 206.00	15	239.00 > 204.00	20
36	Endosulfan sulfate	14.93	387.00 > 289.00	5	387.00 > 241.00	20

No.	Analyte	Retention time (min)	Quantifier transition		Qualifier transition	
			Precursor > product (m/z)	CE [1] (eV)	Precursor > product (m/z)	CE (eV)
37	Endrin	13.37	262.90 > 191.00	30	263.00 > 193.00	30
38	Endrin-δ-keto	16.43	317.00 > 209.00	35	317.00 > 282.00	10
39	EPN	16.77	169.10 > 140.90	8	169.10 > 77.00	22
40	Ethion	13.94	231.00 > 175.00	10	199.00 > 143.00	15
41	Ethoprophos	5.98	242.00 > 158.00	5	242.00 > 200.00	5
42	Etofenprox	22.66	163.00 > 107.00	20	163.00 > 135.00	10
43	Etoxazole	17.13	302.00 > 141.00	10	302.00 > 274.00	5
44	Fenitrothion	9.30	277.00 > 260.00	10	277.00 > 109.00	10
45	Fenoxanil	13.34	189.00 > 125.00	10	293.00 > 155.00	25
46	Fenpropathrin	17.17	265.00 > 210.00	10	265.00 > 181.00	20
47	Fenthion	9.82	278.00 > 245.00	10	278.00 > 169.00	10
48-1	Fenvalerate/Esfenvalerate-1	23.52	225.00 > 147.00	10	225.00 > 119.00	20
48-2	Fenvalerate/Esfenvalerate-2	23.99	225.00 > 147.00	10	225.00 > 119.00	20
49	Flusilazole	12.82	233.00 > 165.00	10	233.00 > 152.00	10
50	Heptachlor	8.93	272.00 > 237.00	15	272.00 > 235.00	15
51	Isofenphos	10.87	213.00 > 121.00	10	213.00 > 185.00	10
52	Mecarbam	11.13	159.00 > 131.00	5	160.00 > 132.00	10
53	Methacrifos	4.92	208.00 > 180.00	5	240.00 > 180.00	10
54	Methidathion	11.47	145.00 > 85.00	5	145.00 > 58.00	15
55	Parathion	9.91	291.00 > 109.00	10	291.00 > 137.00	10
56	Penthiopyrad	13.85	177.00 > 101.00	20	302.00 > 152.00	5
57	Permethrin-cis	20.17	183.00 > 165.00	10	163.00 > 127.00	10
58	Permethrin-trans	20.43	183.00 > 165.00	10	163.00 > 127.00	10
59	Phenthoate	11.05	274.00 > 246.00	5	274.00 > 121.00	10
60	Phosmet	16.61	160.00 > 77.00	20	160.00 > 133.00	10
61	Phthalide (Fthalide)	10.19	243.00 > 215.00	20	243.00 > 144.00	30
62	Pirimicarb	8.00	238.00 > 166.00	10	238.00 > 96.00	25
63-1	Propiconazole-1	14.92	259.00 > 69.00	10	259.00 > 191.00	10
63-2	Propiconazole-2	15.12	259.00 > 69.00	10	259.00 > 191.00	10
64	Pyridaben	20.62	147.00 > 117.00	20	147.00 > 132.00	10
65	Quinoxyfen	14.93	237.00 > 208.00	25	307.00 > 237.00	25
66	Quintozene	7.21	249.00 > 213.00	15	294.80 > 236.80	16
67	Pentachloroaniline	8.39	263.00 > 192.00	20	267.00 > 194.00	20
68	Spirodiclofen	20.06	312.00 > 109.00	15	312.00 > 259.00	10
69	Terbufos	7.42	231.00 > 129.00	20	231.00 > 175.00	10
70	Thifluzamide	12.72	166.00 > 125.00	15	194.00 > 125.00	20
71	Triadimefon	10.00	208.00 > 181.00	10	208.00 > 127.00	15
72	Triadimenol	11.19	128.00 > 100.00	10	168.00 > 112.00	5
73	Triazophos	14.41	161.00 > 134.00	10	161.00 > 106.00	15
74	Vinclozolin	8.63	285.00 > 212.00	10	287.00 > 214.00	10
75	3,5-dichloroaniline	4.33	161.00 > 99.00	30	163.00 > 90.00	30

[1] Collision energy.
